# Proteomic and Phytohormone Analysis of the Response of Maize (Zea mays L.) Seedlings to Sugarcane Mosaic Virus

**DOI:** 10.1371/journal.pone.0070295

**Published:** 2013-07-23

**Authors:** Liuji Wu, Shunxi Wang, Xiao Chen, Xintao Wang, Liancheng Wu, Xiaofeng Zu, Yanhui Chen

**Affiliations:** 1 Henan Agricultural University and Synergetic Innovation Center of Henan Grain Crops, Zhengzhou, China; 2 Key Laboratory of Physiological Ecology and Genetic Improvement of Food Crops in Henan Province, Zhengzhou, China; 3 Henan Province Seed Control Station, Zhengzhou, China; Cairo University, Egypt

## Abstract

**Background:**

Sugarcane mosaic virus (SCMV) is an important virus pathogen in crop production, causing serious losses in grain and forage yields in susceptible cultivars. Control strategies have been developed, but only marginal successes have been achieved. For the efficient control of this virus, a better understanding of its interactions and associated resistance mechanisms at the molecular level is required.

**Methodology/Principal Findings:**

The responses of resistant and susceptible genotypes of maize to SCMV and the molecular basis of the resistance were studied using a proteomic approach based on two-dimensional polyacrylamide gel electrophoresis (2-DE) and matrix-assisted laser desorption/ionization time of flight mass spectrometry (MALDI-TOF-MS/MS) analysis. Ninety-six protein spots showed statistically significant differences in intensity after SCMV inoculation. The classification of differentially expressed proteins showed that SCMV-responsive proteins were mainly involved in energy and metabolism, stress and defense responses, and photosynthesis. Most of the proteins identified were located in chloroplasts, chloroplast membranes, and the cytoplasm. Analysis of changes in phytohormone levels after virus inoculation suggested that salicylic acid, abscisic acid, jasmonic acid, and azelaic acid may played important roles in the maize response to SCMV infection.

**Conclusions/Significance:**

Among these identified proteins, 19 have not been identified previously as virus-responsive proteins, and seven were new and did not have assigned functions. These proteins may be candidate proteins for future investigation, and they may present new biological functions and play important roles in plant-virus interactions. The behavioural patterns of the identified proteins suggest the existence of defense mechanisms operating during the early stages of infection that differed in two genotypes. In addition, there are overlapping and specific phytohormone responses to SCMV infection between resistant and susceptible maize genotypes. This study may provide important insights into the molecular events during plant responses to virus infection.

## Introduction

The global population has more than doubled, from 3 billion in 1959 to 6.7 billion in 2009 and it is predicted that the human population will reach 8.7 to 11.3 billion by the year 2050. Growth in the global livestock industry has also been continuous over the last two decades [1]. While higher productivity is required to meet the growing demand for food now and in the future, agricultural productivity is seriously constrained by plant diseases [2]. Sugarcane mosaic virus (SCMV) was first reported in Ohio, United States in 1963. It belongs to the sugarcane mosaic subgroup of the Potyviridae, together with maize dwarf mosaic virus, Johnsongrass mosaic virus, sorghum mosaic virus, and Zea mosaic virus. SCMV infects maize, sorghum, sugarcane and other poaceous species throughout the world. Large-scale cultivation of susceptible varieties has facilitated the build-up of SCMV in fields and the dissemination of the virus over large areas in many countries worldwide. Because SCMV is naturally transmitted by aphids in a non-persistent manner [3], the growing of virus-resistant cultivars has been the only effective way to prevent severe losses of both crop yield and quality. Screening of maize germplasm under both greenhouse and field conditions has revealed that only limited inbreds lines are completely resistant to SCMV. While SCMV has been known for a long time, it still remains a threat to the agricultural and livestock industry [4]. Thus, it is important to identify SCMV resistance candidate genes or proteins and obtain information about the molecular mechanisms involved in plant - SCMV interactions.

In the postgenomic era, the transcriptomic strategy has been widely undertaken and contributed greatly to our understanding of global changes in gene expression. Compared with transcript level studies, protein level studies have become progressively more important because it is now well established that proteins are more directly related to function, and there is little correspondence between transcript and protein levels [5], [6]. Proteomics has the potential to reveal the protein expression profiles of organisms in response to pathogen infection or other environmental stresses. The investigation of systemic changes in proteome profiles could be beneficial for elucidating cellular processes involved in viral infections. Recently, a number of proteomics studies using two-dimensional polyacrylamide gel electrophoresis (2-DE) in conjunction with mass spectrometry (MS) have been published and provide a good overview of the proteins present in a given tissue, organelle, or stage of development [7], [8]. However, little is known about SCMV-host interactions from a proteomic perspective.

SCMV is an important virus pathogen especially in maize production [9]. Maize (*Zea mays L.*) is one of the most important crop species, which is used especially for direct human consumption and animal feed. Moreover, maize is an important model organism for biological research and the full genome sequence has been officially published [10]. In the present study, using 2-DE and matrix-assisted laser desorption/ionization time of flight mass spectrometry (MALDI-TOF-MS/MS), we analyzed the proteomic response of resistant and susceptible genotypes of maize to SCMV infection. The results provide an invaluable resource for discovering novel proteins involved in pathogen response, and introducing these proteins into agronomically important species may create resistant crops. In addition, we also analyzed phytohormones changes during SCMV infection. This study will provide further insights into the molecular mechanisms of plant-virus interactions.

## Materials and Methods

### Plant Materials and virus Infection

The maize (*Zea mays* L.) inbred line Siyi (resistant to SCMV) and Mo17 (susceptible to SCMV) were grown in a containment greenhouse under controlled conditions: 24°C, 16-h light/8-h dark photoperiod. The SCMV inoculation mixture was prepared from 100 mg young leaves of SCMV-inoculated susceptible Mo17 adult plants displaying typical mosaic symptoms. The infected leaf tissue was homogenized in 1 ml inoculation buffer (0.01 M phosphate buffer, pH 7.0) and mixed with carborundum. Maize plants were mechanically inoculated with SCMV at the three-leaf growth stage (14 days after sowing) on their lowest two leaves. In parallel, the experimental controls were mock inoculated using inoculation buffer. It was periodically observed for the phenotypic symptoms to examine the occurence of disease. The inoculated leaves were collected from SCMV-inoculated and mock-inoculated plants at 6 dpi and stored at −80°C until analysis. Each sample consisted of pooled leaves derived from six plants, and three biological replicates for each sample were collected.

### Protein Extraction and 2D Electrophoresis

Leaves were ground manually to a fine powder with liquid nitrogen. Then the powder was suspended in 25 mL acetone containing 10% w/v trichloroacetic acid and 65 mM DTT. After being precipitated at −20°C for 1 hour, the pellet was collected by centrifuging at 10000 ×g for 45 min and washed twice with the cold acetone. After air-drying the pellet were dissolved in a lysis buffer containing 9.5 M urea, 4% CHAPS, 2% DTT, 0.5% IPG buffer (GE Healthcare, UK) and 0.1% v/v protease inhibitor cocktail (Merck, Germany). The samples were sonicated three times for 10 s, followed by centrifugation for 45 min at 12, 000 ×g. Protein concentrations in supernatant were determined using the Bio-Rad (Hercules, CA) protein assay reagent.

For 2-DE, 100 µg and 400 µg proteins were loaded onto analytical and preparative gels, respectively. The Ettan IPGphor Isoelectric Focusing System (Amersham Biosciences) and pH 3–10 immobilized pH gradient (IPG) strips (13 cm, nonlinear; Amersham) were used for isoelectric focusing (IEF). The IPG strips were rehydrated for 12 h in 250 µL rehydration buffer containing the protein samples. IEF was performed in four steps: 30 V for 12 h, 500 V for 1 h, 1000 V for 1 h, and 8000 V for 8 h. The gel strips were equilibrated for 15 min in equilibration buffer (50 mM Tris-HCl (pH 8.8), 6 M urea, 2% SDS, 30% glycerol, and 1% DTT). This step was repeated using the same buffer with 4% iodoacetamide in place of 1% DTT. The strips were then subjected to the second-dimensional electrophoresis after transfer onto 12.5% SDS-polyacrylamide gels. Electrophoresis was performed using the Hofer SE 600 system (Amersham) at 15 mA per gel for 30 min, followed by 30 mA per gel until the bromophenol blue reached the end of the gel.

### Gel Staining and Image Analysis

Protein spots in the analytical and preparative gels were visualized by silver staining, which is more sensitive than Coomassie staining, according to a previously reported method [11]. The stained gels were scanned using UMax Powerlook 2110XL (UMax), and image analysis was accomplished using Imagemaster 2D Platinum software (GE Amersham) with the following parameters for spot detection: smooth, 2; min area, 5; saliency, 1. The quantitative measure of spot matching success and method of normalization are in accordance with the manual of ImageMasterTM 2D Platinum software. SPSS 16.0 statistical software was used for ANOVA test analysis. Quantitative comparisons between two sample sets were carried out taking into account statistically significant spots only with p<0.05. The proteins with a 2 fold or greater overlap ratio threshold filtering were considered as differentially expressed.

### Tryptic Digestion

Tryptic digestion was performed according to the method reported previously [12]. Protein spots were excised manually from the preparative gels, destained with 100 mM NH_4_HCO_3_ in 30% ACN. After removing the destaining buffer, the gel plugs were lyophilized and rehydrated in 30 µL of 50 mM NH4HCO3 containing 50 ng trypsin (Promega, USA). After overnight digestion at 37°C, the peptides were extracted three times with 0.1% TFA in 60% ACN. Extracts were pooled together and lyophilized. The resulting lyophilized tryptic peptides were kept at −80°C until mass spectrometric analysis.

### MALDI-TOF - MS Analysis and Database Searching

MS and MS/MS spectra were obtained using the ABI MALDI-TOF/TOF 4800 Proteomics Analyzer (Applied Biosystems, USA). Peptide mass maps were acquired in positive ion reflector mode (20 kV accelerating voltage) with 1000 laser shots per spectrum. Monoisotopic peak masses were automatically determined within the mass range 800–4000 Da with a signal to noise ratio minimum set to 10 and a local noise window width of m/z 250. For one main MS spectrum 20 subspectra with 25 shots per subspectrum were accumulated using a random search pattern. Ten of the most intense ion signals were selected as precursors for MS/MS acquisition, excluding the trypsin autolysis peaks and the matrix ion signals.

The MS together with MS/MS spectra were searched against the NCBI viridiplantae database using the GPS Explorer software, version 3.6 (Applied Biosystems) and MASCOT version 2.1 (Matrix Science) with the following parameter settings: trypsin cleavage, one missed cleavage allowed, carbamidomethylation set as fixed modification, oxidation of methionines allowed as variable modification, peptide mass tolerance set to 100 ppm, fragment tolerance set to±0.3 Da, and minimum total ion score confidence interval for MS/MS data set to 95%.

### Western Blot Analysis

For each of the four maize sample groups, the extracted total proteins were separated on 12% SDS/PAGE gels and then transferred onto a polyvinylidene difluoride (PVDF) membrane using an electrophoretic transfer system (Bio-Rad, USA). Membranes were blocked for 1 h at room temperature with 5% skim milk in PBST and probed with rabbit polyclonal antibody to ascorbate peroxidase (Agrisera, Sweden), rabbit polyclonal antibody to peroxiredoxin (Agrisera, Sweden), rabbit polyclonal antibody to superoxide dismutase (Agrisera, Sweden), and mouse monoclonal antibody to actin (Abmart, China) at 4°C overnight respectively. Then the membranes were incubated with horseradish peroxidase (HRP) conjugated goat antirabbit IgG or goat antimouse IgG (Boshide, China) for 1 h at room temperature. Immunoreactivity was detected with an HRP-DAB Detection Kit (Tiangen, China).

### Bioinformatics

The molecular functions of the identified proteins were classified according to the gene ontology annotation combined with the biology function as described by Bevan [13]. The subcellular locations of the unique proteins identified in this study were determined using Softberry bioinformatics software. Genetic map positions were determined *in silico* using the Maize GDB http://www.maizegdb.org/. The protein-protein interaction network was analyzed by String software.

### Phytohormones Extraction and Analysis

Leaves were collected from SCMV-inoculated plants and mock-inoculated plants at 2, 4, 6, 8, 10, and 12 days post inoculation. Leaf tissues were homogenized and extracted in 80% aqueous methanol according to the method described in Escalante-Pérez et al [14]. Extracts were passed through a Sep Pak C18 cartridge. Methanol was removed under reduced pressure, and the aqueous residue was partitioned three times against ethyl acetate (pH 3.0). The ethyl acetate was removed from the combined organic fractions under reduced pressure. The newly obtained residue was resuspended in Tris-buffered saline (TBS; 150 mmol/l NaCl 1 mmol/L MgCl2, and 50 mmol/L Tris at pH 7.8). Then, the levels of salicylic acid (SA), abscisic acid (ABA), ethylene (ET), jasmonic acid (JA), and azelaic acid (AZA) were assayed using enzyme linked immunosorbent assay (ELISA) test kits (Hermes Criterion Biotechnology, Canada) according to the manufacturer’s instructions.

## Results and Discussion

### Phenotypic Comparison of the Resistant and Susceptible Maize in Response to SCMV Infection

**Figure 1:**
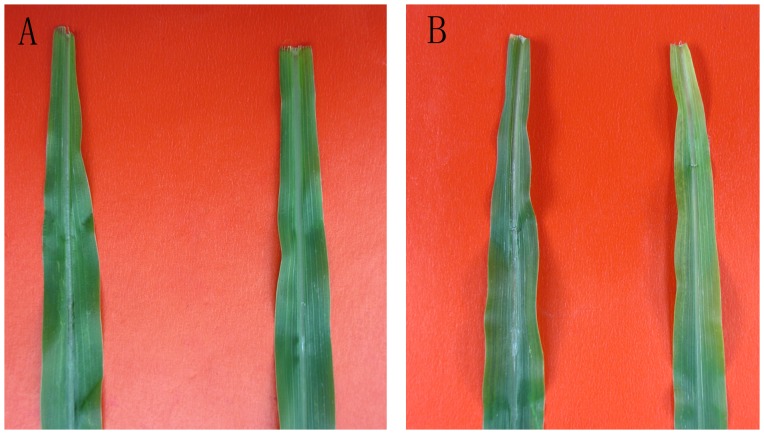
Phenotypic analysis of resistant Siyi and susceptible Mo17 plants to SCMV infection. The plant phenotypes were observed at six days post inoculation. (A) Siyi plants: left, mock-inoculated; right, SCMV-inoculated. (B) Mo17 plants: left, mock-inoculated; right, SCMV-inoculated. Typical mosaic symptoms are visible in Mo17 with SCMV infection, while Siyi is symptomless.

To monitor the protein changes of maize in response to SCMV infection, an inoculation assay was established for resistant maize genotype Siyi and susceptible maize genotype Mo17. Maize plants were periodically examined for the phenotypic symptoms which indicated the occurence of disease. At six days post inoculation (dpi), the mosaic symptoms began to appear in susceptible maize plants Mo17 with SCMV inoculation (Some irregular, light mosaic or mottle was found along the veins), while the resistant maize genotype Siyi was symptomless (Figure 1). At this stage, the bottom inoculated leaves of both Siyi and Mo17 plants were collected for further analysis. In parallel, as experimental controls, Siyi and Mo17 plants were mock inoculated with inoculation buffer and also collected at six dpi. In addition, when symptom progression was monitored over a prolonged period of time (30 dpi), all Mo17 plants with SCMV inoculation showed severe symptoms (The leaves become more yellow and maize plant become severe stunting), while Siyi plants were symptomless throughout the same time period (data not shown).

### 2-DE Analysis of Protein Expression in Response to SCMV Infection

**Figure 2:**
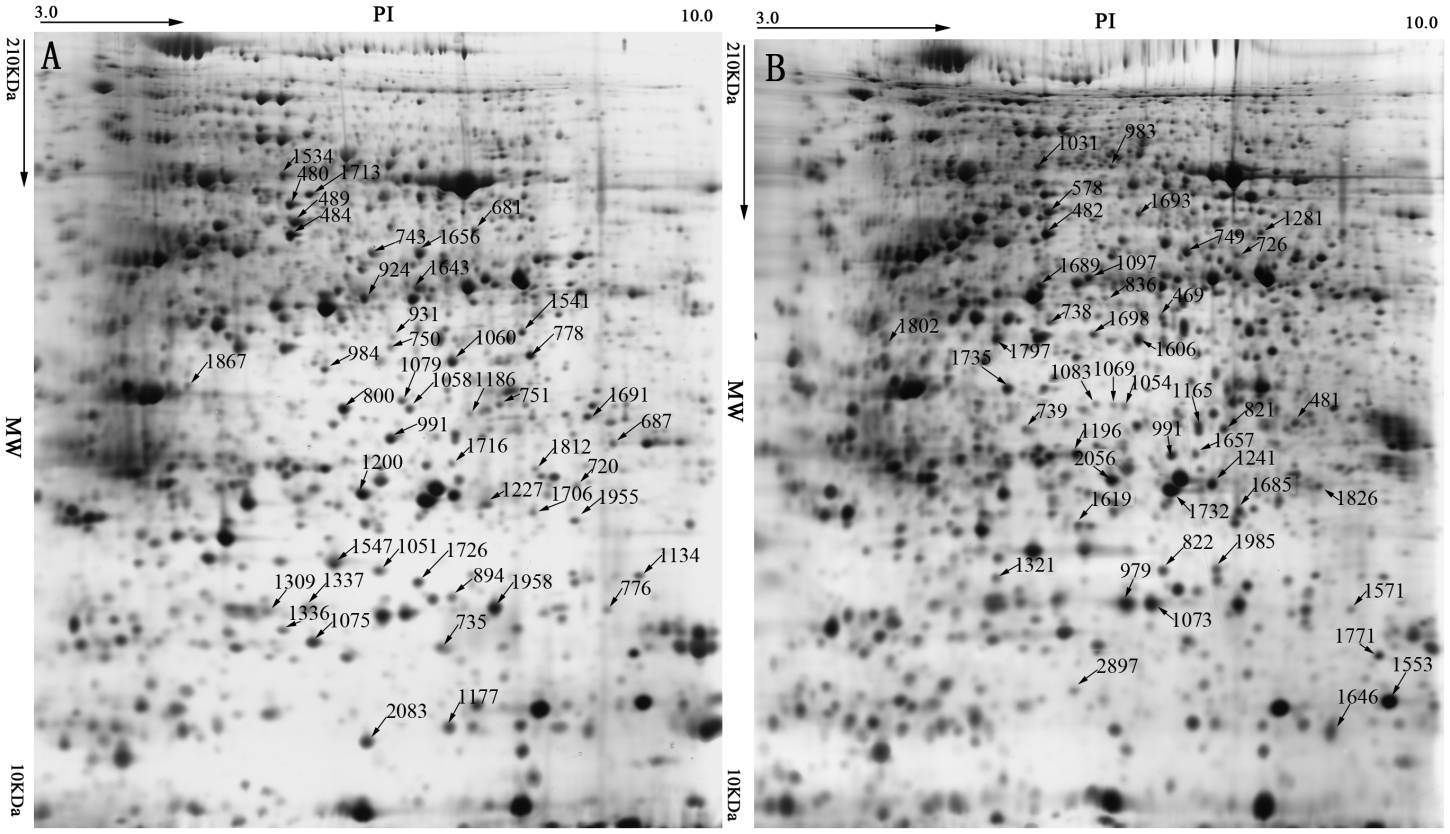
Differentially expressed maize leaf protein spots observed by 2-DE analysis. Arrows indicate spots showing significantly regulated proteins in the resistant genotype Siyi (A) and susceptible genotype Mo17 (B). The numbers correspond to those in Tables 1 and 2, respectively.

To examine the protein expression patterns, maize leaves were collected in triplicates from four sample groups (Siyi_SCMV_, Siyi_CK,_ Mo17_SCMV_ and Mo17_CK_). Before the formal 2-DE experimental, the optimal time point for sample collection was determined by SDS-PAGE analysis. From this assay, it was established that major differences in the protein accumulation pattern from inoculated leaves were firstly observed at 6 dpi in protein extracts (Figure S1). Therefore, for subsequent 2-DE analysis, protein extracts from 6 dpi leaves were used. Based on 2-DE analysis, a total of 96 protein spots showed a statistically significant (p<0.05) change in intensity (ratio ≥2). The positions of the protein spots in the representative 2-DE images are shown in Figure 2. Among these 96 protein spots, 49 appeared to be modulated in the SCMV resistant maize group (Siyi_SCMV_/Siyi_CK_), and of the modulated proteins, 29 protein spots were upregulated while the other 20 downregulated. In the susceptible maize group (Mo17_SCMV/_Mo17_CK_), 47 protein spots appeared to be altered, and of the modulated proteins, 24 protein spots were up-regulated while the other 23 downregulated.

### Identification and Analysis of the Differentially Expressed Proteins

The proteins detected as differentially expressed were excised from preparative gels and subjected to digestion with trypsin for identification and further characterization. Among the 96 differentially expressed protein spots, 90 were successfully analyzed by MALDI-TOF-MS/MS (The detailed description of MS/MS analysis results can be found in Table S1 and the raw data of peptide identification is available at the website http://foodcrop.henau.edu.cn/article/2013/0418/article_101.html). Of the proteins identified, 83 proteins (92%) had known functions or sequences similar to those of known proteins, whereas seven proteins (8%) were novel and had not been assigned any functions (Table 1 and 2).

**Table 1:** List of proteins identified by MALDI-TOF-MS/MS analysis in resistant maize genotype Siyi’s response to SCMV infection.

Spot ID^a^	Accession No^b^	Protein name	Theoretical MW/pI^c^	NP^d^	Cverage (%)	Protein score	Fold change^e^	*P* value^f^	Function	Sub-cellular localization^g^
687	gi|195622012	Membrane-associated protein	35059.3/9.5	9	26	270	−2.27	0.037	Transport protein	Chloroplast
1534	gi|304651309	ZYP1 protein	101020.6/5.92	19	20	92	3.61	0.017	Cell growth	Nuclear
1541	gi|195612242	Cytochrome P450	59241.8/8.96	11	9	111	10.81	0.026	Stress and defense response	Nuclear
1547	gi|195622322	Glutathione S-transferase	23879.7/5.96	8	42	189	−3.95	0.041	Stress and defense response	Chloroplast
1656	gi|195607124	Calcium-dependent protein kinase	61156.1/6.28	4	14	82	24.39	0.022	Signal transduction/transcription	Plasma membrane
1691	gi|226507468	bZIP transcription factor ABI5	38416.8/9.56	9	16	97	3.49	0.01	Signal transduction/transcription	Nuclear
1713	gi|226507400	Aconitase	99565.3/6.04	23	26	321	4.34	0.038	Energy and metabolism	Cytoplasmic
1726	gi|195619268	Peroxiredoxin	23917.6/7.74	5	16	110	3.62	0.034	Stress and defense response	Membrane bound Chloroplast
1075	gi|326378667	Histidine triad nucleotide binding protein	15250.6/6.4	4	24	124	2.16	0.048	Stress and defense response	Cytoplasmic
1955	gi|195637254	Nucleoside diphosphate kinase	24181.7/9.51	12	45	355	6.95	0.011	Energy and metabolism	Membrane bound Chloroplast
1958	gi|195640294	Thioredoxin	21062.1/8.88	3	11	78	−4.79	0.019	Stress and defense response	Membrane bound Chloroplast
2083	gi|195655243	Abscisic stress ripening protein	11666.8/6.81	5	65	234	5.56	0.037	Stress and defense response	Nuclear
1643	gi|14276718	T-cytoplasm male sterility restorer factor	59772.9/6.9	21	38	886	2.8	0.023	Cell growth	Mitochondrial
720	gi|226502949	60S ribosomal protein	34461.8/9.33	10	26	96	2.69	0.012	Structure protein	Endoplasmic reticulum
751	gi|195625588	Cysteine synthase	40833.3/8.74	11	23	187	2.66	0.032	Energy and metabolism	Membrane bound Chloroplast
1227	gi|195643284	Chaperonin	25750/7.74	4	22	140	2.6	0.058	protein synthesis and folding	Membrane bound Chloroplast
1060	gi|195627092	Glutamine synthetase	47192/7.64	10	38	210	−2.52	0.028	Energy and metabolism	Membrane bound Chloroplast
1812	gi|226496743	50S ribosomal protein	37235.2/8.69	16	48	616	−2.68	0.047	Structure protein	Chloroplast
484	gi|28948384	Transketolase	73346.7/5.47	29	47	713	6.61	0.025	Energy and metabolism	Membrane bound Chloroplast
489	gi|28948384	Transketolase	73346.7/5.47	32	55	772	−3.23	0.012	Energy and metabolism	Membrane bound Chloroplast
480	gi|75140229	Transketolase	73346.7/5.47	31	58	889	2.8	0.025	Energy and metabolism	Membrane bound Chloroplast
743	gi|359497202	T-complex protein	61032.7/6.2	9	17	86	2.44	0.049	Signal transduction/transcription	Cytoplasmic
681	gi|343227637	Beta-D-glucosidase precursor	63463.4/6.75	19	26	585	4.79	0.047	Energy and metabolism	Membrane bound Chloroplast
924	gi|3694807	Alanine aminotransferase	53534.3/6.3	10	21	215	−2.39	0.042	Stress and defense response	Cytoplasmic
1867	gi|50952841	RuBisCo subunit binding-protein beta subunit	44275/4.75	14	44	450	−5.14	0.011	Photosynthesis	Membrane bound Chloroplast
750	gi|11467200	RuBisCo large subunit	53294/6.33	15	31	289	−2.14	0.039	Photosynthesis	Cytoplasmic
931	gi|11467200	RuBisCo large subunit	53294/6.33	27	50	950	3.59	0.031	Photosynthesis	Cytoplasmic
984	gi|162464489	Glutamate dehydrogenase	44279.8/6.09	11	26	172	3.32	0.039	Energy and metabolism	Mitochondrial
991	gi|195612198	Fructose-bisphosphate aldolase	38464.1/6.26	15	47	683	−3.21	0.042	Energy and metabolism	Cytoplasmic
1058	gi|195634659	Fructose-bisphosphate aldolase	41923.5/7.63	17	47	864	−3.66	0.044	Energy and metabolism	Membrane bound Chloroplast
1079	gi|195634659	Fructose-bisphosphate aldolase	41923.5/7.63	17	44	915	4.55	0.049	Energy and metabolism	Membrane bound Chloroplast
1177	gi|194032833	Defensin	12071.7/7.48	8	43	82	5.56	0.024	Stress and defense response	Extracellular
1716	gi|149392465	Ferredoxin-NADP reductase	37878/8.37	6	78	131	−2.57	0.018	Photosynthesis	Membrane bound Chloroplast
1186	gi|195624056	Ferredoxin-NADP reductase	40976.5/8.53	10	26	111	4.22	0.032	Photosynthesis	Membrane bound Chloroplast
1051	gi|195627890	Stress responsive protein	23158.2/6.95	3	13	72	3	0.035	Stress and defense response	Cytoplasmic
1337	gi|195628632	Remorin	21875.4/5.74	9	21	111	9.44	0.073	Stress and defense response	Plasma membrane
1309	gi|195628632	Remorin	21875.4/5.74	5	21	98	−4.72	0.023	Stress and defense response	Plasma membrane
1336	gi|195628632	Remorin	21875.4/5.74	8	32	179	21.63	0.019	Stress and defense response	Plasma membrane
894	gi|226532728	Electron transporter protein	21197.7/7.57	3	25	89	2.26	0.024	Electron transport	Plasma membrane
1200	gi|194707256	Unknown	33565.8/5.96	10	34	462	−4.52	0.034	Unknown	Extracellular
778	gi|224028705	Unknown	51877.9/8.35	21	41	511	3.55	0.038	Unknown	Membrane bound Chloroplast
800	gi|195636170	Unknown	42290.4/6.53	21	36	584	−4.66	0.046	Unknown	Membrane bound Chloroplast
1706	gi|194690760	Unknown	24914.4/9.18	7	22	116	−3.33	0.019	Unknown	Membrane bound Chloroplast
735	gi|20257673	Glycine-rich RNA binding protein	14201.5/6.89	7	48	85	2.39	0.012	Protein synthesis and folding	Chloroplast
1134	gi|195633817	Oxygen-evolving enhancer protein 3	23104.5/9.77	8	45	267	−2.11	0.11	Photosynthesis	Membrane bound Chloroplast
776	gi|195633817	Oxygen-evolving enhancer protein 3	23104.5/9.77	7	42	234	−2.5	0.023	Photosynthesis	Membrane bound Chloroplast

a The spot ID was determined at the beginning of the analysis of the gel.

b Accession number from NCBI database of the matched protein.

c Theoretical MW/pI was calculated using DNAStar software.

d NP: the number of matched peptides.

e Fold change was calculated by imagemaster 2D software.

f P value obtained from ANOVA test analysis.

g Map positions were determined *in silico* using the Maize GDB.

**Table 2:** List of proteins identified by MALDI-TOF-MS/MS analysis in susceptible maize genotype Mo17’s response to SCMV infection.

Spot ID^a^	Accession No^b^	Protein name	Theoretical MW/pI^c^	NP^d^	Coverage %	Protein score	Fold change^e^	*P* value	Function^f^	Sub-cellular localization^g^
1165	gi|195624056	Ferredoxin-NADP reductase	40976.5/8.53	10	26	111	3.48	0.049	Photosynthesis	Membrane bound Chloroplast
1281	gi|212274863	Exoglucanase1	67326.3/6.92	12	19	83	5.16	0.046	Energy and metabolism	Membrane bound Chloroplast
1321	gi|195628632	Remorin	21875.4/5.74	5	39	98	−5.9	0.012	Stress and defense response	Plasma membrane
1553	gi|8118441	Germin-like protein	10068.3/9.99	4	35	113	4.65	0.049	Stress and defense response	Extracellular
1571	gi|195639710	Ubiquitin fusion protein	14977.1/9.94	3	16	70	−3.25	0.024	Signal transduction/transcription	Nuclear
1802	gi|50952841	RuBisCo subunit binding-protein beta subunit	44275/4.75	14	44	450	−3.75	0.036	Photosynthesis	Membrane bound Chloroplast
1619	gi|162460411	Glutathione S-transferase	23891.6/6.05	5	29	100	−7.34	0.043	Stress and defense response	Chloroplast
1646	gi|109892850	Cytochrome c oxidase subunit	1707/9.63	2	100	74	−7.77	0.048	Energy and metabolism	Chloroplast
1732	gi|195619268	Peroxiredoxin-5	23917.6/7.74	5	20	110	3.02	0.037	Stress and defense response	Membrane bound Chloroplast
1685	gi|357123797	Peroxiredoxin-2E-1	23757.5/8.58	5	20	83	3.55	0.02	Stress and defense response	Membrane bound Chloroplast
1689	gi|195628730	2,3-bisphosphoglycerate-independent phosphoglycerate mutase	60810.9/5.29	19	30	485	3.65	0.02	Energy and metabolism	Cytoplasmic
1693	gi|226507400	Aconitase	99565.3/6.04	23	6	321	3.68	0.043	Energy and metabolism	Cytoplasmic
882	gi|226532728	Electron transporter protein	21197.7/7.57	3	25	89	2.47	0.033	Electron transport	Plasma membrane
1073	gi|326378667	Histidine triad nucleotide binding protein	15250.6/6.4	4	24	124	2.47	0.033	Stress and defense response	Cytoplasmic
1826	gi|195637254	Nucleoside diphosphate kinase	24181.7/9.51	12	45	355	5.53	0.028	Energy and metabolism	Membrane bound Chloroplast
1985	gi|195640294	Thioredoxin	21062.1/8.88	3	11	78	−5.98	0.033	Stress and defense response	Membrane bound Chloroplast
2056	gi|226530305	Ascorbate Peroxidase	27461.9/5.55	8	35	110	−3.13	0.011	Stress and defense response	Cytoplasmic
2897	gi|195655243	Abscisic stress ripening protein	11666.8/6.81	5	65	234	5.27	0.039	Stress and defense response	Nuclear
482	gi|28948384	Transketolase	73346.7/5.47	29	47	713	6.56	0.047	Energy and metabolism	Membrane bound Chloroplast
578	gi|28948384	Transketolase	73346.7/5.47	32	55	772	−3.29	0.047	Energy and metabolism	Membrane bound Chloroplast
749	gi|359497202	T-complex protein	61032.7/6.2	9	6	86	2.44	0.049	Signal transduction/transcription	Cytoplasmic
738	gi|11467189	ATP synthase CF1 alpha subunit	55729.4/5.87	28	43	913	2.72	0.038	Energy and metabolism	Membrane bound Chloroplast
836	gi|11467189	ATP synthase CF1 alpha subunit	55729.4/5.87	25	49	695	−2.04	0.026	Energy and metabolism	Membrane bound Chloroplast
1097	gi|11467189	ATP synthase CF1 alpha subunit	55729.4/5.87	21	41	856	2.29	0.014	Energy and metabolism	Membrane bound Chloroplast
726	gi|343227637	Beta-D-glucosidase precursor	63463.4/6.75	19	26	585	5.04	0.045	Energy and metabolism	Membrane bound Chloroplast
979	gi|162462586	Superoxide dismutase [Cu-Zn]	15193.4/5.46	6	64	134	−3.79	0.044	Stress and defense response	Cytoplasmic
991	gi|195612198	Fructose-bisphosphate aldolase	38464.1/6.26	15	47	683	−3.38	0.011	Energy and metabolism	Cytoplasmic
1054	gi|195634659	Fructose-bisphosphate aldolase	41923.5/7.63	19	46	806	−4.41	0.039	Energy and metabolism	Membrane bound Chloroplast
1069	gi|195634659	fructose-bisphosphate aldolase	41923.5/7.63	17	47	864	−5.32	0.047	Energy and metabolism	Membrane bound Chloroplast
1083	gi|195634659	Fructose-bisphosphate aldolase	41923.5/7.63	17	44	915	5.83	0.046	Energy and metabolism	Membrane bound Chloroplast
1698	gi|11467200	RuBisCo large subunit	53294.6/6.33	29	55	1010	−2.89	0.046	Photosynthesis	Cytoplasmic
1606	gi|11467200	RuBisCo large subunit	53294.6/6.33	7	13	131	−4.45	0.013	Photosynthesis	Cytoplasmic
469	gi|11467200	RuBisCo large subunit	53294.6/6.33	28	57	1180	2.64	0.028	Photosynthesis	Cytoplasmic
1241	gi|195623400	Chaperonin	25558.7/8.67	10	54	235	2.83	0.027	protein synthesis and folding	Membrane bound Chloroplast
983	gi|357151031	Disease resistance RPP8-like protein	154698.7/5.6	18	17	75	2.66	0.033	Stress and defense response	Membrane bound Gogli
1797	gi|226502947	S-adenosylmethionine synthetase	42985.6/5.5	14	41	699	−2.56	0.016	Energy and metabolism	Cytoplasmic
821	gi|195625588	Cysteine synthase	40833.3/8.74	11	23	187	−2.76	0.011	Energy and metabolism	Membrane bound Chloroplast
739	gi|226508112	Cysteine synthase	41839.6/6.97	8	28	394	−2.78	0.042	Energy and metabolism	Membrane bound Chloroplast
1031	gi|219819651	Pyruvate orthophosphate dikinase	100312.9/5.55	14	12	191	2.2	0.046	Photosynthesis	Cytoplasmic
1735	gi|226500452	Protochlorophyllide reductase	42434.9/8.96	7	21	203	−2.35	0.024	Photosynthesis	Membrane bound Chloroplast
1657	gi|126116602	Coat protein	31744/7.18	6	15	95	43.24	0.037	Virus protein	Virus protein
1196	gi|194707256	Unknown	33565.8/5.96	10	34	462	−5.15	0.025	Unknown	Extracellular
481	gi|212722236	Unknown	41352.4/8.89	13	42	137	−2.74	0.036	Unknown	Membrane bound Chloroplast
1771	gi|194705220	Unknown	10854.7/9.88	12	77	396	−2.1	0.012	Unknown	Chloroplast

a The spot ID was determined at the beginning of the analysis of the gel.

b Accession number from NCBI database of the matched protein.

c Theoretical MW/pI was calculated using DNAStar software.

d NP, the number of matched peptides.

e Fold change was calculated by imagemaster 2D software.

f P value obtained from ANOVA test analysis.

g Map positions were determined *in silico* using the Maize GDB.

**Figure 3:**
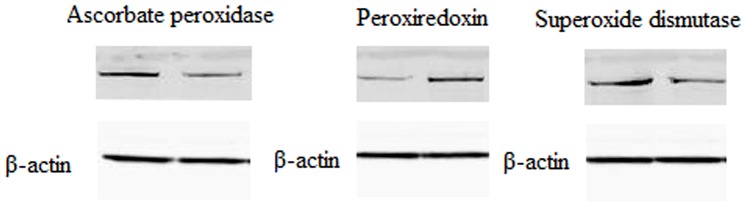
Western blot analysis of ascorbate peroxidase, peroxiredoxin and superoxide dismutase levels in SCMV-inoculated and mock-inoculated samples. Expression level of β-actin was used as loading control. The changes in these three proteins were in good agreement with the proteomic results. These experiments were repeated two times, with similar results.

Among these differentially expressed proteins, ascorbate peroxidase, peroxiredoxin and superoxide dismutase are important proteins associated with disease and stress signals. To verify the data from proteomics experiments, the three proteins were selected and further tested via western blot analysis (Figure 3). Ascorbate peroxidase was significantly downregulated in the Mo17_SCMV_ group when compared with Mo17_CK_ group (control). Similar changes were also observed for superoxide dismutase. In contrast, the expression level of peroxiredoxin was significantly upregulated in the Siyi_SCMV_ group when compared with Siyi_CK_ group (control). The western blot results for the three proteins were consistent with the proteomics analysis, thus strongly supporting the reliability of the proteomics methods.

Several proteins were present in multiple spots in our study. For the differentially expressed proteins from the SCMV resistant maize group (Siyi_SCMV_/Siyi_CK_), three protein spots 484, 489 and 480, two protein sports 750 and 931, three protein spots 991, 1058 and 1079, two protein spots 1716 and 1816, two protein sports 1134 and 776, respectively, were identified as the same protein, transketolase, ribulose-1,5-bisphosphate carboxylase/oxygenase (RuBisCO) large subunit, fructose-bisphosphate aldolase, ferredoxin-NADP reductase, remorin and oxygen-evolving enhancer protein (Table 1). For the susceptible maize group (Mo17_SCMV/_Mo17_CK_), two protein spots 1666 and 739, three protein spots 1698, 1606 and 469, two protein spots 482 and 578, three protein spots 738, 836 and 1097, respectively, were identified as the same protein, cysteine synthase, RuBisCO large subunit, transketolase, ATP synthase CF1 alpha subunit (Table 2). These results suggest that these differentially expressed proteins are present in different isoforms or have undergone posttranslational modifications.

Interestingly, the same protein could be represented by different protein spots in the 2D gels and showed opposite expression patterns. For example, ferredoxin-NADP reductase, a SCMV responsive protein in Siyi, was represented by two protein spots in 2D gels (1716 and 1186). However, 1716 was down-regulated while 1186 was up-regulated. This phenomenon was also found for ATP synthase CF1 alpha subunit (spots 738, 836 and 1097), a SCMV responsive protein in Mo17 and belongs to the energy and metabolism category. One hypothesis concerning this phenomenon is that in plant the same protein may have different isoforms, which play different roles during virus infection. These results also indicate the complex regulatory mechanisms of plants in response to viral infections.

### Functions of the Identified Proteins and their Relationship to the Study

**Figure 4:**
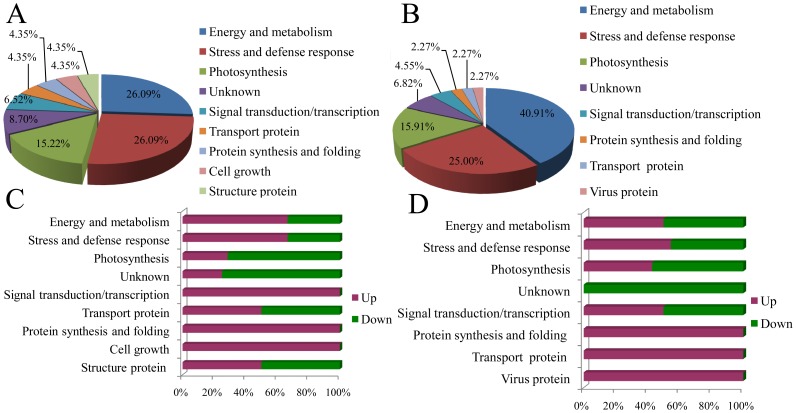
Distribution of functional categories of differentially expressed proteins. (A) Functional categories of differentially expressed proteins in Siyi. (B) Functional categories of differentially expressed proteins in Mo17. (C) Contributions to molecular functions from up-(red) and down-(green) regulated proteins in Siyi. (D) Contributions to molecular functions from up-(red) and down-(green) regulated proteins in Mo17.

The differentially expressed proteins identified from the SCMV-resistant (Siyi_SCMV_/Siyi_CK_) and the susceptible maize (Mo17_SCMV/_Mo17_CK_) genotypes were functionally classified into nine and eight groups, respectively (Figure 4A and B). The largest groups of proteins showing differential expression between Siyi_SCMV_ and Siyi_CK_ were the energy and metabolism group and the stress and defense response group (26.09%), followed by photosynthesis (15.22%). The fourth group represented proteins is unknown proteins (8.7%) followed by signal transduction/transcription (6.52%). The remaining proteins were classified into transport, protein synthesis and folding, structural protein, and cell growth (each with 4.35%). For differentially expressed proteins between Mo17_SCMV_ and Mo17_CK_, energy and metabolism again represents the largest group (40.91%), followed by stress and defense response (25%). The third group represented proteins involved in photosynthesis (15.91%) followed by unknown proteins (6.82%) and signal transduction/transcription proteins (4.55%). The remaining proteins belong to the protein synthesis and folding, transport and viral protein (2.27% each).

**Figure 5:**
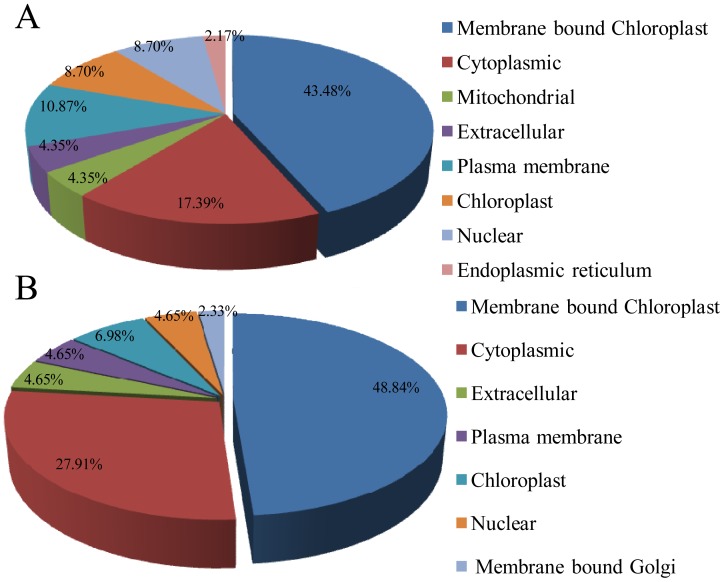
Subcellular locations of differentially expressed proteins. (A) Subcellular location classification of differentially expressed proteins in Siyi. (B) Subcellular location classification of differentially expressed proteins in Mo17.

The distribution of up- and downregulated proteins in the different functional groups is shown in Figure 4C and D. Bioinformatic analysis by Softberry revealed that the majority of the identified proteins were located in the chloroplast membranes, the cytoplasm, and chloroplasts (Figure 5A and B). These results suggest that SCMV infection influenced these physiological processes in both the resistant Siyi and the susceptible Mo17. However, in terms of the specific functional groups, there were different proportions of upregulated and downregulated proteins in Siyi and Mo17.

#### Energy and metabolism regulation under SCMV infection

In plants, pathogenic infections often induce some common physiological alterations and the most important metabolic perturbations [15]. In our study, enzymes involved in the primary metabolism were found to be differentially regulated in response to SCMV infection. In addition, this study showed a marked increase of energy and metabolism-related proteins in the resistant maize genotype Siyi compared with the susceptible Mo17. A number of proteins that play important roles in sugar metabolic pathways were also up-or down-regulated after SCMV infection. For example, fructose-bisphosphate aldolase is a glycolytic enzyme that catalyses the reversible aldol cleavage or condensation of fructose-1,6-bisphosphate into dihydroxyacetone-phosphate and glyceraldehyde 3-phosphate [16]. It showed significant variations between virus-infected and normal plants. Differential accumulations of these proteins implied a shift in the sugar metabolic direction after SCMV inoculation.

Transketolase is an enzyme involved in the pentose phosphate pathway, which is an important source of reductant and carbohydrate molecules with different structures. In our study, transketolase was represented by different protein spots in the 2D gels. The abundance of transketolase in the leaves of plants with SCMV inoculations showed different expression patterns both in Siyi (480 and 484 were increased, while 489 was decreased) and Mo17 (482 increased, while 578 decreased), suggesting that transketolase may have different isoforms and play different roles during viral infection. The decrease of this enzyme suggested that pentose phosphate pathway in plants after SCMV inoculation might be weakened, which is a disadvantage since the plant cell requires intermediary products of sugar metabolism and reductant for growth and development [17]. However, the functional significance of the increase of this enzyme is unclear.

#### SCMV infection altered the expression of stress and defense related proteins

A high proportion of the proteins indentified in this work were stress related, with many of them potentially involved in counteracting stress. Several were represented more significantly in the resistant genotype Siyi. Among these proteins, alanine aminotransferase was downregulated in Siyi after SCMV inoculation. Previous studies have demonstrated that an alanine aminotransferase- like protein functions as a photorespiratory glyoxylate aminotrasferase (GGAT). Photorespiratory transamination to glyoxylate, which is mediated by GGAT and serine glyoxylate aminotransferase, appeared to play an important role in plant growth and in the regulation of amino acid metabolism [18], [19]. Recently, it was reported that alanine aminotransferase might be synthesized almost exclusively in the cytosol of virus-infected leaves, being involved in the defense response against locally invading pathogens. In fact, it was observed that the expression of CaAlaAT1 gene, which encodes a putative alanine aminotransferase, was dramatically induced in hot pepper plants (Capsicum annuum L. cv. Bugang) during the incompatible interaction with TMV [20]. However, we observed a downregulation of alanine aminotransferase in this study, which indicated that the defense response is more complex than a simple upregulation or downregulation of all potential defense proteins.

It is well known that an early response to pathogen attack is oxidative burst, which leads to the production of reactive oxygen species (ROS) [21]. Both superoxide dismutase (SOD) and ascorbate peroxidase are ROS-related proteins. As an antioxidant protein and a key enzyme for detoxifying ROS in plant cells, SOD has a direct role in conferring resistance to a wide range of pathogens [22], [23]. Ascorbate peroxidase is a major H_2_O_2_ scavenging enzyme associated with different stress signals. In normal physiological conditions, ascorbate peroxidase can act as a scavenging enzyme that removes ROS. The ROS concentration has to be kept at sufficiently low levels to prevent large-scale cellular oxidative damage [24], [25]. In our study, we observed a decrease of both SOD and ascorbate peroxidase in the susceptible genotype Mo17. This result suggests that one possible infection model in which SCMV suppresses the H_2_O_2_-degrading activity of ascorbate peroxidase and the antioxidant activity of SOD, thereby leading to an increase of H_2_O_2_ and ROS expression. The higher expression levels of H_2_O_2_ and ROS may then serve as second messengers to inactivate the expression of plant defense-related genes and weaken mechanical barriers.

#### SCMV infection on photosynthesis

In plants, photosynthesis occurs mainly within the leaves, and it is a process in which light energy is converted to chemical energy and used to produce organic compounds. Several studies have revealed that virus-infected plants are generally characterized by a decreased photosynthetic rate suggesting that photosynthesis is one of the major repressed activities during host defense responses [26], [27]. In our study, all of the photosynthesis-related proteins were downregulated in the resistant maize Siyi except one of the isoforms of RuBisCO large subunit and ferredoxin-NADP reductase. RuBisCO is a bifunctional enzyme that occurs in the stroma of chloroplasts and catalyzes two reactions: photosynthetic carbon dioxide fixation and photorespiratory carbon oxidation. In addition to its enzymatic activities, RuBisCO is thought to be the major nitrogen source providing amino acids for developing organs [28]. In addition, pyruvate orthophosphate dikinase was also induced in the susceptible Mo17 after SCMV inoculation. Pyruvate, orthophosphatedikinase was initially discovered in C4 leaves and is a cardinal carbon-assimilating, stromal enzyme of the C4 photosynthetic pathway. Like several other photosynthetic pathway enzymes, its activity is strictly and reversibly regulated by light. This regulation is conferred by the PPDK regulatory protein (RP), a bifunctional protein kinase/phosphatase that catalyzes the ADP−/Pi-dependent, reversible phosphorylation of an active-site threonine residue [29]. The upregulation of these photosynthesis-related proteins indicates an improved photosynthetic ability in maize leaves after SCMV inoculation, and these results also show that photosynthesis is differentially affected in various hosts during virus infection.

#### Virus coat protein for SCMV infection

Coat protein (CP) is a multifunctional protein that plays a crucial role in the molecular mechanism of the virus infection processes and has an important role also in virus movement [30]. Virus movement in plants is thought to occur through cell-to-cell and systemic movement through the phloem [31], mainly as RNA-movement protein complexes [32]. For potyviruses, CP protein is considered as an important factor in short and long distance movement [33]. We also found the accumulation of CP protein in the SCMV susceptible plant Mo17 (Table 2) but not in the resistant plant Siyi (Table 1), which suggests that CP plays an important role in the process of SCMV infection. Furthermore, it must be noted that the leaves of Siyi plants showed no visible infection symptoms even over a prolonged period of observation, and displayed normal flowering and fruits set.

### Overlap of Differentially Expressed Proteins in Resistant and Susceptible Genotypes

Seventeen proteins showed altered expression in both genotypes. They include glutathione S-transferase, aconitase, histidine triad nucleotide binding protein, nucleoside diphosphate kinase, thioredoxin, abscisic stress ripening protein, cysteine synthase, chaperonin, transketolase, T-complex protein, beta-D-glucosidase precursor, RuBisCo subunit binding-protein beta subunit, RuBisCo large subunit, fructose-bisphosphate aldolase, ferredoxin-NADP reductase, remorin and electron transporter protein. Because of their non-specific expression changes, it is suggested that these proteins are probably not the components specific for gene-for-gene resistance, but might be the components of the basal defense machinery. Among these 17 proteins, 11 proteins showed similar variation trend. For example, electron transporter protein was significantly upregulated after SCMV infection in both the resistant and susceptible genotypes, whereas RuBisCo subunit binding-protein beta subunit was significantly down-regulated after SCMV infection in both genotypes.

Among these proteins, glutathione S-transferase (GST) are a family of multifunctional, dimeric enzymes that catalyse the nucleophilic attack of the tripeptideglutathione on lipophilic compounds with electrophilic centres [34]. Plant GSTs have well described roles in the detoxification and tolerance of crops to herbicides. However, individual GSTs are differentially regulated in response to many forms of biotic and abiotic stress, suggesting diverse functions in endogenous metabolisms. Thus, in a variety of plants, specific GSTs are reported to be induced upon infection, in response to treatment with ozone, hydrogen peroxide, glutathione and biotic elicitors, plant hormones, heavy metals, heat shock, dehydration, wounding and senescence [35]. Nucleoside diphosphate kinase (NDPK) is involved in multiple signaling pathways in mammalian systems, including G-protein signaling. *Arabidopsis* NDPK2, like its mammalian counterparts, is multifunctional despite its initial discovery phytochrome-interacting protein. Resent reports suggest that NDPK2 acts as a GTPase-activating protein for small G proteins in plants and they propose that NDPK2 might be a missing link between the phytochrome mediated light signaling and G protein-mediated signaling [36]. In this study, NDPK was upregulated by SCMV infection, suggesting that NDPK was also involved in plant-virus interaction. In plants, beta-D-glucosidases are involved in various functions, including lignification, regulation of the biological activity of cytokinins [37], control of the biosynthesis of indole-3-acetic acid, and chemical defense against pathogens and herbivores [38]. Beta-D-glucosidases was upregulated by SCMV infection verify its defense roles in maize. However the mechanism of how these proteins contribute to the maize basal defense needs to be further characterized.

### Nineteen Proteins Identified by Proteomics that Previously Unreported in Plant-virus Interactions

Most of the differentially expressed proteins have been previously identified as the virus responsive proteins, although the expression patterns of some proteins were not consistent with previous reports. In addition, there are 19 proteins that have not been reported as virus responsive proteins previously. These proteins include T-complex protein, remorin, beta-D-glucosidase precursor, glutamate dehydrogenase, membrane-associated protein, ZYP1 protein, calcium-dependent protein kinase, bZIP transcription factor ABI5, aconitase, histidine triad nucleotide binding protein, NDPK, T-cytoplasm male sterility restorer factor, Glutamine synthetase, ATP synthase CF1 alpha subunit, exoglucanase1, ubiquitin fusion protein and S-adenosylmethionine synthetase.

Among these proteins, histidine triad nucleotide binding protein belongs to a histidine triad superfamily, which contains a highly conserved His-X-His-X-His-XX motif (X is a hydrophobic amino acid) and plays an important role in many biological processes. Studies of knockout mice have provided evidence that HINT may function as a tumor suppressor, although the mechanism by which HINT suppresses tumor formation is not clear [39], [40]. Our previous study indicated that histidine triad nucleotide binding protein might be involved in the immune response of maize [41]. In this study, histidine triad nucleotide binding protein was upregulated in both resistant and susceptible genotypes, which suggests that it may have important roles in the response to SCMV infection. Remorins are plant-specific proteins that comprise a multigene family in all land plants, including ferns and mosses. They previously annotated as proteins with unknown functions, but are now hypothesized to play important roles during cellular signal transduction processes [42]. Recently, Jarsch and Ott [43] demonstrated that a remorin protein restricts viral movement in tomato leaves and the importance of a symbiosis-specific remorin for bacterial infection of root nodules suggests that these proteins may serve such regulatory functions. It is important to further examine the exact role of these proteins in the plant-virus interactions in the future.

### Analysis of the Consistency between Transcription and Protein Expression Profiles

There are several reports focusing on the SCMV responsive genes or candidate genes putatively associated with resistance to SCMV [44]–[47]. Seven proteins or genes including cytochrome, calcium-dependent protein kinase, germin-like protein, S-adenosylmethionine synthetase, ribosomal protein, thioredoxin and chaperonin were identified both at the transcription and proteomic level. Among them, thioredoxin and chaperonin were identified to be differently expressed genes in the near isogenic lines F7+ (SCMV resistant) and F7 (susceptible) by combined suppression subtractive hybridization and macroarray hybridization [47]. In this study, thioredoxin and chaperonin were respectively downregulated and upregulated in response to SCMV infection (Table 1 and 2). Ribosomal protein was shown to be upregulated following SCMV infection both in unigene-microarray and SSH-macroarray experiments [45]. We also found the upregulation of ribosomal protein at proteomic level (Table 1). Another four proteins calcium-dependent protein kinase, cytochrome, germin-like protein and S-adenosylmethionine synthetase were identified as candidate genes putatively associated with resistance to SCMV by custom-made microarrays [44]. They were all upregulated after SCMV infection at transcription level. At proteomic level, calcium-dependent protein kinase, cytochrome and germin-like protein were upregulated, while S-adenosylmethionine synthetase was downregulated (Table 1 and 2).

The consistency between gene transcription and protein expression levels of some proteins suggests that these proteins may be firstly regulated at the transcriptional level under SCMV infection stress. Inconsistency was also found in several previous reports [21], [48] and some reasons were postulated for the discrepancy between the transcription and protein levels. It was thought that the abundance of a protein integrates post-transcriptional and post-translational processing, which modulates the quantity, temporal expression, localization, and efficiency of the final product in the cell [21].

### Protein-protein Interaction Analysis

**Figure 6:**
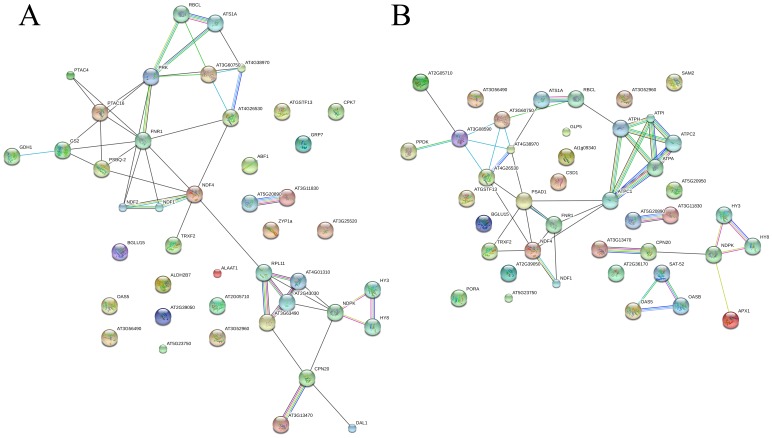
Protein–protein interaction network analyzed by String software. (A) Network analyzed from differentially expressed proteins in sample group Siyi_SCMV_/Siyi_CK._ (B) Network analyzed from differentially expressed proteins in sample group Mo17_SCMV/_Mo17_CK_. Different line colors represent types of evidence for association: green line, neighborhood evidence; red line, fusion evidence; purple line, experimental evidence; light blue line, database evidence; black line, coexpression evidence; blue line, co-occurrence evidence; and yellow line, text-mining evidence.

To determine how SCMV interacts with maize proteins to affect cell function, the proteins identified as being differentially expressed were analyzed by searching the String database and the protein-protein interaction network (Figure 6). Some maize proteins that were significantly altered by SCMV infection were predicted to interact with each other. These protein interactions included (ATS1A-RBCL-PRK-PTAC16-GS2-GDH1; FNR1-NDF2-NDF1-NDF4-TRXF2; HY8-HY3-NDPK-CPN20-AT3G13470; APX1-NDPK-CPN20-AT3G13470 and NDF1-FNR1-PSAD1-AT4G38970-AT3G60750-AT3G08590-PPDK). These proteins have important functions in the defense response, photosynthesis, signal transduction, and energy and metabolism. For example, GS2 (GLUTAMINE SYNTHETASE 2), a light-modulated chloroplast/mitochondrial enzyme, encoded by a nuclear gene and expressed primarily in leaves, is responsible for the reassimilation of the ammonia generated by photorespiration. AT3G52960 (peroxiredoxin), which has thioredoxin peroxidase activity, is upregulated both in resistant and susceptible genotypes (three fold change) by SCMV infection. It plays a role in reduction of hydroperoxides and has been induced during oxidative stress in other systems [49]. The interaction between these proteins may have important roles in SCMV infection.

In addition, the connectivity of proteins in biological networks can also provide insight into their relative importance. Protein or gene “hubs” with a high degree of connectivity (connected to many other proteins or genes) and “bottlenecks”, which reflect key connectors of subnetworks within a network, represent central points for controlling communication within a network and tend to play essential roles in growth, virulence, and targeting by pathogens [50], [51].

### Association of Map Positions of Differentially Expressed Proteins with Genetic Analysis Results

Because SCMV is naturally transmitted by aphids in a non-persistent manner, it is not possible to control SCMV directly with chemical means or through the control of aphid vectors. Therefore, identification of resistant-related genes or proteins and cultivation of resistant maize varieties is the preferred way to control SCMV infections [3]. In previous quantitative trait loci (QTL) experiments, genetic analysis on backcross five (BC5) progeny derived from the cross FAP1360A (resistant) × F7 (susceptible) confirmed that at least two dominant genes, Scmv1 and Scmv2, were required for resistance to SCMV [52]. Xia et al [3] employed composite interval mapping for QTL detection with a linkage map based on 87 restriction fragment length polymorphism and 7 mapped microsatellite markers. Five putative quantitative trait loci (QTL) significantly affecting resistance to SCMV were identified on chromosomes 1 (1.08), 3 (3.04/3.05), 5 (5.01), 6 (6.00), and 10 (10.05) in the joint analyses.

**Figure 7:**
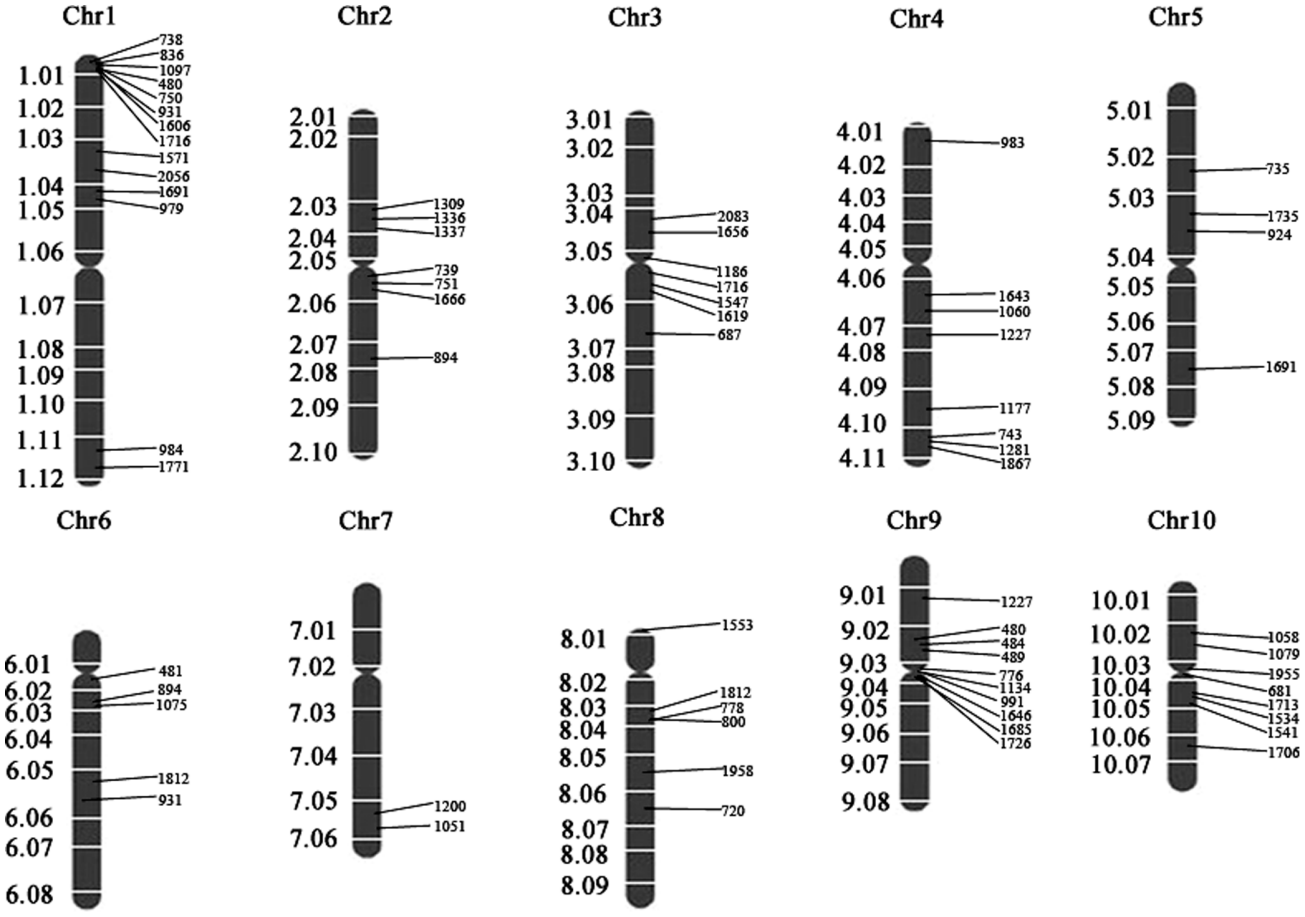
Genetic map positions for genes encoding differentially expressed proteins. Genetic map positions were determined *in silico* using the Maize GDB http://www.maizegdb.org/. Chr, Chromosome.

In this study, genetic map positions for genes encoding the differentially expressed proteins were determined *in silico* using the Maize GDB http://www.maizegdb.org/(Figure 7). Genes encoding 14 of the differentially expressed proteins were located on chromosome 1 in a relatively continuous bin 1.00–1.01, nine were located on chromosome 2, nine on chromosome 4, four on chromosome 5, three on chromosome 7, seven on chromosome 8 and 14 on chromosome 9 and 10, respectively. Furthermore, genes encoding five differentially expressed proteins were assigned to chromosome 6, which carries the *Scmv*1 resistance gene, and another eight genes encoding differentially expressed proteins were assigned to chromosome 3 (in a continuous bin 3.05–3.08), which carries the *Scmv*2 gene.

The percentage of candidate proteins or genes falling into either the Scmv1 (6.00–6.01), Scmv2 (3.04–3.05), or other three QTL regions (1.08, 5.01 and 10.05) is 8% (7 out of 89 mapped differentially expressed proteins), which is not significantly different from the 0-hypothesis tested by the X^2^ test (no clustering of differentially expressed genes). Thus, no evidence of clustering of differentially expressed proteins in the above five QTL regions was found, suggesting that differential expression of proteins due to linkage drag was limited. Moreover, the finding that 7 out of 89 gene locations are in agreement with the five QTL genome locations is not significantly different from expectations based on probability theory. Thus, colocalization of differentially expressed proteins is only a weak indicator for candidacy of being SCMV resistance proteins, which is consistent with the previous transcriptomics analysis [44].

### Accumulation of Phytohormones after virus Inoculation

There is emerging evidence suggesting that a key strategy of plant pathogens is to modify plant hormone levels to promote pathogenicity. Consequently, pathogens have evolved complex repertoires of effector proteins whose functions include modulation of basal phytohormone levels during disease development [53]–[55]. Upon pathogen attack, infected plant cells generate phytohormones signaling molecules to initiate defence mechanisms in surrounding cells to limit pathogen spread. It was reported that five signalling molecules, SA, ABA, JA, ET and ABA, play key roles in mediating disease resistance [56], [57]. Thus, it is important to quantify changes in endogenous concentrations of these phytohormones at different stages of the infection process.

**Figure 8:**
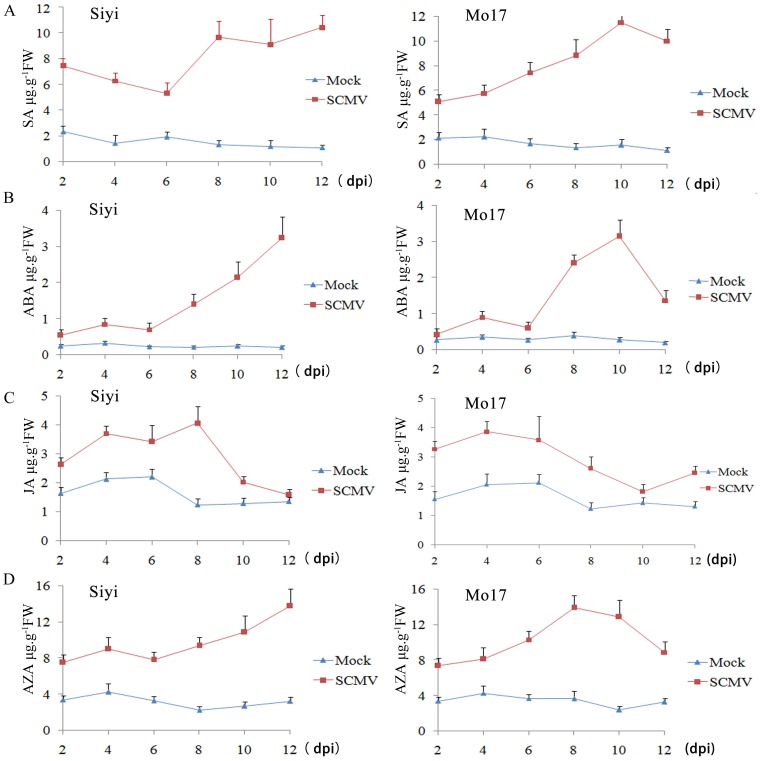
Phytohormone accumulation after inoculation of maize leaves with SCMV. Maize plants (Siyi or Mo17) were mock-inoculated or inoculated with SCMV and leaf samples were collected at 2, 4, 6, 8, 10 and 12 dpi. (A) SA accumulation after inoculation; (B) ABA accumulation after inoculation; (C) JA accumulation after inoculation; (D) AZA accumulation after inoculation. Data are representative of three independent biological experiments. Bars show SE (*n* = 3). FW, fresh weight; dpi, days post inoculation.

We quantified SA, ABA, ET, JA, and AZA after inoculation with SCMV. Inoculation with SCMV led to SA accumulation at 8 dpi in Siyi, while SA accumulated by 4 dpi, peaking at 10 dpi, in Mo17 (Figure 8A). SCMV induced ABA accumulation at 8 dpi in Siyi, while it induced greater accumulation of ABA at 8 dpi in Mo17, peaking at 10 dpi (Figure 8B). JA accumulation was induced at 4 dpi in both Siyi and Mo17, but the concentration decreased after 4 dpi in Mo17 (Figure 8C). After inoculation, similar patterns of AZA accumulation were observed in both genotypes, but the peak in AZA occurred earlier in Mo17 (Figure 8D). No systemic accumulation of ethylene was observed in response to SCMV inoculation (data not shown).

Plants induce long-lasting systemic immunity after local pathogen attack by emitting resistance-priming signals from infection sites. A number of plant molecules have been proposed as mobile factors for this response, but many do not fully satisfy the criteria for timing and action in systemic immunity [58]. AZA has been identified as a pathogen-induced metabolite in *Arabidopsis* vascular sap. It primes plant cells to mount a faster and stronger defense response, including SA production, upon infection [59]. Recently, Zoeller et al [60] suggested that AZA is a general marker for lipid peroxidation, rather than a general immune signal. In this study, both SA and AZA were induced by SCMV inoculation. The changes in the concentrations of SA and AZA showed similar patterns in Mo17, and AZA accumulated to higher levels than SA. In contrast, the changes in the concentrations of SA and AZA showed different patterns in Siyi. These results indicated that the function of AZA in systemic plant immunity might be species-specific.

Our results showed that there were quantitative differences in the accumulation of phytohormones between mock-inoculated and SCMV-inoculated plants. The changes in phytohormone concentrations also differed between the resistant and susceptible maize. In addition, many of the proteins differentially expressed after SCMV infection were also regulated by phytohormones. For example, SOD was downregulated after SCMV infection in Mo17 (Table 2) and it was identified as a SA-responsive protein by liquid chromatography/mass spectrometry [61]. In this study, SCMV also induced SA accumulation at 6 dpi in Mo17 (Figure 8A). The expression level of ferredoxin-NADP reductase was changed in Siyi and Mo17 after SCMV infection (Table 1) and it was also induced by ABA treatment [62]. In addition, the accumulation of ABA was found both in Siyi and Mo17 at 6 dpi, although the greatest accumulation of ABA was at 10 dpi (Figure 8B). Glutamine synthetase was found to be decreased by JA treatment [63]. In our present study, glutamine synthetase was also downregulated after SCMV infection in Siyi (Table 1), which showed a higher level of JA at 6 dpi (Figure 8C). These results suggested that phytohormone-mediated defenses were associated with resistance to SCMV, and many of the differentially expressed proteins may contribute to the maize defense response through the phytohormones-dependent signaling pathways. Further research is required to clarify the details of the defense signaling pathways and the complex interaction between these phytohormones and the differentially expressed proteins.

## Conclusions

The proteomic approach based on protein separation and statistical analysis followed by protein identification has demonstrated outstanding utility to search for potential biomarkers related to the maize response to the SCMV. Proteomic analysis in resistant and susceptible genotypes of maize infected with SCMV revealed 96 protein spots with significant changes. A number of them were identified as energy and metabolism, as well as stress and defense responses, and photosynthesis related proteins. Most of proteins identified were located in chloroplasts, chloroplast membranes or the cytoplasm. In addition, we identified 19 proteins that were not identified as virus responsive protein previously and seven were novel with no known functions. These candidate proteins can be used for reverse genetic experiments to test for their roles in viral pathogenesis, which may provide new insight into the signaling pathways that are modulated by viral infection. It may be of crucial importance in helping and directing programs aimed at improving new crop varieties.

There are overlapping and specific proteomic responses to SCMV infection in resistant and susceptible genotypes. After inoculation, there are 17 proteins altered in both genotypes. Many of these proteins showed different levels of expression or even changes in opposite directions. The functional classification and the subcellular locations of the SCMV responsive proteins in Siyi and Mo17 are similar. However, for the specific functional group, there is a wide difference in the proportion of the upregulated or downregulated proteins. This differential pattern of protein expression between the two different genotypes during SCMV infection indicated that their cellular response to resistance are different at the level detectable by 2-DE. In addition, the changes in concentrations of phytohormones showed quantitative differences between mock-inoculated and SCMV-inoculated plants. The changes in phytohormone concentrations also differed between the resistant and susceptible plants. These results suggested that phytohormone-mediated defenses were associated with resistance to SCMV, although further research is required to elucidate the details of the defense signaling pathways and the functional roles of these phytohormones.

## Supporting Information

Figure S1 SDS-PAGE analysis for the optimal time point for sample collection.
(TIF)

Table S1 List of peptides identified for each single protein by MS/MS analysis.
(DOC)
