# Supplementary figures and images for: Proteomic and Phytohormone Analysis of the Response of Maize (Zea mays L.) Seedlings to Sugarcane Mosaic Virus
Source: PLoS One. 2013 Jul 23;8(7):e70295. doi: 10.1371/journal.pone.0070295 (PMC3720893; doi:10.1371/journal.pone.0070295)

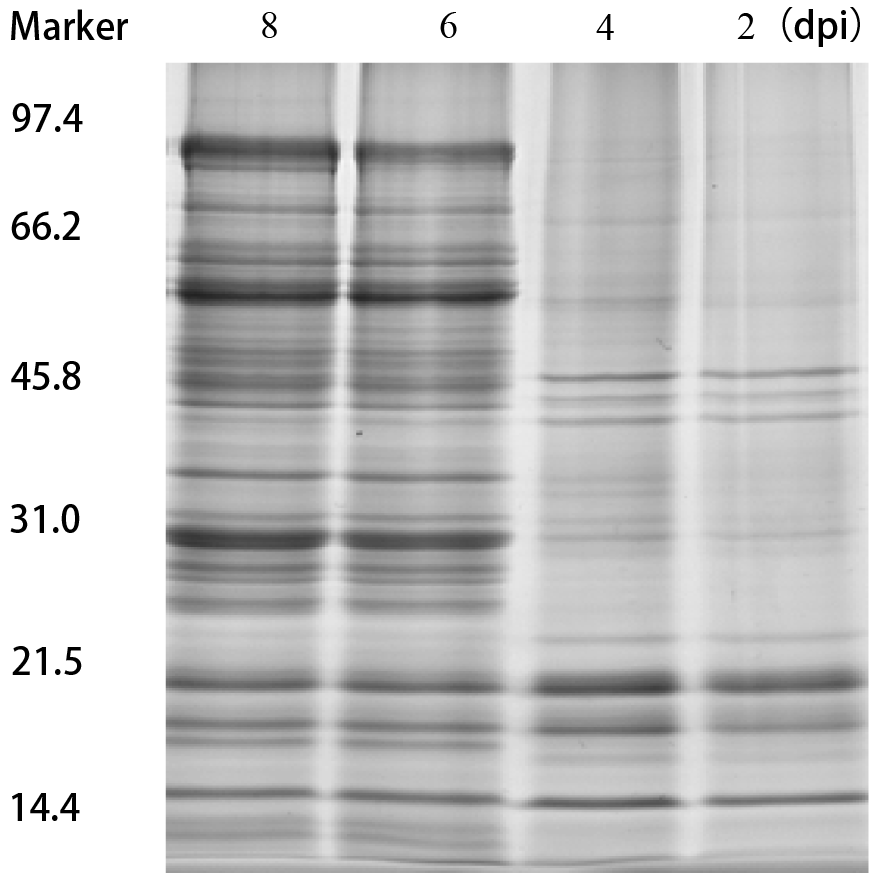

Supplement: Figure S1 — SDS-PAGE analysis for the optimal time point for sample collection. (TIF) [file pone.0070295.s001.tif]
