# Supplementary material for: Proteomic and Phytohormone Analysis of the Response of Maize (Zea mays L.) Seedlings to Sugarcane Mosaic Virus
Source: PLoS One. 2013 Jul 23;8(7):e70295. doi: 10.1371/journal.pone.0070295 (PMC3720893; doi:10.1371/journal.pone.0070295)
Supplement: Table S1 — List of peptides identified for each single protein by MS/MS analysis. (DOC) [file pone.0070295.s002.doc]

Table S1: List of peptides identified for each single protein by MS/MS analysis.

| **Gel Idx/Pos** | **1097** |  |  |  |  |  |  |  |
| --- | --- | --- | --- | --- | --- | --- | --- | --- |
| **Accession No.** | **Protein Name** | **Protein PI** | **Protein MW** | **Protein Score** | **Protein Score C. I. %** | **Total Ion Score** | **Total Ion C. I. %** | **Pep. Count** |
| gi|11467189 | ATP synthase CF1 alpha subunit [Zea mays] | 5.87 | 55729.4 | 856 | 100 | 723 | 100 | 21 |
| **Peptide Information** | | | | | | | | |
| **Calc. Mass** | **Obsrv. Mass** | **± da** | **± ppm** | **Start Seq.** | **End Seq.** | **Sequence** | **Total Ion Score** | **Modification** |
| 917.4799 | 917.5038 | 0.0239 | 26 | 406 | 413 | TSQNQLAR |  |  |
| 920.52 | 920.5378 | 0.0178 | 19 | 457 | 463 | FLDELRK |  |  |
| 947.4792 | 947.5099 | 0.0307 | 32 | 120 | 128 | GEIVASESR |  |  |
| 950.5054 | 950.526 | 0.0206 | 22 | 17 | 23 | IEQYNRK |  |  |
| 985.5789 | 985.6008 | 0.0219 | 22 | 23 | 31 | KVGIENIGR |  |  |
| 1013.5739 | 1013.6096 | 0.0357 | 35 | 32 | 41 | VVQVGDGIAR |  |  |
| 1107.5542 | 1107.5958 | 0.0416 | 38 | 15 | 22 | ERIEQYNR |  |  |
| 1115.5691 | 1115.6136 | 0.0445 | 40 | 492 | 500 | EAIQEQLER |  |  |
| 1115.5691 | 1115.6136 | 0.0445 | 40 | 492 | 500 | EAIQEQLER | 65 |  |
| 1158.6477 | 1158.6454 | -0.0023 | -2 | 2 | 11 | ATLRVDEINK |  |  |
| 1252.726 | 1252.7734 | 0.0474 | 38 | 129 | 140 | LIESPAPGIISR |  |  |
| 1252.726 | 1252.7734 | 0.0474 | 38 | 129 | 140 | LIESPAPGIISR | 88 |  |
| 1266.7528 | 1266.7979 | 0.0451 | 36 | 108 | 119 | VINALAKPIDGR |  |  |
| 1266.7528 | 1266.7979 | 0.0451 | 36 | 108 | 119 | VINALAKPIDGR | 69 |  |
| 1274.6951 | 1274.7288 | 0.0337 | 26 | 177 | 188 | TAVATDTILNQK |  |  |
| 1280.6733 | 1280.7333 | 0.06 | 47 | 481 | 491 | TFTEQAETLLK |  |  |
| 1317.705 | 1317.7284 | 0.0234 | 18 | 256 | 266 | HTLIIYDDLSK |  |  |
| 1416.7845 | 1416.8445 | 0.06 | 42 | 95 | 107 | IAQIPVSEAYLGR |  |  |
| 1416.7845 | 1416.8445 | 0.06 | 42 | 95 | 107 | IAQIPVSEAYLGR | 118 |  |
| 1521.8159 | 1521.8606 | 0.0447 | 29 | 444 | 456 | GYLDSLEIEQVKK |  |  |
| 1577.858 | 1577.8707 | 0.0127 | 8 | 267 | 279 | QAQAYRQMSLLLR |  |  |
| 1852.04 | 1852.0159 | -0.0241 | -13 | 24 | 41 | VGIENIGRVVQVGDGIAR |  |  |
| 2121.0896 | 2121.2178 | 0.1282 | 60 | 42 | 61 | IIGLGEIMSGELVEFAEGTR |  |  |
| 2121.0896 | 2121.2178 | 0.1282 | 60 | 42 | 61 | IIGLGEIMSGELVEFAEGTR | 179 |  |
| 2137.0845 | 2137.1558 | 0.0713 | 33 | 42 | 61 | IIGLGEIMSGELVEFAEGTR |  | Oxidation (M)[8] |
| 2137.0845 | 2137.1558 | 0.0713 | 33 | 42 | 61 | IIGLGEIMSGELVEFAEGTR | 100 | Oxidation (M)[8] |
| 2195.2144 | 2195.2842 | 0.0698 | 32 | 108 | 128 | VINALAKPIDGRGEIVASESR |  |  |
| 2195.2144 | 2195.2842 | 0.0698 | 32 | 108 | 128 | VINALAKPIDGRGEIVASESR | 38 |  |
| 2273.2209 | 2273.3401 | 0.1192 | 52 | 142 | 162 | SVYEPLQTGLIAIDSMIPIGR |  |  |

| **Gel Idx/Pos** | **1060** |  |  |  |  |  |  |  |
| --- | --- | --- | --- | --- | --- | --- | --- | --- |
| **Accession No.** | **Protein Name** | **Protein PI** | **Protein MW** | **Protein Score** | **Protein Score C. I. %** | **Total Ion Score** | **Total Ion C. I. %** | **Pep. Count** |
| gi|195627092 | glutamine synthetase [Zea mays] | 7.64 | 47192.9 | 210 | 100 | 153 | 100 | 10 |
| **Peptide Information** | | | | | | | | |
| **Calc. Mass** | **Obsrv. Mass** | **± da** | **± ppm** | **Start Seq.** | **End Seq.** | **Sequence** | **Total Ion Score** | **Modification** |
| 899.5672 | 899.6208 | 0.0536 | 60 | 324 | 331 | AILNLSLR |  |  |
| 937.4738 | 937.5262 | 0.0524 | 56 | 380 | 387 | GKGYLEDR |  |  |
| 976.5098 | 976.5619 | 0.0521 | 53 | 165 | 173 | AAQIFSDPK |  |  |
| 1539.8265 | 1539.8402 | 0.0137 | 9 | 94 | 107 | TISKPVEDPSELPK |  |  |
| 1597.7354 | 1597.7911 | 0.0557 | 35 | 332 | 345 | HDLHISAYGEGNER |  |  |
| 1732.8402 | 1732.9103 | 0.0701 | 40 | 351 | 366 | HETASIGTFSWGVANR |  |  |
| 1747.9377 | 1748.0062 | 0.0685 | 39 | 74 | 89 | VIAEYIWVGGSGIDIR |  |  |
| 1747.9377 | 1748.0062 | 0.0685 | 39 | 74 | 89 | VIAEYIWVGGSGIDIR | 44 |  |
| 1792.9666 | 1792.9143 | -0.0523 | -29 | 40 | 58 | LAGVQGHGLSTGSTGVVPR |  |  |
| 1797.8575 | 1797.9491 | 0.0916 | 51 | 59 | 73 | LEQLLNMDTTPYTDK |  | Oxidation (M)[7] |
| 2220.1699 | 2220.1682 | -0.0017 | -1 | 174 | 192 | VAAQVPWFGIEQEYTLLQK |  |  |
| 2643.3196 | 2643.3164 | -0.0032 | -1 | 139 | 162 | GGNNVLVICDTYTPQGEPLPTNKR |  | Carbamidomethyl (C)[9] |
| 2643.3196 | 2643.3164 | -0.0032 | -1 | 139 | 162 | GGNNVLVICDTYTPQGE | 109 | Carbamidomethyl (C)[9] |

| **Gel Idx/Pos** | **1075** |  |  |  |  |  |  |  |
| --- | --- | --- | --- | --- | --- | --- | --- | --- |
| **Accession No.** | **Protein Name** | **Protein PI** | **Protein MW** | **Protein Score** | **Protein Score C. I. %** | **Total Ion Score** | **Total Ion C. I. %** | **Pep. Count** |
| gi|326378667 | histidine triad nucleotide binding protein [Zea mays] | 6.35 | 15250.6 | 124 | 100 | 158 | 100 | 4 |
| **Peptide Information** | | | | | | | | |
| **Calc. Mass** | **Obsrv. Mass** | **± da** | **± ppm** | **Start Seq.** | **End Seq.** | **Sequence** | **Total Ion Score** | **Modification** |
| 949.4738 | 949.4692 | -0.0046 | -5 | 85 | 89 | KVVAK |  |  |
| 949.4738 | 949.4692 | -0.0046 | -5 | 27 | 38 | EIPSTVVYEDEK |  |  |
| 957.5727 | 957.5565 | -0.0162 | -17 | 27 | 38 | EIPSTVVYEDEK | 69 |  |
| 1609.8909 | 1609.9384 | 0.0475 | 30 | 61 | 67 | DGLTGLAK |  |  |
| 1609.8909 | 1609.9384 | 0.0475 | 30 | 59 | 72 | VKDGLTGLAKAEER | 78 |  |
| 1737.9858 | 1738.0491 | 0.0633 | 36 | 59 | 72 | VKDGLTGLAKAEER |  |  |

| **Gel Idx/Pos** | **836** |  |  |  |  |  |  |  |
| --- | --- | --- | --- | --- | --- | --- | --- | --- |
| **Accession No.** | **Protein Name** | **Protein PI** | **Protein MW** | **Protein Score** | **Protein Score C. I. %** | **Total Ion Score** | **Total Ion C. I. %** | **Pep. Count** |
| gi|11467189 | ATP synthase CF1 alpha subunit [Zea mays] | 5.87 | 55729.4 | 695 | 100 | 821 | 100 | 25 |
| **Peptide Information** | | | | | | | | |
| **Calc. Mass** | **Obsrv. Mass** | **± da** | **± ppm** | **Start Seq.** | **End Seq.** | **Sequence** | **Total Ion Score** | **Modification** |
| 860.5022 | 860.4832 | -0.019 | -22 | 273 | 279 | QMSLLLR |  |  |
| 876.4971 | 876.4843 | -0.0128 | -15 | 273 | 279 | QMSLLLR |  | Oxidation (M)[2] |
| 917.4799 | 917.4769 | -0.003 | -3 | 406 | 413 | TSQNQLAR |  |  |
| 920.52 | 920.5054 | -0.0146 | -16 | 457 | 463 | FLDELRK |  |  |
| 950.5054 | 950.5004 | -0.005 | -5 | 17 | 23 | IEQYNRK |  |  |
| 985.5789 | 985.5698 | -0.0091 | -9 | 23 | 31 | KVGIENIGR |  |  |
| 1013.5739 | 1013.5781 | 0.0042 | 4 | 32 | 41 | VVQVGDGIAR |  |  |
| 1107.5542 | 1107.569 | 0.0148 | 13 | 15 | 22 | ERIEQYNR |  |  |
| 1229.6848 | 1229.6979 | 0.0131 | 11 | 166 | 176 | ELIIGDRQTGK |  |  |
| 1252.726 | 1252.7401 | 0.0141 | 11 | 129 | 140 | LIESPAPGIISR |  |  |
| 1252.726 | 1252.7401 | 0.0141 | 11 | 129 | 140 | LIESPAPGIISR | 113 |  |
| 1266.7528 | 1266.762 | 0.0092 | 7 | 108 | 119 | VINALAKPIDGR |  |  |
| 1274.6951 | 1274.7064 | 0.0113 | 9 | 177 | 188 | TAVATDTILNQK |  |  |
| 1280.6733 | 1280.683 | 0.0097 | 8 | 481 | 491 | TFTEQAETLLK |  |  |
| 1317.705 | 1317.7074 | 0.0024 | 2 | 256 | 266 | HTLIIYDDLSK |  |  |
| 1408.827 | 1408.851 | 0.024 | 17 | 129 | 141 | LIESPAPGIISRR |  |  |
| 1416.7845 | 1416.8154 | 0.0309 | 22 | 95 | 107 | IAQIPVSEAYLGR |  |  |
| 1416.7845 | 1416.8154 | 0.0309 | 22 | 95 | 107 | IAQIPVSEAYLGR | 116 |  |
| 1521.8159 | 1521.8337 | 0.0178 | 12 | 444 | 456 | GYLDSLEIEQVKK |  |  |
| 1577.858 | 1577.842 | -0.016 | -10 | 267 | 279 | QAQAYRQMSLLLR |  |  |
| 1602.8486 | 1602.8781 | 0.0295 | 18 | 254 | 266 | ERHTLIIYDDLSK |  |  |
| 1948.9611 | 1949.0118 | 0.0507 | 26 | 492 | 507 | EAIQEQLERFSLQEQT |  |  |
| 2121.0896 | 2121.1772 | 0.0876 | 41 | 42 | 61 | IIGLGEIMSGELVEFAEGTR |  |  |
| 2121.0896 | 2121.1772 | 0.0876 | 41 | 42 | 61 | IIGLGEIMSGELVEFAEGTR | 182 |  |
| 2137.0845 | 2137.1294 | 0.0449 | 21 | 42 | 61 | IIGLGEIMSGELVEFAEGTR |  | Oxidation (M)[8] |
| 2137.0845 | 2137.1294 | 0.0449 | 21 | 42 | 61 | IIGLGEIMSGELVEFAEGTR | 92 | Oxidation (M)[8] |
| 2195.2144 | 2195.2703 | 0.0559 | 25 | 108 | 128 | VINALAKPIDGRGEIVASESR |  |  |
| 2195.2144 | 2195.2703 | 0.0559 | 25 | 108 | 128 | VINALAKPIDGRGEIVASESR | 85 |  |
| 2273.2209 | 2273.3079 | 0.087 | 38 | 142 | 162 | SVYEPLQTGLIAIDSMIPIGR |  |  |
| 2289.2158 | 2289.2383 | 0.0225 | 10 | 142 | 162 | SVYEPLQTGLIAIDSMIPIGR |  | Oxidation (M)[16] |
| 2318.1624 | 2318.2446 | 0.0822 | 35 | 423 | 443 | QSQSNPLPVEEQVATIYTGTR |  |  |
| 2318.1624 | 2318.2446 | 0.0822 | 35 | 423 | 443 | QSQSNPLPVEEQVATIYTGTR | 138 |  |
| 2377.2246 | 2377.332 | 0.1074 | 45 | 481 | 500 | TFTEQAETLLKEAIQEQLER |  |  |
| 2377.2246 | 2377.332 | 0.1074 | 45 | 481 | 500 | TFTEQAETLLKEAIQEQLER | 129 |  |

| **Gel Idx/Pos** | **750** |  |  |  |  |  |  |  |
| --- | --- | --- | --- | --- | --- | --- | --- | --- |
| **Accession No.** | **Protein Name** | **Protein PI** | **Protein MW** | **Protein Score** | **Protein Score C. I. %** | **Total Ion Score** | **Total Ion C. I. %** | **Pep. Count** |
| gi|11467200 | ribulose-1,5-bisphosphate carboxylase/oxygenase large subunit [Zea mays] | 6.33 | 53294.6 | 289 | 100 | 836 | 100 | 15 |
| **Peptide Information** | | | | | | | | |
| **Calc. Mass** | **Obsrv. Mass** | **± da** | **± ppm** | **Start Seq.** | **End Seq.** | **Sequence** | **Total Ion Score** | **Modification** |
| 805.4454 | 805.436 | -0.0094 | -12 | 131 | 137 | IPPTYSK |  |  |
| 892.5251 | 892.5317 | 0.0066 | 7 | 1 | 9 | SVGFKAGVK |  |  |
| 914.405 | 914.4689 | 0.0639 | 70 | 297 | 303 | NHGMHFR |  | Oxidation (M)[4] |
| 914.405 | 914.4689 | 0.0639 | 70 | 297 | 303 | NHGMHFR |  | Oxidation (M)[4] |
| 930.4752 | 930.535 | 0.0598 | 64 | 423 | 430 | NEGRDLAR |  |  |
| 930.4752 | 930.535 | 0.0598 | 64 | 423 | 430 | NEGRDLAR | 41 |  |
| 946.4839 | 946.5493 | 0.0654 | 69 | 219 | 227 | AQAETGEIK |  |  |
| 971.4073 | 971.4742 | 0.0669 | 69 | 179 | 185 | ACYECLR |  | Carbamidomethyl (C)[2,5] |
| 971.4073 | 971.4742 | 0.0669 | 69 | 179 | 185 | ACYECLR | 43 | Carbamidomethyl (C)[2,5] |
| 982.488 | 982.5152 | 0.0272 | 28 | 458 | 465 | FEFKPVDT |  |  |
| 985.5789 | 985.6196 | 0.0407 | 41 | 123 | 130 | ALRLEDLR |  |  |
| 985.5789 | 985.6196 | 0.0407 | 41 | 123 | 130 | ALRLEDLR | 12 |  |
| 1021.5312 | 1021.5982 | 0.067 | 66 | 24 | 32 | DTDILAAFR |  |  |
| 1021.5312 | 1021.5982 | 0.067 | 66 | 24 | 32 | DTDILAAFR | 28 |  |
| 1078.6003 | 1078.6606 | 0.0603 | 56 | 169 | 178 | LGLSAKNYGR |  |  |
| 1116.583 | 1116.649 | 0.066 | 59 | 413 | 422 | VALEACVQAR |  | Carbamidomethyl (C)[6] |
| 1116.583 | 1116.649 | 0.066 | 59 | 413 | 422 | VALEACVQAR | 66 | Carbamidomethyl (C)[6] |
| 1257.6797 | 1257.7283 | 0.0486 | 39 | 427 | 437 | DLAREGNEIIK |  |  |
| 1325.6896 | 1325.8002 | 0.1106 | 83 | 297 | 307 | NHGMHFRVLAK |  | Oxidation (M)[4] |
| 1465.7546 | 1465.8328 | 0.0782 | 53 | 138 | 150 | TFQGPPHGIQVER |  |  |
| 1518.741 | 1518.793 | 0.052 | 34 | 207 | 218 | DRFVFCAEAIYK |  | Carbamidomethyl (C)[6] |
| 2169.9871 | 2169.998 | 0.0109 | 5 | 186 | 204 | GGLDFTKDDENVNSQPFMR |  |  |

| **Gel Idx/Pos** | **738** |  |  |  |  |  |  |  |
| --- | --- | --- | --- | --- | --- | --- | --- | --- |
| **Accession No.** | **Protein Name** | **Protein PI** | **Protein MW** | **Protein Score** | **Protein Score C. I. %** | **Total Ion Score** | **Total Ion C. I. %** | **Pep. Count** |
| gi|11467189 | ATP synthase CF1 alpha subunit [Zea mays] | 5.87 | 55729.4 | 913 | 100 | 637 | 100 | 28 |
| **Peptide Information** | | | | | | | | |
| **Calc. Mass** | **Obsrv. Mass** | **± da** | **± ppm** | **Start Seq.** | **End Seq.** | **Sequence** | **Total Ion Score** | **Modification** |
| 815.4621 | 815.4751 | 0.013 | 16 | 166 | 172 | ELIIGDR |  |  |
| 822.4104 | 822.4318 | 0.0214 | 26 | 17 | 22 | IEQYNR |  |  |
| 857.4839 | 857.5032 | 0.0193 | 23 | 24 | 31 | VGIENIGR |  |  |
| 860.5022 | 860.5 | -0.0022 | -3 | 273 | 279 | QMSLLLR |  |  |
| 876.4971 | 876.4957 | -0.0014 | -2 | 273 | 279 | QMSLLLR |  | Oxidation (M)[2] |
| 917.4799 | 917.5083 | 0.0284 | 31 | 406 | 413 | TSQNQLAR |  |  |
| 920.52 | 920.5274 | 0.0074 | 8 | 457 | 463 | FLDELRK |  |  |
| 947.4792 | 947.5168 | 0.0376 | 40 | 120 | 128 | GEIVASESR |  |  |
| 950.5054 | 950.5244 | 0.019 | 20 | 17 | 23 | IEQYNRK |  |  |
| 985.5789 | 985.5869 | 0.008 | 8 | 23 | 31 | KVGIENIGR |  |  |
| 1013.5739 | 1013.5955 | 0.0216 | 21 | 32 | 41 | VVQVGDGIAR |  |  |
| 1013.5739 | 1013.5955 | 0.0216 | 21 | 32 | 41 | VVQVGDGIAR | 69 |  |
| 1107.5542 | 1107.5842 | 0.03 | 27 | 15 | 22 | ERIEQYNR |  |  |
| 1115.5691 | 1115.594 | 0.0249 | 22 | 492 | 500 | EAIQEQLER |  |  |
| 1115.5691 | 1115.594 | 0.0249 | 22 | 492 | 500 | EAIQEQLER | 51 |  |
| 1158.6477 | 1158.6593 | 0.0116 | 10 | 2 | 11 | ATLRVDEINK |  |  |
| 1229.6848 | 1229.7051 | 0.0203 | 17 | 166 | 176 | ELIIGDRQTGK |  |  |
| 1252.726 | 1252.7545 | 0.0285 | 23 | 129 | 140 | LIESPAPGIISR |  |  |
| 1252.726 | 1252.7545 | 0.0285 | 23 | 129 | 140 | LIESPAPGIISR | 94 |  |
| 1266.7528 | 1266.7795 | 0.0267 | 21 | 108 | 119 | VINALAKPIDGR |  |  |
| 1266.7528 | 1266.7795 | 0.0267 | 21 | 108 | 119 | VINALAKPIDGR | 58 |  |
| 1280.6733 | 1280.6896 | 0.0163 | 13 | 481 | 491 | TFTEQAETLLK |  |  |
| 1317.705 | 1317.7633 | 0.0583 | 44 | 256 | 266 | HTLIIYDDLSK |  |  |
| 1408.827 | 1408.8431 | 0.0161 | 11 | 129 | 141 | LIESPAPGIISRR |  |  |
| 1416.7845 | 1416.819 | 0.0345 | 24 | 95 | 107 | IAQIPVSEAYLGR |  |  |
| 1416.7845 | 1416.819 | 0.0345 | 24 | 95 | 107 | IAQIPVSEAYLGR | 111 |  |
| 1521.8159 | 1521.7668 | -0.0491 | -32 | 444 | 456 | GYLDSLEIEQVKK |  |  |
| 1521.8159 | 1521.9054 | 0.0895 | 59 | 444 | 456 | GYLDSLEIEQVKK |  |  |
| 1577.858 | 1577.8555 | -0.0025 | -2 | 267 | 279 | QAQAYRQMSLLLR |  |  |
| 2121.0896 | 2121.1418 | 0.0522 | 25 | 42 | 61 | IIGLGEIMSGELVEFAEGTR |  |  |
| 2137.0845 | 2137.0654 | -0.0191 | -9 | 42 | 61 | IIGLGEIMSGELVEFAEGTR |  | Oxidation (M)[8] |
| 2137.0845 | 2137.0654 | -0.0191 | -9 | 42 | 61 | IIGLGEIMSGELVEFAEGTR | 103 | Oxidation (M)[8] |
| 2195.2144 | 2195.1785 | -0.0359 | -16 | 108 | 128 | VINALAKPIDGRGEIVASESR |  |  |
| 2273.2209 | 2273.2703 | 0.0494 | 22 | 142 | 162 | SVYEPLQTGLIAIDSMIPIGR |  |  |
| 2289.2158 | 2289.1802 | -0.0356 | -16 | 142 | 162 | SVYEPLQTGLIAIDSMIPIGR |  | Oxidation (M)[16] |
| 2318.1624 | 2318.2021 | 0.0397 | 17 | 423 | 443 | QSQSNPLPVEEQVATIYTGTR |  |  |
| 2318.1624 | 2318.2021 | 0.0397 | 17 | 423 | 443 | QSQSNPLPVEEQVATIYTGTR | 152 |  |
| 2377.2246 | 2377.2566 | 0.032 | 13 | 481 | 500 | TFTEQAETLLKEAIQEQLER |  |  |
| 2445.3171 | 2445.2617 | -0.0554 | -23 | 141 | 162 | RSVYEPLQTGLIAIDSMIPIGR |  | Oxidation (M)[17] |

| **Gel Idx/Pos** | **924** |  |  |  |  |  |  |  |
| --- | --- | --- | --- | --- | --- | --- | --- | --- |
| **Accession No.** | **Protein Name** | **Protein PI** | **Protein MW** | **Protein Score** | **Protein Score C. I. %** | **Total Ion Score** | **Total Ion C. I. %** | **Pep. Count** |
| gi|3694807 | alanine aminotransferase [Zea mays] | 6.3 | 53534.3 | 215 | 100 | 223 | 100 | 10 |
| **Peptide Information** | | | | | | | | |
| **Calc. Mass** | **Obsrv. Mass** | **± da** | **± ppm** | **Start Seq.** | **End Seq.** | **Sequence** | **Total Ion Score** | **Modification** |
| 1022.5741 | 1022.5722 | -0.0019 | -2 | 23 | 31 | GEIVIHAQR |  |  |
| 1022.5741 | 1022.5722 | -0.0019 | -2 | 23 | 31 | GEIVIHAQR | 24 |  |
| 1023.5258 | 1023.5598 | 0.034 | 33 | 419 | 427 | APDAFYALR |  |  |
| 1066.5527 | 1066.5643 | 0.0116 | 11 | 87 | 96 | SLFSADAISR |  |  |
| 1089.4783 | 1089.4951 | 0.0168 | 15 | 300 | 308 | GYYGECGKR |  | Carbamidomethyl (C)[6] |
| 1089.4783 | 1089.4951 | 0.0168 | 15 | 300 | 308 | GYYGECGKR | 46 | Carbamidomethyl (C)[6] |
| 1137.6085 | 1137.6077 | -0.0008 | -1 | 14 | 22 | VLKCEYAVR |  | Carbamidomethyl (C)[4] |
| 1151.6207 | 1151.6338 | 0.0131 | 11 | 418 | 427 | KAPDAFYALR |  |  |
| 1151.6207 | 1151.6338 | 0.0131 | 11 | 418 | 427 | KAPDAFYALR | 90 |  |
| 1167.7208 | 1167.7202 | -0.0006 | -1 | 97 | 107 | AKQILATIPGR |  |  |
| 1484.7203 | 1484.7651 | 0.0448 | 30 | 309 | 322 | GGYMEITGFSAPVR |  |  |
| 1484.7203 | 1484.7651 | 0.0448 | 30 | 309 | 322 | GGYMEITGFSAPVR | 64 |  |
| 1500.7152 | 1500.6613 | -0.0539 | -36 | 309 | 322 | GGYMEITGFSAPVR |  | Oxidation (M)[4] |
| 1500.7152 | 1500.7999 | 0.0847 | 56 | 309 | 322 | GGYMEITGFSAPVR |  | Oxidation (M)[4] |
| 1713.9568 | 1713.9144 | -0.0424 | -25 | 1 | 16 | MAASVTVENLNPKVLK |  |  |
| 2184.0676 | 2184.0776 | 0.01 | 5 | 69 | 86 | EVLALCDHPCLLEKEETK |  | Carbamidomethyl (C)[6,10] |

| **Gel Idx/Pos** | **1716** |  |  |  |  |  |  |  |
| --- | --- | --- | --- | --- | --- | --- | --- | --- |
| **Accession No.** | **Protein Name** | **Protein PI** | **Protein MW** | **Protein Score** | **Protein Score C. I. %** | **Total Ion Score** | **Total Ion C. I. %** | **Pep. Count** |
| gi|11467200 | ribulose-1,5-bisphosphate carboxylase/oxygenase large subunit [Zea mays] | 6.33 | 53294.6 | 1,010 | 100 | 643 | 100 | 29 |
| **Peptide Information** | | | | | | | | |
| **Calc. Mass** | **Obsrv. Mass** | **± da** | **± ppm** | **Start Seq.** | **End Seq.** | **Sequence** | **Total Ion Score** | **Modification** |
| 914.405 | 914.4276 | 0.0226 | 25 | 306 | 312 | NHGMHFR |  | Oxidation (M)[4] |
| 930.4752 | 930.5164 | 0.0412 | 44 | 432 | 439 | NEGRDLAR |  |  |
| 946.4839 | 946.5291 | 0.0452 | 48 | 228 | 236 | AQAETGEIK |  |  |
| 971.4073 | 971.4518 | 0.0445 | 46 | 188 | 194 | ACYECLR |  | Carbamidomethyl (C)[2,5] |
| 971.4073 | 971.4518 | 0.0445 | 46 | 188 | 194 | ACYECLR | 47 | Carbamidomethyl (C)[2,5] |
| 983.5197 | 983.4805 | -0.0392 | -40 | 464 | 471 | EIKFDGFK |  |  |
| 985.5789 | 985.6163 | 0.0374 | 38 | 132 | 139 | ALRLEDLR |  |  |
| 985.5789 | 985.6163 | 0.0374 | 38 | 132 | 139 | ALRLEDLR | 35 |  |
| 1021.5312 | 1021.5803 | 0.0491 | 48 | 33 | 41 | DTDILAAFR |  |  |
| 1021.5312 | 1021.5803 | 0.0491 | 48 | 33 | 41 | DTDILAAFR | 73 |  |
| 1037.4899 | 1037.5481 | 0.0582 | 56 | 351 | 358 | DDFIEKDR |  |  |
| 1078.6003 | 1078.6403 | 0.04 | 37 | 178 | 187 | LGLSAKNYGR |  |  |
| 1116.583 | 1116.6328 | 0.0498 | 45 | 422 | 431 | VALEACVQAR |  | Carbamidomethyl (C)[6] |
| 1116.583 | 1116.6328 | 0.0498 | 45 | 422 | 431 | VALEACVQAR | 80 | Carbamidomethyl (C)[6] |
| 1247.6129 | 1247.6572 | 0.0443 | 36 | 218 | 227 | FVFCAEAIYK |  | Carbamidomethyl (C)[4] |
| 1257.6797 | 1257.7463 | 0.0666 | 53 | 436 | 446 | DLAREGNEIIK |  |  |
| 1275.7307 | 1275.787 | 0.0563 | 44 | 340 | 350 | EITLGFVDLLR |  |  |
| 1275.7307 | 1275.787 | 0.0563 | 44 | 340 | 350 | EITLGFVDLLR | 99 |  |
| 1401.7737 | 1401.8282 | 0.0545 | 39 | 135 | 146 | LEDLRIPPAYSK |  |  |
| 1407.6678 | 1407.623 | -0.0448 | -32 | 22 | 32 | LTYYTPEYETK |  |  |
| 1407.6678 | 1407.7579 | 0.0901 | 64 | 22 | 32 | LTYYTPEYETK |  |  |
| 1451.6219 | 1451.7073 | 0.0854 | 59 | 202 | 213 | DDENVNSQPFMR |  |  |
| 1465.7546 | 1465.8286 | 0.074 | 50 | 147 | 159 | TFQGPPHGIQVER |  |  |
| 1465.7546 | 1465.8286 | 0.074 | 50 | 147 | 159 | TFQGPPHGIQVER | 112 |  |
| 1481.7167 | 1481.8228 | 0.1061 | 72 | 320 | 334 | MSGGDHIHSGTVVGK |  |  |
| 1502.8512 | 1502.9038 | 0.0526 | 35 | 165 | 177 | YGRPLLGCTIKPK |  | Carbamidomethyl (C)[8] |
| 1518.741 | 1518.8025 | 0.0615 | 40 | 216 | 227 | DRFVFCAEAIYK |  | Carbamidomethyl (C)[6] |
| 1689.7723 | 1689.8943 | 0.122 | 72 | 188 | 201 | ACYECLRGGLDFTK |  | Carbamidomethyl (C)[2,5] |
| 1708.8766 | 1708.9601 | 0.0835 | 49 | 147 | 161 | TFQGPPHGIQVERDK |  |  |
| 1860.0226 | 1860.0094 | -0.0132 | -7 | 335 | 350 | LEGEREITLGFVDLLR |  |  |
| 1860.0226 | 1860.171 | 0.1484 | 80 | 335 | 350 | LEGEREITLGFVDLLR |  |  |
| 1950.916 | 1950.9956 | 0.0796 | 41 | 237 | 253 | GHYLNATAGTCEEMIKR |  | Carbamidomethyl (C)[11] |
| 1966.911 | 1966.9747 | 0.0637 | 32 | 237 | 253 | GHYLNATAGTCEEMIKR |  | Carbamidomethyl (C)[11], Oxidation (M)[14] |
| 2023.0747 | 2023.0562 | -0.0185 | -9 | 340 | 356 | EITLGFVDLLRDDFIEK |  |  |
| 2023.0747 | 2023.2113 | 0.1366 | 68 | 340 | 356 | EITLGFVDLLRDDFIEK |  |  |
| 2169.9871 | 2170.1018 | 0.1147 | 53 | 195 | 213 | GGLDFTKDDENVNSQPFMR |  |  |
| 2169.9871 | 2170.1018 | 0.1147 | 53 | 195 | 213 | GGLDFTKDDENVNSQPFMR | 130 |  |
| 2185.9819 | 2186.0349 | 0.053 | 24 | 195 | 213 | GGLDFTKDDENVNSQPFMR |  | Oxidation (M)[18] |
| 2222.1716 | 2222.2778 | 0.1062 | 48 | 140 | 159 | IPPAYSKTFQGPPHGIQVER |  |  |
| 2410.1814 | 2410.3167 | 0.1353 | 56 | 22 | 41 | LTYYTPEYETKDTDILAAFR |  |  |
| 2410.1814 | 2410.3167 | 0.1353 | 56 | 22 | 41 | LTYYTPEYETKDTDILAAFR | 148 |  |
| 3870.9033 | 3871.0442 | 0.1409 | 36 | 42 | 79 | VTPQLGVPPEEAGAAVAAESSTGTWTTVWTDGLTSLDR |  |  |

| **Gel Idx/Pos** | **894** |  |  |  |  |  |  |  |
| --- | --- | --- | --- | --- | --- | --- | --- | --- |
| **Accession No.** | **Protein Name** | **Protein PI** | **Protein MW** | **Protein Score** | **Protein Score C. I. %** | **Total Ion Score** | **Total Ion C. I. %** | **Pep. Count** |
| gi|226532728 | electron transporter protein [Zea mays] | 7.57 | 21197.7 | 89 | 99.55 | 65 | 99.897 | 3 |
| **Peptide Information** | | | | | | | | |
| **Calc. Mass** | **Obsrv. Mass** | **± da** | **± ppm** | **Start Seq.** | **End Seq.** | **Sequence** | **Total Ion Score** | **Modification** |
| 827.4046 | 827.4222 | 0.0176 | 21 | 186 | 191 | IHEWDK |  |  |
| 1382.7063 | 1382.712 | 0.0057 | 4 | 80 | 91 | LLPDGTPDVHYR |  |  |
| 1382.7063 | 1382.712 | 0.0057 | 4 | 80 | 91 | LLPDGTPDVHYR | 38 |  |
| 3099.4429 | 3099.5339 | 0.091 | 29 | 50 | 79 | AVEVDAPSAGAPEPEEAEEPSVDFAFVSPR |  |  |
| 3099.4429 | 3099.5339 | 0.091 | 29 | 50 | 79 | AVEVDAPSAGAPEPEEAEEPSVDFAFVSPR | 52 |  |

| **Gel Idx/Pos** | **480** |  |  |  |  |  |  |  |
| --- | --- | --- | --- | --- | --- | --- | --- | --- |
| **Accession No.** | **Protein Name** | **Protein PI** | **Protein MW** | **Protein Score** | **Protein Score C. I. %** | **Total Ion Score** | **Total Ion C. I. %** | **Pep. Count** |
| gi|11467200 | ribulose-1,5-bisphosphate carboxylase/oxygenase large subunit [Zea mays] | 6.33 | 53294.6 | 1,180 | 100 | 836 | 100 | 28 |
| **Peptide Information** | | | | | | | | |
| **Calc. Mass** | **Obsrv. Mass** | **± da** | **± ppm** | **Start Seq.** | **End Seq.** | **Sequence** | **Total Ion Score** | **Modification** |
| 912.472 | 912.4847 | 0.0127 | 14 | 296 | 303 | AMHAVIDR |  |  |
| 914.405 | 914.4255 | 0.0205 | 22 | 306 | 312 | NHGMHFR |  | Oxidation (M)[4] |
| 946.4839 | 946.4996 | 0.0157 | 17 | 228 | 236 | AQAETGEIK |  |  |
| 971.4073 | 971.4283 | 0.021 | 22 | 188 | 194 | ACYECLR |  | Carbamidomethyl (C)[2,5] |
| 971.4073 | 971.4283 | 0.021 | 22 | 188 | 194 | ACYECLR | 34 | Carbamidomethyl (C)[2,5] |
| 983.5197 | 983.4901 | -0.0296 | -30 | 464 | 471 | EIKFDGFK |  |  |
| 985.5789 | 985.592 | 0.0131 | 13 | 132 | 139 | ALRLEDLR |  |  |
| 985.5789 | 985.592 | 0.0131 | 13 | 132 | 139 | ALRLEDLR | 35 |  |
| 1021.5312 | 1021.5588 | 0.0276 | 27 | 33 | 41 | DTDILAAFR |  |  |
| 1021.5312 | 1021.5588 | 0.0276 | 27 | 33 | 41 | DTDILAAFR | 72 |  |
| 1037.4899 | 1037.527 | 0.0371 | 36 | 351 | 358 | DDFIEKDR |  |  |
| 1116.583 | 1116.6162 | 0.0332 | 30 | 422 | 431 | VALEACVQAR |  | Carbamidomethyl (C)[6] |
| 1116.583 | 1116.6162 | 0.0332 | 30 | 422 | 431 | VALEACVQAR | 75 | Carbamidomethyl (C)[6] |
| 1170.5586 | 1170.6744 | 0.1158 | 99 | 304 | 312 | QKNHGMHFR |  | Oxidation (M)[6] |
| 1187.6644 | 1187.7054 | 0.041 | 35 | 286 | 295 | DNGLLLHIHR |  |  |
| 1247.6129 | 1247.6428 | 0.0299 | 24 | 218 | 227 | FVFCAEAIYK |  | Carbamidomethyl (C)[4] |
| 1247.6129 | 1247.6428 | 0.0299 | 24 | 218 | 227 | FVFCAEAIYK | 51 | Carbamidomethyl (C)[4] |
| 1257.6797 | 1257.7312 | 0.0515 | 41 | 436 | 446 | DLAREGNEIIK |  |  |
| 1275.7307 | 1275.7723 | 0.0416 | 33 | 340 | 350 | EITLGFVDLLR |  |  |
| 1275.7307 | 1275.7723 | 0.0416 | 33 | 340 | 350 | EITLGFVDLLR | 99 |  |
| 1401.7737 | 1401.8202 | 0.0465 | 33 | 135 | 146 | LEDLRIPPAYSK |  |  |
| 1407.6678 | 1407.7085 | 0.0407 | 29 | 22 | 32 | LTYYTPEYETK |  |  |
| 1451.6219 | 1451.6956 | 0.0737 | 51 | 202 | 213 | DDENVNSQPFMR |  |  |
| 1465.7546 | 1465.817 | 0.0624 | 43 | 147 | 159 | TFQGPPHGIQVER |  |  |
| 1465.7546 | 1465.817 | 0.0624 | 43 | 147 | 159 | TFQGPPHGIQVER | 115 |  |
| 1481.7167 | 1481.8394 | 0.1227 | 83 | 320 | 334 | MSGGDHIHSGTVVGK |  |  |
| 1502.8512 | 1502.9182 | 0.067 | 45 | 165 | 177 | YGRPLLGCTIKPK |  | Carbamidomethyl (C)[8] |
| 1518.741 | 1518.8207 | 0.0797 | 52 | 216 | 227 | DRFVFCAEAIYK |  | Carbamidomethyl (C)[6] |
| 1534.7358 | 1534.7904 | 0.0546 | 36 | 451 | 463 | WSAELAAACEIWK |  | Carbamidomethyl (C)[9] |
| 1860.0226 | 1860.0095 | -0.0131 | -7 | 335 | 350 | LEGEREITLGFVDLLR |  |  |
| 1860.0226 | 1860.16 | 0.1374 | 74 | 335 | 350 | LEGEREITLGFVDLLR |  |  |
| 1950.916 | 1951.0042 | 0.0882 | 45 | 237 | 253 | GHYLNATAGTCEEMIKR |  | Carbamidomethyl (C)[11] |
| 2023.0747 | 2023.1958 | 0.1211 | 60 | 340 | 356 | EITLGFVDLLRDDFIEK |  |  |
| 2169.9871 | 2170.1069 | 0.1198 | 55 | 195 | 213 | GGLDFTKDDENVNSQPFMR |  |  |
| 2169.9871 | 2170.1069 | 0.1198 | 55 | 195 | 213 | GGLDFTKDDENVNSQPFMR | 131 |  |
| 2185.9819 | 2186.0103 | 0.0284 | 13 | 195 | 213 | GGLDFTKDDENVNSQPFMR |  | Oxidation (M)[18] |
| 2410.1814 | 2410.3411 | 0.1597 | 66 | 22 | 41 | LTYYTPEYETKDTDILAAFR |  |  |
| 2410.1814 | 2410.3411 | 0.1597 | 66 | 22 | 41 | LTYYTPEYETKDTDILAAFR | 148 |  |
| 3870.9033 | 3871.1338 | 0.2305 | 60 | 42 | 79 | VTPQLGVPPEEAGAAVAAESSTGTWTTVWTDGLTSLDR |  |  |
| 3870.9033 | 3871.1338 | 0.2305 | 60 | 42 | 79 | VTPQLGVPPEEAGAAVAAESSTGTWTTVWTDGLTSLDR | 152 |  |

| **Gel Idx/Pos** | **1656** |  |  |  |  |  |  |  |
| --- | --- | --- | --- | --- | --- | --- | --- | --- |
| **Accession No.** | **Protein Name** | **Protein PI** | **Protein MW** | **Protein Score** | **Protein Score C. I. %** | **Total Ion Score** | **Total Ion C. I. %** | **Pep. Count** |
| gi|195607124 | calcium-dependent protein kinase [Zea mays] | 6.28 | 61156.1 | 82 | 96.632 | 136 | 94.828 | 4 |
| **Peptide Information** | | | | | | | | |
| **Calc. Mass** | **Obsrv. Mass** | **± da** | **± ppm** | **Start Seq.** | **End Seq.** | **Sequence** | **Total Ion Score** | **Modification** |
| 2101.1401 | 2101.0813 | -0.0588 | -28 | 353 | 371 | HALRVIAEHLSVEEAADIK |  |  |
| 2105.9526 | 2106.0532 | 0.1006 | 48 | 1 | 21 | MGNCCVTPNGAADAGGGKKPK |  | Carbamidomethyl (C)[4,5], Oxidation (M)[1] |
| 2105.9526 | 2106.0532 | 0.1006 | 48 | 1 | 21 | MGNCCVTPNGAADAGGGKKPK |  | Carbamidomethyl (C)[4,5], Oxidation (M)[1] |
| 2116.0784 | 2116.075 | -0.0034 | -2 | 222 | 239 | FTEIVGSPYYMAPEVLKR |  | Oxidation (M)[11] |
| 2132.0884 | 2132.0859 | -0.0025 | -1 | 30 | 48 | KPNPFSIEYNRSTPASAP |  |  |

| **Gel Idx/Pos** | **1054** |  |  |  |  |  |  |  |
| --- | --- | --- | --- | --- | --- | --- | --- | --- |
| **Accession No.** | **Protein Name** | **Protein PI** | **Protein MW** | **Protein Score** | **Protein Score C. I. %** | **Total Ion Score** | **Total Ion C. I. %** | **Pep. Count** |
| gi|195634659 | fructose-bisphosphate aldolase [Zea mays] | 7.63 | 41923.5 | 806 | 100 | 628 | 100 | 19 |
| **Peptide Information** |  |  |  |  |  |  |  |  |
| **Calc. Mass** | **Obsrv. Mass** | **± da** | **± ppm** | **Start Seq.** | **End Seq.** | **Sequence** | **Total Ion Score** | **Modification** |
| 873.4577 | 873.447 | -0.0107 | -12 | 193 | 200 | EAAWGLAR |  |  |
| 873.4577 | 873.447 | -0.0107 | -12 | 193 | 200 | EAAWGLAR | 64 |  |
| 901.5101 | 901.4606 | -0.0495 | -55 | 359 | 367 | ANSLAQLGK |  |  |
| 947.4979 | 947.4981 | 0.0002 | 0 | 330 | 337 | ALQNTCLK |  | Carbamidomethyl (C)[6] |
| 970.5679 | 970.5674 | -0.0005 | -1 | 348 | 356 | AAQDALLLR |  |  |
| 970.5679 | 970.5674 | -0.0005 | -1 | 348 | 356 | AAQDALLLR | 80 |  |
| 1141.5007 | 1141.5334 | 0.0327 | 29 | 368 | 378 | YTSDGEAAEAK |  |  |
| 1156.5382 | 1156.5477 | 0.0095 | 8 | 163 | 172 | EAAYYQQGAR |  |  |
| 1156.5382 | 1156.5477 | 0.0095 | 8 | 163 | 172 | EAAYYQQGAR | 78 |  |
| 1197.7314 | 1197.7347 | 0.0033 | 3 | 346 | 356 | VKAAQDALLLR |  |  |
| 1291.6893 | 1291.6846 | -0.0047 | -4 | 271 | 282 | ATPEQVAAYTLK |  |  |
| 1387.7175 | 1387.7402 | 0.0227 | 16 | 73 | 85 | LASIGLENTEANR |  |  |
| 1387.7175 | 1387.7402 | 0.0227 | 16 | 73 | 85 | LASIGLENTEANR | 108 |  |
| 1510.8475 | 1510.8336 | -0.0139 | -9 | 178 | 192 | TVVSIPNGPSELAVK |  |  |
| 1543.8187 | 1543.7533 | -0.0654 | -42 | 72 | 85 | RLASIGLENTEANR |  |  |
| 1543.8187 | 1543.9183 | 0.0996 | 65 | 72 | 85 | RLASIGLENTEANR |  |  |
| 1562.8174 | 1562.8511 | 0.0337 | 22 | 269 | 282 | DRATPEQVAAYTLK |  |  |
| 1562.8174 | 1562.8511 | 0.0337 | 22 | 269 | 282 | DRATPEQVAAYTLK | 47 |  |
| 1622.7626 | 1622.6936 | -0.069 | -43 | 58 | 72 | GILAMDESNATCGKR |  | Carbamidomethyl (C)[12] |
| 1622.7626 | 1622.8405 | 0.0779 | 48 | 58 | 72 | GILAMDESNATCGKR |  | Carbamidomethyl (C)[12] |
| 1638.7574 | 1638.6846 | -0.0728 | -44 | 58 | 72 | GILAMDESNATCGKR |  | Carbamidomethyl (C)[12], Oxidation (M)[5] |
| 1832.8372 | 1832.861 | 0.0238 | 13 | 368 | 384 | YTSDGEAAEAKEGMFVK |  |  |
| 1905.9777 | 1906.1462 | 0.1685 | 88 | 73 | 89 | LASIGLENTEANRQAYR |  |  |
| 2365.2874 | 2365.3503 | 0.0629 | 27 | 178 | 200 | TVVSIPNGPSELAVKEAAWGLAR |  |  |
| 2401.1565 | 2401.2778 | 0.1213 | 51 | 140 | 162 | GLVPLAGSNNESWCQGLDGLASR |  | Carbamidomethyl (C)[14] |
| 2401.1565 | 2401.2778 | 0.1213 | 51 | 140 | 162 | GLVPLAGSNNESWCQGLDGLASR | 103 | Carbamidomethyl (C)[14] |
| 2743.3469 | 2743.3606 | 0.0137 | 5 | 137 | 162 | VDKGLVPLAGSNNESWCQGLDGLASR |  | Carbamidomethyl (C)[17] |
| 3047.5684 | 3047.656 | 0.0876 | 29 | 201 | 228 | YAAISQDNGLVPIVEPEILLDGEHGIER |  |  |
| 3047.5684 | 3047.656 | 0.0876 | 29 | 201 | 228 | YAAISQDNGLVPIVEPEILLDGEHGIER | 147 |  |

| **Gel Idx/Pos** | **751** |  |  |  |  |  |  |  |
| --- | --- | --- | --- | --- | --- | --- | --- | --- |
| **Accession No.** | **Protein Name** | **Protein PI** | **Protein MW** | **Protein Score** | **Protein Score C. I. %** | **Total Ion Score** | **Total Ion C. I. %** | **Pep. Count** |
| gi|195625588 | cysteine synthase [Zea mays] | 8.74 | 40833.3 | 187 | 100 | 120 | 100 | 11 |
| **Peptide Information** | | | | | | | | |
| **Calc. Mass** | **Obsrv. Mass** | **± da** | **± ppm** | **Start Seq.** | **End Seq.** | **Sequence** | **Total Ion Score** | **Modification** |
| 849.3883 | 849.3771 | -0.0112 | -13 | 82 | 89 | VTEGCGAR |  | Carbamidomethyl (C)[5] |
| 881.4363 | 881.416 | -0.0203 | -23 | 190 | 196 | ATELYER |  |  |
| 946.4952 | 946.4816 | -0.0136 | -14 | 65 | 73 | DSASQLIGR |  |  |
| 1009.5312 | 1009.5209 | -0.0103 | -10 | 189 | 196 | KATELYER |  |  |
| 1215.6804 | 1215.6901 | 0.0097 | 8 | 63 | 73 | IRDSASQLIGR |  |  |
| 1215.6804 | 1215.6901 | 0.0097 | 8 | 63 | 73 | IRDSASQLIGR | 28 |  |
| 1294.7042 | 1294.7053 | 0.0011 | 1 | 94 | 104 | LEFLQPSFSVK |  |  |
| 1296.7886 | 1296.814 | 0.0254 | 20 | 348 | 359 | LIVTVLPSLGER |  |  |
| 1296.7886 | 1296.814 | 0.0254 | 20 | 348 | 359 | LIVTVLPSLGER | 43 |  |
| 1313.6736 | 1313.7053 | 0.0317 | 24 | 360 | 370 | YLSSALFDELR |  |  |
| 1316.7209 | 1316.6591 | -0.0618 | -47 | 169 | 181 | AFGANLVLTDPAK |  |  |
| 1316.7209 | 1316.7723 | 0.0514 | 39 | 169 | 181 | AFGANLVLTDPAK |  |  |
| 1441.7686 | 1441.8307 | 0.0621 | 43 | 360 | 371 | YLSSALFDELRK |  |  |
| 1441.7686 | 1441.8307 | 0.0621 | 43 | 360 | 371 | YLSSALFDELRK | 50 |  |
| 1630.8218 | 1630.7994 | -0.0224 | -14 | 105 | 118 | DRPAISMLEDAEKR |  |  |
| 1630.8218 | 1630.9662 | 0.1444 | 89 | 105 | 118 | DRPAISMLEDAEKR |  |  |

| **Gel Idx/Pos** | **1691** |  |  |  |  |  |  |  |
| --- | --- | --- | --- | --- | --- | --- | --- | --- |
| **Accession No.** | **Protein Name** | **Protein PI** | **Protein MW** | **Protein Score** | **Protein Score C. I. %** | **Total Ion Score** | **Total Ion C. I. %** | **Pep. Count** |
| gi|195628730 | 2,3-bisphosphoglycerate-independent phosphoglycerate mutase [Zea mays] | 5.29 | 60810.9 | 485 | 100 | 354 | 100 | 19 |
| **Peptide Information** | | | | | | | | |
| **Calc. Mass** | **Obsrv. Mass** | **± da** | **± ppm** | **Start Seq.** | **End Seq.** | **Sequence** | **Total Ion Score** | **Modification** |
| 896.4724 | 896.4702 | -.0022 | -2 | 341 | 348 | TSGEYLVK |  |  |
| 910.5105 | 910.5116 | .0011 | 1 | 163 | 170 | VHILTDGR |  |  |
| 1001.5626 | 1001.571 | .0084 | 8 | 100 | 109 | LVDQALASGK |  |  |
| 1001.5626 | 1001.571 | .0084 | 8 | 100 | 109 | LVDQALASGK | 28 |  |
| 1042.4874 | 1042.4858 | -.0016 | -2 | 353 | 361 | TFACSETVK |  | Carbamidomethyl (C)[4] |
| 1083.6772 | 1083.6699 | -.0073 | -7 | 143 | 151 | LDQLQILLK |  |  |
| 1087.5491 | 1087.5553 | .0062 | 6 | 195 | 206 | GVDAQIASGGGR |  |  |
| 1087.5491 | 1087.5553 | .0062 | 6 | 195 | 206 | GVDAQIASGGGR | 65 |  |
| 1188.626 | 1188.6277 | .0017 | 1 | 331 | 340 | YLVSPPEIDR |  |  |
| 1188.6260 | 1188.628 | .0017 | 1 | 331 | 340 | YLVSPPEIDR | 71 |  |
| 1286.6812 | 1286.691 | .0097 | 8 | 193 | 206 | AKGVDAQIASGGGR |  |  |
| 1304.6157 | 1304.655 | .0395 | 30 | 110 | 120 | IYDGDGFNYIK |  |  |
| 1323.6328 | 1323.663 | .0297 | 22 | 214 | 223 | YENDWDVVKR |  |  |
| 1348.7220 | 1348.726 | .0038 | 3 | 412 | 423 | DALLSGKFDQVR |  |  |
| 1348.7220 | 1348.726 | .0038 | 3 | 412 | 423 | DALLSGKFDQVR | 80 |  |
| 1403.6511 | 1403.705 | .0534 | 38 | 315 | 326 | YAGMLQYDGELK |  | Oxidation (M)[4] |
| 1433.7059 | 1433.734 | .0276 | 19 | 224 | 236 | GWDAQVLGEAPYK |  |  |
| 1475.6438 | 1475.651 | .0074 | 5 | 296 | 307 | ALEYADFDNFDR |  |  |
| 1589.8071 | 1589.785 | -.0219 | -14 | 223 | 236 | RGWDAQVLGEAPYK |  |  |
| 1730.8134 | 1730.819 | .0051 | 3 | 296 | 309 | ALEYADFDNFDRVR |  |  |
| 1730.8134 | 1730.819 | .0051 | 3 | 296 | 309 | ALEYADFDNFDRVR | 54 |  |
| 1856.9211 | 1856.918 | -.0034 | -2 | 315 | 330 | YAGMLQYDGELKLPSR |  | Oxidation (M)[4] |
| 1856.9211 | 1856.918 | -.0034 | -2 | 315 | 330 | YAGMLQYDGELKLPSR | 35 | Oxidation (M)[4] |
| 2066.0806 | 2066.078 | -.0025 | -1 | 331 | 348 | YLVSPPEIDRTSGEYLVK |  |  |
| 2066.0806 | 2066.078 | -.0025 | -1 | 331 | 348 | YLVSPPEIDRTSGEYLVK | 22 |  |
| 2413.1487 | 2413.188 | .0388 | 16 | 424 | 446 | VNLPNGDMVGHTGDIEATVVACK |  | Carbamidomethyl (C)[22], Oxidation (M)[8] |

| **Gel Idx/Pos** | **1186** |  |  |  |  |  |  |  |
| --- | --- | --- | --- | --- | --- | --- | --- | --- |
| **Accession No.** | **Protein Name** | **Protein PI** | **Protein MW** | **Protein Score** | **Protein Score C. I. %** | **Total Ion Score** | **Total Ion C. I. %** | **Pep. Count** |
| gi|195624056 | ferredoxin--NADP reductase, leaf isozyme [Zea mays] | 8.53 | 40976.5 | 111 | 100 | 58 | 99.947 | 10 |
| **Peptide Information** | | | | | | | | |
| **Calc. Mass** | **Obsrv. Mass** | **± da** | **± ppm** | **Start Seq.** | **End Seq.** | **Sequence** | **Total Ion Score** | **Modification** |
| 807.4359 | 807.4337 | -0.0022 | -3 | 284 | 290 | LDFAVSR |  |  |
| 807.4359 | 807.4337 | -0.0022 | -3 | 284 | 290 | LDFAVSR | 13 |  |
| 811.413 | 811.4249 | 0.0119 | 15 | 300 | 305 | MYIQTR |  |  |
| 1236.6736 | 1236.6161 | -0.0575 | -46 | 87 | 96 | YKPKEPYVGR |  |  |
| 1251.6005 | 1251.5712 | -0.0293 | -23 | 360 | 369 | KGEQWNVEVY |  |  |
| 1251.6005 | 1251.5712 | -0.0293 | -23 | 360 | 369 | KGEQWNVEVY | 45 |  |
| 1283.6089 | 1283.5909 | -0.018 | -14 | 347 | 356 | DGIDWMQYKK |  |  |
| 1378.7213 | 1378.6829 | -0.0384 | -28 | 173 | 184 | LVYTNDQGEIVK |  |  |
| 1378.7213 | 1378.6829 | -0.0384 | -28 | 173 | 184 | LVYTNDQGEIVK |  |  |
| 1534.8224 | 1534.7913 | -0.0311 | -20 | 172 | 184 | RLVYTNDQGEIVK |  |  |
| 1534.8224 | 1534.7913 | -0.0311 | -20 | 172 | 184 | RLVYTNDQGEIVK |  |  |
| 1630.7959 | 1630.8209 | 0.025 | 15 | 149 | 164 | LYSIASSALGDFGDSK |  |  |
| 1735.8605 | 1735.829 | -0.0315 | -18 | 331 | 346 | GMEKGIDDIMLDLAAK |  | Oxidation (M)[2] |
| 1739.8381 | 1739.823 | -0.0151 | -9 | 291 | 305 | EQTNAAGEKMYIQTR |  |  |
| 1739.8381 | 1739.823 | -0.0151 | -9 | 291 | 305 | EQTNAAGEKMYIQTR |  |  |

| **Gel Idx/Pos** | **1726** |  |  |  |  |  |  |  |
| --- | --- | --- | --- | --- | --- | --- | --- | --- |
| **Accession No.** | **Protein Name** | **Protein PI** | **Protein MW** | **Protein Score** | **Protein Score C. I. %** | **Total Ion Score** | **Total Ion C. I. %** | **Pep. Count** |
| gi|195619268 | peroxiredoxin-5 [Zea mays] | 7.7399 | 23917.6 | 110 | 100 | 85 | 95 | 5 |
| **Peptide Information** | | | | | | | | |
| **Calc. Mass** | **Obsrv. Mass** | **± da** | **± ppm** | **Start Seq.** | **End Seq.** | **Sequence** | **Total Ion Score** | **Modification** |
| 868.504 | 868.4453 | -0.0587 | -68 | 124 | 131 | HLPGFVAK |  |  |
| 1177.6464 | 1177.6533 | 0.0069 | 6 | 201 | 211 | YALLAEDGVVK |  |  |
| 1177.6464 | 1177.6533 | 0.0069 | 6 | 201 | 211 | YALLAEDGVVK | 30 |  |
| 1333.7474 | 1333.7599 | 0.0125 | 9 | 200 | 211 | RYALLAEDGVVK |  |  |
| 1333.7474 | 1333.7599 | 0.0125 | 9 | 200 | 211 | RYALLAEDGVVK |  |  |
| 1871.9896 | 1872.0991 | 0.1095 | 58 | 180 | 197 | AMGVELDLSDKPVGLGVR |  | Oxidation (M)[2] |
| 1871.9896 | 1872.0991 | 0.1095 | 58 | 180 | 197 | AMGVELDLSDKPVGLGVR | 65 | Oxidation (M)[2] |

| **Gel Idx/Pos** | **489** |  |  |  |  |  |  |  |
| --- | --- | --- | --- | --- | --- | --- | --- | --- |
| **Accession No.** | **Protein Name** | **Protein PI** | **Protein MW** | **Protein Score** | **Protein Score C. I. %** | **Total Ion Score** | **Total Ion C. I. %** | **Pep. Count** |
| gi|28948384 | Chain C, Maize Transketolase In Complex With Tpp | 5.47 | 73346.7 | 772 | 100 | 460 | 100 | 32 |
| **Peptide Information** | | | | | | | | |
| **Calc. Mass** | **Obsrv. Mass** | **± da** | **± ppm** | **Start Seq.** | **End Seq.** | **Sequence** | **Total Ion Score** | **Modification** |
| 802.4417 | 802.4633 | 0.0216 | 27 | 580 | 586 | AADELRK |  |  |
| 914.4617 | 914.489 | 0.0273 | 30 | 326 | 332 | FAEYEKK |  |  |
| 931.5095 | 931.5216 | 0.0121 | 13 | 10 | 18 | AATGELLEK |  |  |
| 967.4731 | 967.5092 | 0.0361 | 37 | 333 | 341 | YADDAATLK |  |  |
| 996.4686 | 996.4901 | 0.0215 | 22 | 63 | 69 | NPYWFNR |  |  |
| 996.4686 | 996.4901 | 0.0215 | 22 | 63 | 69 | NPYWFNR | 48 |  |
| 1005.5615 | 1005.572 | 0.0105 | 10 | 25 | 33 | FLAIDAVEK |  |  |
| 1005.5615 | 1005.572 | 0.0105 | 10 | 25 | 33 | FLAIDAVEK | 39 |  |
| 1012.6262 | 1012.639 | 0.0128 | 13 | 525 | 533 | RPSILALSR |  |  |
| 1012.6262 | 1012.639 | 0.0128 | 13 | 525 | 533 | RPSILALSR | 34 |  |
| 1064.5385 | 1064.5802 | 0.0417 | 39 | 102 | 109 | QFRQWGSR |  |  |
| 1085.6565 | 1085.6443 | -0.0122 | -11 | 247 | 256 | AVTDKPTLIK |  |  |
| 1113.6263 | 1113.6185 | -0.0078 | -7 | 611 | 621 | ESVLPAAVTAR |  |  |
| 1124.4967 | 1124.5203 | 0.0236 | 21 | 230 | 239 | NGNTGYDDIR |  |  |
| 1140.7212 | 1140.7347 | 0.0135 | 12 | 524 | 533 | KRPSILALSR |  |  |
| 1140.7212 | 1140.7347 | 0.0135 | 12 | 524 | 533 | KRPSILALSR | 12 |  |
| 1193.5433 | 1193.5623 | 0.019 | 16 | 359 | 369 | YTPESPGDATR |  |  |
| 1193.5433 | 1193.5623 | 0.019 | 16 | 359 | 369 | YTPESPGDATR | 78 |  |
| 1221.6475 | 1221.6561 | 0.0086 | 7 | 257 | 268 | VTTTIGFGSPNK |  |  |
| 1267.5967 | 1267.6272 | 0.0305 | 24 | 63 | 71 | NPYWFNRDR |  |  |
| 1361.6808 | 1361.7029 | 0.0221 | 16 | 269 | 282 | ANSYSVHGSALGAK |  |  |
| 1389.7373 | 1389.7457 | 0.0084 | 6 | 622 | 634 | ISIEAGSTLGWQK |  |  |
| 1464.7944 | 1464.7976 | 0.0032 | 2 | 660 | 673 | EYGITVESIIAAAK |  |  |
| 1476.8057 | 1476.8113 | 0.0056 | 4 | 536 | 549 | LPHLPGTSIEGVEK |  |  |
| 1486.7842 | 1486.8075 | 0.0233 | 16 | 218 | 229 | FEALGWHTIWVK |  |  |
| 1498.7227 | 1498.7766 | 0.0539 | 36 | 59 | 69 | YNPKNPYWFNR |  |  |
| 1573.6952 | 1573.7306 | 0.0354 | 22 | 403 | 415 | MFGDFQKDTAEER |  |  |
| 1573.6952 | 1573.7306 | 0.0354 | 22 | 403 | 415 | MFGDFQKDTAEER | 101 |  |
| 1589.6901 | 1589.7192 | 0.0291 | 18 | 403 | 415 | MFGDFQKDTAEER |  | Oxidation (M)[1] |
| 1601.8857 | 1601.8718 | -0.0139 | -9 | 10 | 24 | AATGELLEKSVNTIR |  |  |
| 1609.7605 | 1609.7939 | 0.0334 | 21 | 311 | 325 | HTPEGAALEADWNAK |  |  |
| 1732.9592 | 1732.9834 | 0.0242 | 14 | 534 | 549 | QKLPHLPGTSIEGVEK |  |  |
| 1732.9592 | 1732.9834 | 0.0242 | 14 | 534 | 549 | QKLPHLPGTSIEGVEK | 68 |  |
| 1779.964 | 1780.0096 | 0.0456 | 26 | 642 | 659 | AIGIDKFGASAPAGTIYK |  |  |
| 1796.9794 | 1797.0051 | 0.0257 | 14 | 342 | 358 | SIITGELPTGWVDALPK |  |  |
| 1796.9794 | 1797.0051 | 0.0257 | 14 | 342 | 358 | SIITGELPTGWVDALPK | 81 |  |
| 2233.1104 | 2233.2253 | 0.1149 | 51 | 497 | 517 | AMPNILMLRPADGNETAGAYK |  |  |
| 2249.1052 | 2249.1953 | 0.0901 | 40 | 497 | 517 | AMPNILMLRPADGNETAGAYK |  | Oxidation (M)[2] |
| 2265.1003 | 2265.1643 | 0.064 | 28 | 497 | 517 | AMPNILMLRPADGNETAGAYK |  | Oxidation (M)[2,7] |
| 2564.3103 | 2564.4338 | 0.1235 | 48 | 257 | 282 | VTTTIGFGSPNKANSYSVHGSALGAK |  |  |
| 2740.3254 | 2740.4678 | 0.1424 | 52 | 283 | 305 | EVEATRQNLGWPYDTFFVPEDVK |  |  |
| 3038.4988 | 3038.7175 | 0.2187 | 72 | 550 | 579 | GGYTISDNSTGNKPDLIVMGTGSELEIAAK |  |  |
| 3054.4937 | 3054.625 | 0.1313 | 43 | 550 | 579 | GGYTISDNSTGNKPDLIVMGTGSELEIAAK |  | Oxidation (M)[19] |
| 3471.7754 | 3472.022 | 0.2466 | 71 | 110 | 144 | TPGHPENFETPGVEVTTGPLGQGIANAVGLALAEK |  |  |

| **Gel Idx/Pos** | **2083** |  |  |  |  |  |  |  |
| --- | --- | --- | --- | --- | --- | --- | --- | --- |
| **Accession No.** | **Protein Name** | **Protein PI** | **Protein MW** | **Protein Score** | **Protein Score C. I. %** | **Total Ion Score** | **Total Ion C. I. %** | **Pep. Count** |
| gi|195655243 | abscisic stress ripening protein 1 [Zea mays] | 6.81 | 11666.8 | 234 | 100 | 193 | 100 | 5 |
| **Peptide Information** | | | | | | | | |
| **Calc. Mass** | **Obsrv. Mass** | **± da** | **± ppm** | **Start Seq.** | **End Seq.** | **Sequence** | **Total Ion Score** | **Modification** |
| 962.4625 | 962.4984 | 0.0359 | 37 | 33 | 39 | HHKHMEK |  | Oxidation (M)[5] |
| 1218.561 | 1218.5813 | 0.0203 | 17 | 61 | 70 | KDPENEHGHR |  |  |
| 1218.561 | 1218.5813 | 0.0203 | 17 | 61 | 70 | KDPENEHGHR | 53 |  |
| 1257.625 | 1257.6365 | 0.0115 | 9 | 6 | 14 | HHHHLFHHR |  |  |
| 1683.9065 | 1683.9541 | 0.0476 | 28 | 40 | 56 | LGELGAIAAGAYALHEK |  |  |
| 1683.9065 | 1683.9541 | 0.0476 | 28 | 40 | 56 | LGELGAIAAGAYALHEK | 140 |  |
| 2520.2629 | 2520.363 | 0.1001 | 40 | 71 | 94 | VKEEVAAVAAVGSAGFAFHEHHEK |  |  |
| 2520.2629 | 2520.363 | 0.1001 | 40 | 71 | 94 | VKEEVAAVAAVGSAGFAFHEHHEK |  |  |

| **Gel Idx/Pos** | **484** |  |  |  |  |  |  |  |
| --- | --- | --- | --- | --- | --- | --- | --- | --- |
| **Accession No.** | **Protein Name** | **Protein PI** | **Protein MW** | **Protein Score** | **Protein Score C. I. %** | **Total Ion Score** | **Total Ion C. I. %** | **Pep. Count** |
| gi|28948384 | Chain C, Maize Transketolase In Complex With Tpp | 5.47 | 73346.7 | 713 | 100 | 451 | 100 | 29 |
| **Peptide Information** | | | | | | | | |
| **Calc. Mass** | **Obsrv. Mass** | **± da** | **± ppm** | **Start Seq.** | **End Seq.** | **Sequence** | **Total Ion Score** | **Modification** |
| 802.4417 | 802.4785 | 0.0368 | 46 | 580 | 586 | AADELRK |  |  |
| 872.3971 | 872.4606 | 0.0635 | 73 | 403 | 409 | MFGDFQK |  |  |
| 914.4617 | 914.4923 | 0.0306 | 33 | 326 | 332 | FAEYEKK |  |  |
| 931.5095 | 931.549 | 0.0395 | 42 | 10 | 18 | AATGELLEK |  |  |
| 967.4731 | 967.5074 | 0.0343 | 35 | 333 | 341 | YADDAATLK |  |  |
| 996.4686 | 996.5043 | 0.0357 | 36 | 63 | 69 | NPYWFNR |  |  |
| 996.4686 | 996.5043 | 0.0357 | 36 | 63 | 69 | NPYWFNR | 41 |  |
| 1005.5615 | 1005.5917 | 0.0302 | 30 | 25 | 33 | FLAIDAVEK |  |  |
| 1005.5615 | 1005.5917 | 0.0302 | 30 | 25 | 33 | FLAIDAVEK | 48 |  |
| 1012.6262 | 1012.6555 | 0.0293 | 29 | 525 | 533 | RPSILALSR |  |  |
| 1012.6262 | 1012.6555 | 0.0293 | 29 | 525 | 533 | RPSILALSR | 35 |  |
| 1085.6565 | 1085.6635 | 0.007 | 6 | 247 | 256 | AVTDKPTLIK |  |  |
| 1124.4967 | 1124.5399 | 0.0432 | 38 | 230 | 239 | NGNTGYDDIR |  |  |
| 1140.7212 | 1140.7445 | 0.0233 | 20 | 524 | 533 | KRPSILALSR |  |  |
| 1193.5433 | 1193.5885 | 0.0452 | 38 | 359 | 369 | YTPESPGDATR |  |  |
| 1193.5433 | 1193.5885 | 0.0452 | 38 | 359 | 369 | YTPESPGDATR | 63 |  |
| 1221.6475 | 1221.6821 | 0.0346 | 28 | 257 | 268 | VTTTIGFGSPNK |  |  |
| 1267.5967 | 1267.6483 | 0.0516 | 41 | 63 | 71 | NPYWFNRDR |  |  |
| 1361.6808 | 1361.7273 | 0.0465 | 34 | 269 | 282 | ANSYSVHGSALGAK |  |  |
| 1389.7373 | 1389.7766 | 0.0393 | 28 | 622 | 634 | ISIEAGSTLGWQK |  |  |
| 1464.7944 | 1464.8196 | 0.0252 | 17 | 660 | 673 | EYGITVESIIAAAK |  |  |
| 1476.8057 | 1476.8309 | 0.0252 | 17 | 536 | 549 | LPHLPGTSIEGVEK |  |  |
| 1486.7842 | 1486.8035 | 0.0193 | 13 | 218 | 229 | FEALGWHTIWVK |  |  |
| 1498.7227 | 1498.7985 | 0.0758 | 51 | 59 | 69 | YNPKNPYWFNR |  |  |
| 1573.6952 | 1573.7524 | 0.0572 | 36 | 403 | 415 | MFGDFQKDTAEER |  |  |
| 1573.6952 | 1573.7524 | 0.0572 | 36 | 403 | 415 | MFGDFQKDTAEER | 104 |  |
| 1589.6901 | 1589.7439 | 0.0538 | 34 | 403 | 415 | MFGDFQKDTAEER |  | Oxidation (M)[1] |
| 1589.6901 | 1589.7439 | 0.0538 | 34 | 403 | 415 | MFGDFQKDTAEER | 78 | Oxidation (M)[1] |
| 1601.8857 | 1601.8915 | 0.0058 | 4 | 10 | 24 | AATGELLEKSVNTIR |  |  |
| 1609.7605 | 1609.812 | 0.0515 | 32 | 311 | 325 | HTPEGAALEADWNAK |  |  |
| 1609.7605 | 1609.812 | 0.0515 | 32 | 311 | 325 | HTPEGAALEADWNAK | 100 |  |
| 1732.9592 | 1733.0103 | 0.0511 | 29 | 534 | 549 | QKLPHLPGTSIEGVEK |  |  |
| 1732.9592 | 1733.0103 | 0.0511 | 29 | 534 | 549 | QKLPHLPGTSIEGVEK | 59 |  |
| 1779.964 | 1780.0125 | 0.0485 | 27 | 642 | 659 | AIGIDKFGASAPAGTIYK |  |  |
| 1796.9794 | 1797.0287 | 0.0493 | 27 | 342 | 358 | SIITGELPTGWVDALPK |  |  |
| 2233.1104 | 2233.1973 | 0.0869 | 39 | 497 | 517 | AMPNILMLRPADGNETAGAYK |  |  |
| 2249.1052 | 2249.1521 | 0.0469 | 21 | 497 | 517 | AMPNILMLRPADGNETAGAYK |  | Oxidation (M)[2] |
| 2265.1003 | 2265.1262 | 0.0259 | 11 | 497 | 517 | AMPNILMLRPADGNETAGAYK |  | Oxidation (M)[2,7] |
| 2740.3254 | 2740.4624 | 0.137 | 50 | 283 | 305 | EVEATRQNLGWPYDTFFVPEDVK |  |  |
| 3038.4988 | 3038.6423 | 0.1435 | 47 | 550 | 579 | GGYTISDNSTGNKPDLIVMGTGSELEIAAK |  |  |
| 3054.4937 | 3054.6233 | 0.1296 | 42 | 550 | 579 | GGYTISDNSTGNKPDLIVMGTGSELEIAAK |  | Oxidation (M)[19] |

| **Gel Idx/Pos** | **1336** |  |  |  |  |  |  |  |
| --- | --- | --- | --- | --- | --- | --- | --- | --- |
| **Accession No.** | **Protein Name** | **Protein PI** | **Protein MW** | **Protein Score** | **Protein Score C. I. %** | **Total Ion Score** | **Total Ion C. I. %** | **Pep. Count** |
| gi|195628632 | remorin [Zea mays] | 5.74 | 21875.4 | 179 | 100 | 132 | 100 | 8 |
| **Peptide Information** | | | | | | | | |
| **Calc. Mass** | **Obsrv. Mass** | **± da** | **± ppm** | **Start Seq.** | **End Seq.** | **Sequence** | **Total Ion Score** | **Modification** |
| 804.4097 | 804.3653 | -0.0444 | -55 | 2 | 8 | AEEEAKK |  |  |
| 807.3552 | 807.3758 | 0.0206 | 26 | 1 | 7 | MAEEEAK |  |  |
| 820.4345 | 820.387 | -0.0475 | -58 | 163 | 169 | AMVEAKR |  | Oxidation (M)[2] |
| 838.4304 | 838.3969 | -0.0335 | -40 | 140 | 146 | KAEYAEK |  |  |
| 905.3999 | 905.4108 | 0.0109 | 12 | 94 | 100 | AWEENEK |  |  |
| 1372.6927 | 1372.6785 | -0.0142 | -10 | 70 | 82 | QGGSNDRDLALAR |  |  |
| 1372.6927 | 1372.6785 | -0.0142 | -10 | 70 | 82 | QGGSNDRDLALAR | 43 |  |
| 1419.7478 | 1419.7333 | -0.0145 | -10 | 56 | 69 | VADEPAPEKPAPAK |  |  |
| 1419.7478 | 1419.7333 | -0.0145 | -10 | 56 | 69 | VADEPAPEKPAPAK | 36 |  |
| 2144.196 | 2144.1069 | -0.0891 | -42 | 49 | 69 | ALAIVEKVADEPAPEKPAPAK |  |  |
| 2144.196 | 2144.1069 | -0.0891 | -42 | 49 | 69 | ALAIVEKVADEPAPEKPAPAK | 53 |  |
| 2144.196 | 2144.3032 | 0.1072 | 50 | 49 | 69 | ALAIVEKVADEPAPEKPAPAK |  |  |

| **Gel Idx/Pos** | **681** |  |  |  |  |  |  |  |
| --- | --- | --- | --- | --- | --- | --- | --- | --- |
| **Accession No.** | **Protein Name** | **Protein PI** | **Protein MW** | **Protein Score** | **Protein Score C. I. %** | **Total Ion Score** | **Total Ion C. I. %** | **Pep. Count** |
| gi|343227637 | beta-D-glucosidase precursor [Zea mays] | 6.75 | 63463.4 | 585 | 100 | 444 | 100 | 19 |
| **Peptide Information** | | | | | | | | |
| **Calc. Mass** | **Obsrv. Mass** | **± da** | **± ppm** | **Start Seq.** | **End Seq.** | **Sequence** | **Total Ion Score** | **Modification** |
| 807.4359 | 807.4657 | 0.0298 | 37 | 474 | 479 | LDYIQR |  |  |
| 807.4359 | 807.4657 | 0.0298 | 37 | 474 | 479 | LDYIQR | 30 |  |
| 956.4836 | 956.4343 | -0.0493 | -52 | 516 | 523 | YGIAYVDR |  |  |
| 963.537 | 963.563 | 0.026 | 27 | 473 | 479 | RLDYIQR |  |  |
| 972.4244 | 972.4561 | 0.0317 | 33 | 339 | 346 | GDYPFSMR |  |  |
| 988.4193 | 988.4448 | 0.0255 | 26 | 339 | 346 | GDYPFSMR |  |  |
| 988.4193 | 988.4448 | 0.0255 | 26 | 339 | 346 | GDYPFSMR | 10 | Oxidation (M)[7] |
| 1004.3812 | 1004.4262 | 0.045 | 45 | 132 | 139 | EMGMDAYR |  | Oxidation (M)[7] |
| 1094.5664 | 1094.5844 | 0.018 | 16 | 299 | 308 | IGLAFDVMGR |  | Oxidation (M)[2,4] |
| 1094.5664 | 1094.5844 | 0.018 | 16 | 299 | 308 | IGLAFDVMGR | 25 | Oxidation (M)[8] |
| 1138.5891 | 1138.6138 | 0.0247 | 22 | 351 | 359 | ERLPFFSDK |  | Oxidation (M)[8] |
| 1138.5891 | 1138.6138 | 0.0247 | 22 | 351 | 359 | ERLPFFSDK | 37 |  |
| 1211.642 | 1211.6578 | 0.0158 | 13 | 207 | 216 | IVNDYKNFAK |  |  |
| 1328.6304 | 1328.6521 | 0.0217 | 16 | 217 | 227 | VCFDNFGDKVK |  |  |
| 1358.6443 | 1358.6707 | 0.0264 | 19 | 129 | 139 | LLKEMGMDAYR |  | Carbamidomethyl (C)[2] |
| 1366.7002 | 1366.7059 | 0.0057 | 4 | 353 | 363 | LPFFSDKQQEK |  | Oxidation (M)[5,7] |
| 1454.7162 | 1454.7358 | 0.0196 | 13 | 309 | 321 | VPYGTSFLDEQAK |  |  |
| 1634.8094 | 1634.8352 | 0.0258 | 16 | 459 | 472 | EKPLPMEAALNDYK |  |  |
| 1728.8076 | 1728.8224 | 0.0148 | 9 | 151 | 166 | GTVEGGINQDGIDYYK |  | Oxidation (M)[6] |
| 1739.8599 | 1739.9043 | 0.0444 | 26 | 309 | 323 | VPYGTSFLDEQAKER |  |  |
| 1739.8599 | 1739.9043 | 0.0444 | 26 | 309 | 323 | VPYGTSFLDEQAKER | 121 |  |
| 1790.9105 | 1790.9425 | 0.032 | 18 | 459 | 473 | EKPLPMEAALNDYKR |  |  |
| 1808.9501 | 1808.999 | 0.0489 | 27 | 47 | 63 | VGNENGVQLLSPSEIPR |  | Oxidation (M)[6] |
| 1808.9501 | 1808.999 | 0.0489 | 27 | 47 | 63 | VGNENGVQLLSPSEIPR | 123 |  |
| 1884.9087 | 1884.9615 | 0.0528 | 28 | 151 | 167 | GTVEGGINQDGIDYYKR |  |  |
| 1884.9087 | 1884.9615 | 0.0528 | 28 | 151 | 167 | GTVEGGINQDGIDYYKR | 100 |  |
| 1965.0513 | 1965.0959 | 0.0446 | 23 | 47 | 64 | VGNENGVQLLSPSEIPRR |  |  |

| **Gel Idx/Pos** | **743** |  |  |  |  |  |  |  |
| --- | --- | --- | --- | --- | --- | --- | --- | --- |
| **Accession No.** | **Protein Name** | **Protein PI** | **Protein MW** | **Protein Score** | **Protein Score C. I. %** | **Total Ion Score** | **Total Ion C. I. %** | **Pep. Count** |
| gi|359497202 | PREDICTED: T-complex protein 1 [Vitis vinifera] | 6.2 | 61032.7 | 86 | 100 | 68.369 | 0 | 9 |
| **Peptide Information** | | | | | | | | |
| **Calc. Mass** | **Obsrv. Mass** | **± da** | **± ppm** | **Start Seq.** | **End Seq.** | **Sequence** | **Total Ion Score** | **Modification** |
| 861.4611 | 861.5008 | 0.0397 | 46 | 202 | 209 | KVPGGTMR |  | Oxidation (M)[7] |
| 981.5588 | 981.5421 | -0.0167 | -17 | 425 | 433 | QHARTIAGK |  |  |
| 981.5588 | 981.5421 | -0.0167 | -17 | 425 | 433 | QHARTIAGK |  |  |
| 989.4898 | 989.53 | 0.0402 | 41 | 317 | 324 | VTEEDLQR |  |  |
| 989.4898 | 989.53 | 0.0402 | 41 | 317 | 324 | VTEEDLQR |  |  |
| 1076.6462 | 1076.6072 | -0.039 | -36 | 71 | 80 | LLDIVHPAAK |  |  |
| 1154.6351 | 1154.6672 | 0.0321 | 28 | 391 | 400 | SLHDAIMIVR |  |  |
| 1170.63 | 1170.6392 | 0.0092 | 8 | 391 | 400 | SLHDAIMIVR | 71 | Oxidation (M)[7] |
| 1196.6311 | 1196.6332 | 0.0021 | 2 | 210 | 220 | DSFLVNGVAFK |  |  |
| 1321.6019 | 1321.6436 | 0.0417 | 32 | 379 | 390 | GGADQFIEEAER |  |  |
| 1321.6019 | 1321.6436 | 0.0417 | 32 | 379 | 390 | GGADQFIEEAER | 24 | 24 |
| 1402.6638 | 1402.7015 | 0.0377 | 27 | 222 | 233 | TFSYAGFEQQPK |  |  |
| 1968.0009 | 1968.1 | 0.0991 | 50 | 361 | 378 | FNIFSGCPSGQTATIVLR |  | Carbamidomethyl (C)[7] |
| 1968.0009 | 1968.1 | 0.0991 | 50 | 361 | 378 | FNIFSGCPSGQTATIVLR |  | Carbamidomethyl (C)[7] |

| **Gel Idx/Pos** | **1958** |  |  |  |  |  |  |  |
| --- | --- | --- | --- | --- | --- | --- | --- | --- |
| **Accession No.** | **Protein Name** | **Protein PI** | **Protein MW** | **Protein Score** | **Protein Score C. I. %** | **Total Ion Score** | **Total Ion C. I. %** | **Pep. Count** |
| gi|195640294 | thioredoxin F-type [Zea mays] | 8.88 | 21062.1 | 78 | 99.84 | 63 | 99.985 | 3 |
| **Peptide Information** | | | | | | | | |
| **Calc. Mass** | **Obsrv. Mass** | **± da** | **± ppm** | **Start Seq.** | **End Seq.** | **Sequence** | **Total Ion Score** | **Modification** |
| 947.556 | 947.5222 | -0.0338 | -36 | 139 | 146 | NLDVVFLK |  |  |
| 1428.7581 | 1428.6979 | -0.0602 | -42 | 185 | 197 | IDELAEAIEIANK |  |  |
| 1556.853 | 1556.8086 | -0.0444 | -29 | 185 | 198 | IDELAEAIEIANKK |  |  |
| 1556.853 | 1556.8086 | -0.0444 | -29 | 185 | 198 | IDELAEAIEIANKK | 63 |  |

| **Gel Idx/Pos** | **800** |  |  |  |  |  |  |  |
| --- | --- | --- | --- | --- | --- | --- | --- | --- |
| **Accession No.** | **Protein Name** | **Protein PI** | **Protein MW** | **Protein Score** | **Protein Score C. I. %** | **Total Ion Score** | **Total Ion C. I. %** | **Pep. Count** |
| gi|195636170 | hypothetical protein [Zea mays] | 6.53 | 42290.4 | 584 | 100 | 397 | 100 | 21 |
| **Peptide Information** | | | | | | | | |
| **Calc. Mass** | **Obsrv. Mass** | **± da** | **± ppm** | **Start Seq.** | **End Seq.** | **Sequence** | **Total Ion Score** | **Modification** |
| 855.4359 | 855.4671 | 0.0312 | 36 | 356 | 363 | GVNSFGFK |  |  |
| 856.5363 | 856.5422 | 0.0059 | 7 | 217 | 223 | ILNNKVR |  |  |
| 856.5363 | 856.5422 | 0.0059 | 7 | 217 | 223 | ILNNKVR | 7 |  |
| 885.4101 | 885.4417 | 0.0316 | 36 | 349 | 355 | YDDGIFR |  |  |
| 885.4101 | 885.4417 | 0.0316 | 36 | 349 | 355 | YDDGIFR | 26 |  |
| 942.5618 | 942.5795 | 0.0177 | 19 | 196 | 203 | IEEAVLLR |  |  |
| 978.5255 | 978.5512 | 0.0257 | 26 | 142 | 149 | DTLFEVVR |  |  |
| 978.5255 | 978.5512 | 0.0257 | 26 | 142 | 149 | DTLFEVVR | 41 |  |
| 1070.6569 | 1070.6598 | 0.0029 | 3 | 196 | 204 | IEEAVLLRK |  |  |
| 1107.5834 | 1107.5795 | -0.0039 | -4 | 205 | 213 | IYGVDYHLK |  |  |
| 1213.6212 | 1213.6255 | 0.0043 | 4 | 346 | 355 | VTKYDDGIFR |  |  |
| 1235.6783 | 1235.6179 | -0.0604 | -49 | 204 | 213 | KIYGVDYHLK |  |  |
| 1250.6012 | 1250.6178 | 0.0166 | 13 | 150 | 161 | VGDDADAVYGLR |  |  |
| 1250.6012 | 1250.6178 | 0.0166 | 13 | 150 | 161 | VGDDADAVYGLR | 98 |  |
| 1332.6577 | 1332.6707 | 0.013 | 10 | 178 | 189 | ESCLNASIPSVR |  | Carbamidomethyl (C)[3] |
| 1421.7423 | 1421.719 | -0.0233 | -16 | 205 | 216 | IYGVDYHLKDAK |  |  |
| 1460.7526 | 1460.7765 | 0.0239 | 16 | 178 | 190 | ESCLNASIPSVRK |  | Carbamidomethyl (C)[3] |
| 1494.77 | 1494.8231 | 0.0531 | 36 | 246 | 258 | YVVSNGKIWDASR |  |  |
| 1511.8792 | 1511.873 | -0.0062 | -4 | 191 | 203 | EAQLKIEEAVLLR |  |  |
| 1511.8792 | 1511.873 | -0.0062 | -4 | 191 | 203 | EAQLKIEEAVLLR | 60 |  |
| 1524.7152 | 1524.7205 | 0.0053 | 3 | 98 | 109 | FTTYNYMIDGKR |  | Oxidation (M)[7] |
| 1556.7704 | 1556.769 | -0.0014 | -1 | 356 | 369 | GVNSFGFKSEVTER |  |  |
| 1556.7704 | 1556.769 | -0.0014 | -1 | 356 | 369 | GVNSFGFKSEVTER | 109 |  |
| 1721.8282 | 1721.8396 | 0.0114 | 7 | 349 | 363 | YDDGIFRGVNSFGFK |  |  |
| 2024.0117 | 2024.0013 | -0.0104 | -5 | 172 | 189 | LTYEGKESCLNASIPSVR |  | Carbamidomethyl (C)[9] |
| 2359.0911 | 2359.1128 | 0.0217 | 9 | 77 | 97 | MVEGYPYLQFASDDIGDPSVR |  |  |
| 2359.0911 | 2359.1128 | 0.0217 | 9 | 77 | 97 | MVEGYPYLQFASDDIGDPSVR | 56 |  |
| 2375.0859 | 2375.0972 | 0.0113 | 5 | 77 | 97 | MVEGYPYLQFASDDIGDPSVR |  | Oxidation (M)[1] |
| 2800.3711 | 2800.3281 | -0.043 | -15 | 320 | 344 | ILATQGTCQVPFSYTQEDILTTGEK |  | Carbamidomethyl (C)[8] |

| **Gel Idx/Pos** | **1541** |  |  |  |  |  |  |  |
| --- | --- | --- | --- | --- | --- | --- | --- | --- |
| **Accession No.** | **Protein Name** | **Protein PI** | **Protein MW** | **Protein Score** | **Protein Score C. I. %** | **Total Ion Score** | **Total Ion C. I. %** | **Pep. Count** |
| gi|195612242 | cytochrome P450 CYP714B3 [Zea mays] | 8.96 | 59241.8 | 111 | 99.172 | 80 | 90 | 11 |
| **Peptide Information** | | | | | | | | |
| **Calc. Mass** | **Obsrv. Mass** | **± da** | **± ppm** | **Start Seq.** | **End Seq.** | **Sequence** | **Total Ion Score** | **Modification** |
| 800.376 | 800.4259 | 0.0499 | 62 | 265 | 270 | FFPSMR |  | Oxidation (M)[5] |
| 821.4186 | 821.4531 | 0.0345 | 42 | 516 | 522 | VQSVCTK |  | Carbamidomethyl (C)[5] |
| 911.4733 | 911.4573 | -0.016 | -18 | 273 | 279 | QAWELHK |  |  |
| 929.5162 | 929.4431 | -0.0731 | -79 | 64 | 73 | EAAAAAKAAR |  |  |
| 977.5197 | 977.47 | -0.0497 | -51 | 516 | 523 | VQSVCTKR |  | Carbamidomethyl (C)[5] |
| 1057.4731 | 1057.5652 | 0.0921 | 87 | 358 | 365 | EEVHEVCR |  | Carbamidomethyl (C)[7] |
| 1080.525 | 1080.5728 | 0.0478 | 44 | 1 | 10 | MEVAMAMAVK |  |  |
| 1080.5837 | 1080.5728 | -0.0109 | -10 | 390 | 399 | LYPAGAFVSR |  |  |
| 1128.5098 | 1128.5265 | 0.0167 | 15 | 1 | 10 | MEVAMAMAVK |  | Oxidation (M)[1,5,7] |
| 1192.6613 | 1192.5804 | -0.0809 | -68 | 170 | 179 | IIAPEFFLDK |  |  |
| 1192.6613 | 1192.5804 | -0.0809 | -68 | 170 | 179 | IIAPEFFLDK | 55 |  |
| 1317.7195 | 1317.7715 | 0.052 | 39 | 379 | 389 | NLTMVIQETLR |  |  |
| 1317.7195 | 1317.7715 | 0.052 | 39 | 379 | 389 | NLTMVIQETLR | 20 |  |
| 1419.8247 | 1419.7462 | -0.0785 | -55 | 170 | 181 | IIAPEFFLDKVK |  |  |
| 1419.8247 | 1419.7462 | -0.0785 | -55 | 170 | 181 | IIAPEFFLDKVK | 36 |  |

| **Gel Idx/Pos** | **1227** |  |  |  |  |  |  |  |
| --- | --- | --- | --- | --- | --- | --- | --- | --- |
| **Accession No.** | **Protein Name** | **Protein PI** | **Protein MW** | **Protein Score** | **Protein Score C. I. %** | **Total Ion Score** | **Total Ion C. I. %** | **Pep. Count** |
| gi|195643284 | chaperonin [Zea mays] | 7.74 | 25750.7 | 140 | 100 | 110 | 100 | 4 |
| **Peptide Information** | | | | | | | | |
| **Calc. Mass** | **Obsrv. Mass** | **± da** | **± ppm** | **Start Seq.** | **End Seq.** | **Sequence** | **Total Ion Score** | **Modification** |
| 801.3777 | 801.3204 | -0.0573 | -72 | 220 | 226 | YAGSEFK |  |  |
| 1004.4829 | 1004.4242 | -0.0587 | -58 | 152 | 159 | DMKPLNDR |  | Oxidation (M)[2] |
| 1135.6106 | 1135.5983 | -0.0123 | -11 | 227 | 237 | GADGTAYIVLR |  |  |
| 1135.6106 | 1135.5983 | -0.0123 | -11 | 227 | 237 | GADGTAYIVLR | 46 |  |
| 1917.9706 | 1918.0106 | 0.04 | 21 | 220 | 237 | YAGSEFKGADGTAYIVLR |  |  |
| 1917.9706 | 1918.0106 | 0.04 | 21 | 220 | 237 | YAGSEFKGADGTAYIVLR | 64 |  |
| 2563.4204 | 2563.4814 | 0.061 | 24 | 73 | 99 | TVGGILLPSTAQTKPQGG |  |  |

| **Gel Idx/Pos** | **1058** |  |  |  |  |  |  |  |
| --- | --- | --- | --- | --- | --- | --- | --- | --- |
| **Accession No.** | **Protein Name** | **Protein PI** | **Protein MW** | **Protein Score** | **Protein Score C. I. %** | **Total Ion Score** | **Total Ion C. I. %** | **Pep. Count** |
| gi|195634659 | fructose-bisphosphate aldolase [Zea mays] | 7.63 | 41923.5 | 864 | 100 | 716 | 100 | 17 |
| **Peptide Information** | | | | | | | | |
| **Calc. Mass** | **Obsrv. Mass** | **± da** | **± ppm** | **Start Seq.** | **End Seq.** | **Sequence** | **Total Ion Score** | **Modification** |
| 873.4577 | 873.475 | 0.0173 | 20 | 193 | 200 | EAAWGLAR |  |  |
| 873.4577 | 873.475 | 0.0173 | 20 | 193 | 200 | EAAWGLAR | 65 |  |
| 901.5101 | 901.4832 | -0.0269 | -30 | 359 | 367 | ANSLAQLGK |  |  |
| 947.4979 | 947.511 | 0.0131 | 14 | 330 | 337 | ALQNTCLK |  | Carbamidomethyl (C)[6] |
| 970.5679 | 970.5774 | 0.0095 | 10 | 348 | 356 | AAQDALLLR |  |  |
| 970.5679 | 970.5774 | 0.0095 | 10 | 348 | 356 | AAQDALLLR | 76 |  |
| 1115.5845 | 1115.5916 | 0.0071 | 6 | 338 | 347 | TWGGQPDKVK |  |  |
| 1141.5007 | 1141.533 | 0.0323 | 28 | 368 | 378 | YTSDGEAAEAK |  |  |
| 1156.5382 | 1156.55 | 0.0118 | 10 | 163 | 172 | EAAYYQQGAR |  |  |
| 1156.5382 | 1156.55 | 0.0118 | 10 | 163 | 172 | EAAYYQQGAR | 82 |  |
| 1291.6893 | 1291.6742 | -0.0151 | -12 | 271 | 282 | ATPEQVAAYTLK |  |  |
| 1291.6893 | 1291.6742 | -0.0151 | -12 | 271 | 282 | ATPEQVAAYTLK | 54 |  |
| 1387.7175 | 1387.7168 | -0.0007 | -1 | 73 | 85 | LASIGLENTEANR |  |  |
| 1387.7175 | 1387.7168 | -0.0007 | -1 | 73 | 85 | LASIGLENTEANR | 116 |  |
| 1510.8475 | 1510.8247 | -0.0228 | -15 | 178 | 192 | TVVSIPNGPSELAVK |  |  |
| 1543.8187 | 1543.814 | -0.0047 | -3 | 72 | 85 | RLASIGLENTEANR |  |  |
| 1562.8174 | 1562.8113 | -0.0061 | -4 | 269 | 282 | DRATPEQVAAYTLK |  |  |
| 1562.8174 | 1562.8113 | -0.0061 | -4 | 269 | 282 | DRATPEQVAAYTLK | 34 |  |
| 1622.7626 | 1622.7561 | -0.0065 | -4 | 58 | 72 | GILAMDESNATCGKR |  | Carbamidomethyl (C)[12] |
| 1638.7574 | 1638.7316 | -0.0258 | -16 | 58 | 72 | GILAMDESNATCGKR |  | Carbamidomethyl (C)[12], Oxidation (M)[5] |
| 1848.832 | 1848.8112 | -0.0208 | -11 | 368 | 384 | YTSDGEAAEAKEGMFVK |  | Oxidation (M)[14] |
| 2401.1565 | 2401.1997 | 0.0432 | 18 | 140 | 162 | GLVPLAGSNNESWCQGLDGLASR |  | Carbamidomethyl (C)[14] |
| 2401.1565 | 2401.1997 | 0.0432 | 18 | 140 | 162 | GLVPLAGSNNESWCQGLDGLASR | 137 | Carbamidomethyl (C)[14] |
| 2743.3469 | 2743.4126 | 0.0657 | 24 | 137 | 162 | VDKGLVPLAGSNNESWCQGLDGLASR |  | Carbamidomethyl (C)[17] |
| 3047.5684 | 3047.6914 | 0.123 | 40 | 201 | 228 | YAAISQDNGLVPIVEPEILLDGEHGIER |  |  |
| 3047.5684 | 3047.6914 | 0.123 | 40 | 201 | 228 | YAAISQDNGLVPIVEPEILLDGEHGIER | 152 |  |

| **Gel Idx/Pos** | **776** |  |  |  |  |  |  |  |
| --- | --- | --- | --- | --- | --- | --- | --- | --- |
| **Accession No.** | **Protein Name** | **Protein PI** | **Protein MW** | **Protein Score** | **Protein Score C. I. %** | **Total Ion Score** | **Total Ion C. I. %** | **Pep. Count** |
| gi|195633817 | oxygen-evolving enhancer protein 3-1 [Zea mays] | 9.77 | 23104.5 | 234 | 100 | 109 | 100 | 7 |
| **Peptide Information** | | | | | | | | |
| **Calc. Mass** | **Obsrv. Mass** | **± da** | **± ppm** | **Start Seq.** | **End Seq.** | **Sequence** | **Total Ion Score** | **Modification** |
| 1128.6047 | 1128.6049 | 0.0002 | 0 | 150 | 158 | ASYLRYDLK |  |  |
| 1132.5997 | 1132.5946 | -0.0051 | -5 | 97 | 105 | DFDLPLKER |  |  |
| 1132.5997 | 1132.5946 | -0.0051 | -5 | 97 | 105 | DFDLPLKER | 40 |  |
| 1261.6324 | 1261.6335 | 0.0011 | 1 | 138 | 147 | AWPYVQNDLR |  |  |
| 1261.6324 | 1261.6335 | 0.0011 | 1 | 138 | 147 | AWPYVQNDLR | 16 |  |
| 1445.7272 | 1445.726 | -0.0012 | -1 | 179 | 191 | LFSTIDDLDHAAK |  |  |
| 1445.7272 | 1445.726 | -0.0012 | -1 | 179 | 191 | LFSTIDDLDHAAK |  |  |
| 1543.8268 | 1543.8317 | 0.0049 | 3 | 106 | 119 | FYLQPLPPAEAAAR |  |  |
| 1543.8268 | 1543.8317 | 0.0049 | 3 | 106 | 119 | FYLQPLPPAEAAAR |  |  |
| 1582.8475 | 1582.7803 | -0.0672 | -42 | 201 | 215 | YFAATKDALGDVLAK |  |  |
| 2002.0353 | 2002.0341 | -0.0012 | -1 | 76 | 96 | IGAPPPPSGGLPGTLNSDQAR |  |  |
| 2002.0353 | 2002.0341 | -0.0012 | -1 | 76 | 96 | IGAPPPPSGGLPGTLNSD |  |  |

| **Gel Idx/Pos** | **720** |  |  |  |  |  |  |  |
| --- | --- | --- | --- | --- | --- | --- | --- | --- |
| **Accession No.** | **Protein Name** | **Protein PI** | **Protein MW** | **Protein Score** | **Protein Score C. I. %** | **Total Ion Score** | **Total Ion C. I. %** | **Pep. Count** |
| gi|226502949 | 60S ribosomal protein L5-1 [Zea mays] | 9.33 | 34461.8 | 96 | 99.972 | 49 | 94.828 | 10 |
| **Peptide Information** | | | | | | | | |
| **Calc. Mass** | **Obsrv. Mass** | **± da** | **± ppm** | **Start Seq.** | **End Seq.** | **Sequence** | **Total Ion Score** | **Modification** |
| 809.4152 | 809.4111 | -0.0041 | -5 | 272 | 277 | LTYEQR |  |  |
| 809.4152 | 809.4111 | -0.0041 | -5 | 272 | 277 | LTYEQR |  |  |
| 810.4104 | 810.3989 | -0.0115 | -14 | 26 | 32 | AGKTDYR |  |  |
| 937.5101 | 937.5068 | -0.0033 | -4 | 272 | 278 | LTYEQRK |  |  |
| 937.5101 | 937.5068 | -0.0033 | -4 | 271 | 277 | KLTYEQR |  |  |
| 969.6091 | 969.6022 | -0.0069 | -7 | 144 | 152 | ALLDVGLIR |  |  |
| 969.6091 | 969.6022 | -0.0069 | -7 | 144 | 152 | ALLDVGLIR | 49 |  |
| 972.5472 | 972.4948 | -0.0524 | -54 | 37 | 44 | LINQDKNK |  |  |
| 1095.5906 | 1095.6001 | 0.0095 | 9 | 189 | 197 | QLDADIHRK |  |  |
| 1264.7008 | 1264.6827 | -0.0181 | -14 | 153 | 164 | TTTGNRVFGALK |  |  |
| 1370.6145 | 1370.6676 | 0.0531 | 39 | 230 | 241 | GIEADDMEALYK |  | Oxidation (M)[7] |
| 1564.8079 | 1564.8363 | 0.0284 | 18 | 165 | 179 | GALDGGLDIPHSEKR |  |  |

| **Gel Idx/Pos** | **1534** |  |  |  |  |  |  |  |
| --- | --- | --- | --- | --- | --- | --- | --- | --- |
| **Accession No.** | **Protein Name** | **Protein PI** | **Protein MW** | **Protein Score** | **Protein Score C. I. %** | **Total Ion Score** | **Total Ion C. I. %** | **Pep. Count** |
| gi|304651309 | ZYP1 protein [Zea mays] | 5.92 | 101020.6 | 92 | 93.927 | 70 | 100 | 19 |
| **Peptide Information** | | | | | | | | |
| **Calc. Mass** | **Obsrv. Mass** | **± da** | **± ppm** | **Start Seq.** | **End Seq.** | **Sequence** | **Total Ion Score** | **Modification** |
| 806.3825 | 806.3815 | -0.001 | -1 | 710 | 716 | VMGESQR |  |  |
| 825.4689 | 825.4265 | -0.0424 | -51 | 2 | 8 | QKHSGLR |  |  |
| 832.4774 | 832.4515 | -0.0259 | -31 | 210 | 216 | EKDSLIK |  |  |
| 850.4166 | 850.4249 | 0.0083 | 10 | 734 | 740 | RSDPYGR |  |  |
| 873.4788 | 873.4131 | -0.0657 | -75 | 415 | 422 | LASELQGR |  |  |
| 880.441 | 880.4528 | 0.0118 | 13 | 169 | 175 | LEYAEQK |  |  |
| 901.5717 | 901.4977 | -0.074 | -82 | 48 | 55 | ITAEKLVK |  |  |
| 918.4527 | 918.4297 | -0.023 | -25 | 717 | 724 | VDQEVNSK |  |  |
| 939.475 | 939.4234 | -0.0516 | -55 | 332 | 339 | MLMARSSK |  | Oxidation (M)[1] |
| 948.4819 | 948.4644 | -0.0175 | -18 | 265 | 272 | NSLELMNK |  |  |
| 955.4699 | 955.4492 | -0.0207 | -22 | 332 | 339 | MLMARSSK |  | Oxidation (M)[1,3] |
| 964.4767 | 964.4586 | -0.0181 | -19 | 265 | 272 | NSLELMNK | 20 | Oxidation (M)[6] |
| 1043.519 | 1043.5383 | 0.0193 | 18 | 62 | 70 | TDLEMAHVK |  |  |
| 1080.4626 | 1080.5582 | 0.0956 | 88 | 188 | 195 | QEKEEMDR |  | Oxidation (M)[6] |
| 1080.4626 | 1080.5582 | 0.0956 | 88 | 188 | 195 | QEKEEMDR | 71 | Oxidation (M)[6] |
| 1227.5536 | 1227.6036 | 0.05 | 41 | 305 | 315 | EHASMSSHVSR |  |  |
| 1362.6934 | 1362.7125 | 0.0191 | 14 | 686 | 696 | MQEELELQKSK |  |  |
| 1371.759 | 1371.6682 | -0.0908 | -66 | 73 | 84 | RATEQINIIEGK |  |  |
| 1419.7334 | 1419.7354 | 0.002 | 1 | 236 | 246 | LQCMEQELKLK |  | Carbamidomethyl (C)[3] |
| 1419.7334 | 1419.7354 | 0.002 | 1 | 236 | 246 | LQCMEQELKLK |  | Carbamidomethyl (C)[3] |
| 1445.6981 | 1445.7338 | 0.0357 | 25 | 287 | 298 | DLNELFSSFMVK |  | Oxidation (M)[10] |
| 1496.7592 | 1496.6787 | -0.0805 | -54 | 199 | 211 | GQLYSNDTTIKEK |  |  |
| 2022.9478 | 2022.9866 | 0.0388 | 19 | 316 | 331 | LISSFERFYEMAQEEK |  | Oxidation (M)[11] |

| **Gel Idx/Pos** | **1200** |  |  |  |  |  |  |  |
| --- | --- | --- | --- | --- | --- | --- | --- | --- |
| **Accession No.** | **Protein Name** | **Protein PI** | **Protein MW** | **Protein Score** | **Protein Score C. I. %** | **Total Ion Score** | **Total Ion C. I. %** | **Pep. Count** |
| gi|194707256 | unknown [Zea mays] | 5.96 | 33565.8 | 462 | 100 | 388 | 100 | 10 |
| **Peptide Information** | | | | | | | | |
| **Calc. Mass** | **Obsrv. Mass** | **± da** | **± ppm** | **Start Seq.** | **End Seq.** | **Sequence** | **Total Ion Score** | **Modification** |
| 946.5139 | 946.5154 | 0.0015 | 2 | 175 | 182 | DGKVCLVR |  | Carbamidomethyl (C)[5] |
| 946.5139 | 946.5154 | 0.0015 | 2 | 175 | 182 | DGKVCLVR | 46 | Carbamidomethyl (C)[5] |
| 1196.5542 | 1196.5414 | -0.0128 | -11 | 164 | 174 | AGEDQYSLASR |  |  |
| 1196.5542 | 1196.5414 | -0.0128 | -11 | 164 | 174 | AGEDQYSLASR | 71 |  |
| 1496.6976 | 1496.6742 | -0.0234 | -16 | 164 | 177 | AGEDQYSLASRDGK |  |  |
| 1564.7167 | 1564.7206 | 0.0039 | 2 | 2 | 13 | EFPHHHHHHGHR |  |  |
| 1673.9109 | 1673.7657 | -0.1452 | -87 | 202 | 216 | VKDEEGYPAIVLVNK |  |  |
| 1673.9109 | 1673.9209 | 0.01 | 6 | 202 | 216 | VKDEEGYPAIVLVNK |  |  |
| 1673.9109 | 1673.9209 | 0.01 | 6 | 202 | 216 | VKDEEGYPAIVLVNK | 65 |  |
| 1695.7571 | 1695.8625 | 0.1054 | 62 | 1 | 13 | MEFPHHHHHHGHR |  |  |
| 1760.8273 | 1760.8219 | -0.0054 | -3 | 160 | 174 | IYCKAGEDQYSLASR |  | Carbamidomethyl (C)[3] |
| 1760.8273 | 1760.8219 | -0.0054 | -3 | 160 | 174 | IYCKAGEDQYSLASR | 50 | Carbamidomethyl (C)[3] |
| 1884.8723 | 1884.8674 | -0.0049 | -3 | 237 | 252 | HNPDSLDESVLWTESR |  |  |
| 1884.8723 | 1884.8674 | -0.0049 | -3 | 237 | 252 | HNPDSLDESVLWTESR | 106 |  |
| 2048.9131 | 2048.9387 | 0.0256 | 12 | 285 | 301 | DGTALVLWEWCEGDNQR |  | Carbamidomethyl (C)[11] |
| 2165.0452 | 2165.0757 | 0.0305 | 14 | 23 | 43 | GPPPPVYGGYGQPPPPDPYGR |  |  |
| 2165.0452 | 2165.0757 | 0.0305 | 14 | 23 | 43 | GPPPPVYGGYGQPPPPDPYGR | 50 |  |

| **Gel Idx/Pos** | **1657** |  |  |  |  |  |  |  |
| --- | --- | --- | --- | --- | --- | --- | --- | --- |
| **Accession No.** | **Protein Name** | **Protein PI** | **Protein MW** | **Protein Score** | **Protein Score C. I. %** | **Total Ion Score** | **Total Ion C. I. %** | **Pep. Count** |
| gi|126116602 | coat protein [Sugarcane mosaic virus] | 7.18 | 31744.8 | 95 | 100 | 26.0 | 63 | 6 |
| **Peptide Information** | | | | | | | | |
| **Calc. Mass** | **Obsrv. Mass** | **± da** | **± ppm** | **Start Seq.** | **End Seq.** | **Sequence** | **Total Ion Score** | **Modification** |
| 809.4192 | 809.3765 | -0.0427 | -53 | 141 | 146 | WYEAIK |  |  |
| 889.4196 | 889.4383 | 0.0187 | 21 | 253 | 259 | CIQDPTR |  | Carbamidomethyl (C)[1] |
| 905.3968 | 905.4727 | 0.0759 | 84 | 262 | 268 | QAHMQMK |  | Oxidation (M)[4,6] |
| 1009.4697 | 1009.496 | 0.0263 | 26 | 133 | 140 | ASREEFDR |  |  |
| 1106.5325 | 1106.5754 | 0.0429 | 39 | 79 | 89 | DKDVDAGTTGK |  |  |
| 1401.7584 | 1401.8009 | 0.0425 | 30 | 81 | 94 | DVDAGTTGKITVPK |  |  |
| 1401.7584 | 1401.8009 | 0.0425 | 30 | 81 | 94 | DVDAGTTGKITVPK | 81 |  |

| **Gel Idx/Pos** | **480** |  |  |  |  |  |  |  |
| --- | --- | --- | --- | --- | --- | --- | --- | --- |
| **Accession No.** | **Protein Name** | **Protein PI** | **Protein MW** | **Protein Score** | **Protein Score C. I. %** | **Total Ion Score** | **Total Ion C. I. %** | **Pep. Count** |
| gi|75140229 | RecName: Full=Transketolase, chloroplastic; Short=TK | 5.47 | 73346.7 | 889 | 100 | 586 | 100 | 31 |
| **Peptide Information** | | | | | | | | |
| **Calc. Mass** | **Obsrv. Mass** | **± da** | **± ppm** | **Start Seq.** | **End Seq.** | **Sequence** | **Total Ion Score** | **Modification** |
| 802.4417 | 802.437 | -0.0047 | -6 | 580 | 586 | AADELRK |  |  |
| 872.3971 | 872.4185 | 0.0214 | 25 | 403 | 409 | MFGDFQK |  |  |
| 914.4617 | 914.4694 | 0.0077 | 8 | 326 | 332 | FAEYEKK |  |  |
| 931.5095 | 931.4986 | -0.0109 | -12 | 10 | 18 | AATGELLEK |  |  |
| 967.4731 | 967.4836 | 0.0105 | 11 | 333 | 341 | YADDAATLK |  |  |
| 996.4686 | 996.4827 | 0.0141 | 14 | 63 | 69 | NPYWFNR |  |  |
| 996.4686 | 996.4827 | 0.0141 | 14 | 63 | 69 | NPYWFNR | 46 |  |
| 1005.5615 | 1005.559 | -0.0025 | -2 | 25 | 33 | FLAIDAVEK |  |  |
| 1005.5615 | 1005.559 | -0.0025 | -2 | 25 | 33 | FLAIDAVEK | 31 |  |
| 1012.6262 | 1012.6204 | -0.0058 | -6 | 525 | 533 | RPSILALSR |  |  |
| 1085.6565 | 1085.6392 | -0.0173 | -16 | 247 | 256 | AVTDKPTLIK |  |  |
| 1113.6263 | 1113.6204 | -0.0059 | -5 | 611 | 621 | ESVLPAAVTAR |  |  |
| 1124.4967 | 1124.5205 | 0.0238 | 21 | 230 | 239 | NGNTGYDDIR |  |  |
| 1193.5433 | 1193.572 | 0.0287 | 24 | 359 | 369 | YTPESPGDATR |  |  |
| 1193.5433 | 1193.572 | 0.0287 | 24 | 359 | 369 | YTPESPGDATR | 80 |  |
| 1221.6475 | 1221.6621 | 0.0146 | 12 | 257 | 268 | VTTTIGFGSPNK |  |  |
| 1267.5967 | 1267.6323 | 0.0356 | 28 | 63 | 71 | NPYWFNRDR |  |  |
| 1361.6808 | 1361.7036 | 0.0228 | 17 | 269 | 282 | ANSYSVHGSALGAK |  |  |
| 1389.7373 | 1389.7511 | 0.0138 | 10 | 622 | 634 | ISIEAGSTLGWQK |  |  |
| 1389.7373 | 1389.7511 | 0.0138 | 10 | 622 | 634 | ISIEAGSTLGWQK | 86 |  |
| 1464.7944 | 1464.7886 | -0.0058 | -4 | 660 | 673 | EYGITVESIIAAAK |  |  |
| 1476.8057 | 1476.8082 | 0.0025 | 2 | 536 | 549 | LPHLPGTSIEGVEK |  |  |
| 1573.6952 | 1573.6798 | -0.0154 | -10 | 403 | 415 | MFGDFQKDTAEER |  |  |
| 1573.6952 | 1573.8207 | 0.1255 | 80 | 403 | 415 | MFGDFQKDTAEER |  |  |
| 1573.6952 | 1573.8207 | 0.1255 | 80 | 403 | 415 | MFGDFQKDTAEER | 86 |  |
| 1589.6901 | 1589.7318 | 0.0417 | 26 | 403 | 415 | MFGDFQKDTAEER |  | Oxidation (M)[1] |
| 1589.6901 | 1589.7318 | 0.0417 | 26 | 403 | 415 | MFGDFQKDTAEER | 66 | Oxidation (M)[1] |
| 1601.8857 | 1601.8757 | -0.01 | -6 | 10 | 24 | AATGELLEKSVNTIR |  |  |
| 1609.7605 | 1609.72 | -0.0405 | -25 | 311 | 325 | HTPEGAALEADWNAK |  |  |
| 1609.7605 | 1609.8729 | 0.1124 | 70 | 311 | 325 | HTPEGAALEADWNAK |  |  |
| 1732.9592 | 1732.9625 | 0.0033 | 2 | 534 | 549 | QKLPHLPGTSIEGVEK |  |  |
| 1779.964 | 1779.9934 | 0.0294 | 17 | 642 | 659 | AIGIDKFGASAPAGTIYK |  |  |
| 1796.9794 | 1796.9873 | 0.0079 | 4 | 342 | 358 | SIITGELPTGWVDALPK |  |  |
| 1796.9794 | 1796.9873 | 0.0079 | 4 | 342 | 358 | SIITGELPTGWVDALPK | 102 |  |
| 2054.9858 | 2054.9749 | -0.0109 | -5 | 289 | 305 | QNLGWPYDTFFVPEDVK |  |  |
| 2233.1104 | 2233.2266 | 0.1162 | 52 | 497 | 517 | AMPNILMLRPADGNETAGAYK |  |  |
| 2249.1052 | 2249.1362 | 0.031 | 14 | 497 | 517 | AMPNILMLRPADGNETAGAYK |  | Oxidation (M)[2] |
| 2740.3254 | 2740.4233 | 0.0979 | 36 | 283 | 305 | EVEATRQNLGWPYDTFFVPEDVK |  |  |
| 3038.4988 | 3038.574 | 0.0752 | 25 | 550 | 579 | GGYTISDNSTGNKPDLIVMGTGSELEIAAK |  |  |
| 3054.4937 | 3054.5442 | 0.0505 | 17 | 550 | 579 | GGYTISDNSTGNKPDLIVMGTGSELEIAAK |  | Oxidation (M)[19] |
| 3057.4324 | 3057.5452 | 0.1128 | 37 | 191 | 217 | LIAFYDDNHISIDGDTEIAFTEDVSTR |  |  |
| 3301.6265 | 3301.7659 | 0.1394 | 42 | 593 | 621 | VVSFVSWELFDEQSDEYKESVLPAAVTAR |  |  |
| 3301.6265 | 3301.7659 | 0.1394 | 42 | 593 | 621 | VVSFVSWELFDEQSDEYKESVLPAAVTAR | 154 |  |
| 3471.7754 | 3471.9031 | 0.1277 | 37 | 110 | 144 | TPGHPENFETPGVEVTT |  |  |

| **Gel Idx/Pos** | **894** |  |  |  |  |  |  |  |
| --- | --- | --- | --- | --- | --- | --- | --- | --- |
| **Accession No.** | **Protein Name** | **Protein PI** | **Protein MW** | **Protein Score** | **Protein Score C. I. %** | **Total Ion Score** | **Total Ion C. I. %** | **Pep. Count** |
| gi|326378667 | histidine triad nucleotide binding protein [Zea mays] | 6.4 | 15250.6 | 124 | 100 | 158 | 100 | 4 |
| **Peptide Information** | | | | | | | | |
| **Calc. Mass** | **Obsrv. Mass** | **± da** | **± ppm** | **Start Seq.** | **End Seq.** | **Sequence** | **Total Ion Score** | **Modification** |
| 949.4738 | 949.4692 | -0.0046 | -5 | 85 | 89 | KVVAK |  |  |
| 949.4738 | 949.4692 | -0.0046 | -5 | 27 | 38 | EIPSTVVYEDEK |  |  |
| 957.5727 | 957.5565 | -0.0162 | -17 | 27 | 38 | EIPSTVVYEDEK | 69 |  |
| 1609.8909 | 1609.9384 | 0.0475 | 30 | 61 | 67 | DGLTGLAK |  |  |
| 1609.8909 | 1609.9384 | 0.0475 | 30 | 59 | 72 | VKDGLTGLAKAEER | 78 |  |
| 1737.9858 | 1738.0491 | 0.0633 | 36 | 59 | 72 | VKDGLTGLAKAEER |  |  |

| **Gel Idx/Pos** | **1643** |  |  |  |  |  |  |  |
| --- | --- | --- | --- | --- | --- | --- | --- | --- |
| **Accession No.** | **Protein Name** | **Protein PI** | **Protein MW** | **Protein Score** | **Protein Score C. I. %** | **Total Ion Score** | **Total Ion C. I. %** | **Pep. Count** |
| gi|14276718 | T-cytoplasm male sterility restorer factor 2 [Zea mays] | 6.9 | 59772.9 | 886 | 100 | 716 | 100 | 21 |
| **Peptide Information** | | | | | | | | |
| **Calc. Mass** | **Obsrv. Mass** | **± da** | **± ppm** | **Start Seq.** | **End Seq.** | **Sequence** | **Total Ion Score** | **Modification** |
| 835.456 | 835.4655 | 0.0095 | 11 | 139 | 145 | FADLIEK |  |  |
| 870.5658 | 870.5464 | -0.0194 | -22 | 300 | 307 | IILELAAK |  |  |
| 917.5203 | 917.521 | 0.0007 | 1 | 378 | 385 | VVGDPFRK |  |  |
| 945.5265 | 945.5231 | -0.0034 | -4 | 377 | 384 | RVVGDPFR |  |  |
| 946.4993 | 946.5027 | 0.0034 | 4 | 85 | 92 | TFPTLDPR |  |  |
| 946.4993 | 946.5027 | 0.0034 | 4 | 85 | 92 | TFPTLDPR | 41 |  |
| 983.4727 | 983.5013 | 0.0286 | 29 | 33 | 41 | TVPADGMHR |  |  |
| 1028.4935 | 1028.4944 | 0.0009 | 1 | 362 | 369 | VYDEFVEK |  |  |
| 1028.4935 | 1028.4944 | 0.0009 | 1 | 362 | 369 | VYDEFVEK | 31 |  |
| 1046.4943 | 1046.5098 | 0.0155 | 15 | 118 | 126 | AFDEGPWPK |  |  |
| 1105.6252 | 1105.6139 | -0.0113 | -10 | 458 | 466 | FKDLNEVIK |  |  |
| 1155.5688 | 1155.6493 | 0.0805 | 70 | 32 | 41 | RTVPADGMHR |  | Oxidation (M)[8] |
| 1174.5891 | 1174.597 | 0.0079 | 7 | 117 | 126 | KAFDEGPWPK |  |  |
| 1174.5891 | 1174.597 | 0.0079 | 7 | 117 | 126 | KAFDEGPWPK | 55 |  |
| 1363.7581 | 1363.7469 | -0.0112 | -8 | 526 | 537 | GVDSLKNYLQVK |  |  |
| 1412.7931 | 1412.7273 | -0.0658 | -47 | 227 | 240 | VGPALACGNTLVLK |  | Carbamidomethyl (C)[7] |
| 1412.7931 | 1412.8623 | 0.0692 | 49 | 227 | 240 | VGPALACGNTLVLK |  | Carbamidomethyl (C)[7] |
| 1437.6969 | 1437.6185 | -0.0784 | -55 | 407 | 421 | YGVDGGATLVTGGDR |  |  |
| 1437.6969 | 1437.7529 | 0.056 | 39 | 407 | 421 | YGVDGGATLVTGGDR |  |  |
| 1437.6969 | 1437.7529 | 0.056 | 39 | 407 | 421 | YGVDGGATLVTGGDR | 123 |  |
| 1521.8159 | 1521.7852 | -0.0307 | -20 | 241 | 254 | TAEQTPLSALYISK |  |  |
| 1671.9316 | 1671.9293 | -0.0023 | -1 | 443 | 457 | IAQEEIFGPVQSILK |  |  |
| 1671.9316 | 1671.9293 | -0.0023 | -1 | 443 | 457 | IAQEEIFGPVQSILK | 96 |  |
| 1850.9243 | 1850.9512 | 0.0269 | 15 | 407 | 425 | YGVDGGATLVTGGDRLGDK |  |  |
| 1860.0273 | 1859.9904 | -0.0369 | -20 | 33 | 49 | TVPADGMHRLLPGVLQR |  |  |
| 1895.9094 | 1895.9227 | 0.0133 | 7 | 93 | 110 | TGEVIAHVAEGDAEDINR |  |  |
| 1895.9094 | 1895.9227 | 0.0133 | 7 | 93 | 110 | TGEVIAHVAEGDAEDINR | 175 |  |
| 1945.9365 | 1945.9323 | -0.0042 | -2 | 426 | 442 | GFYIQPTIFSDVQDGMK |  |  |
| 1961.9314 | 1961.8964 | -0.035 | -18 | 426 | 442 | GFYIQPTIFSDVQDGMK |  | Oxidation (M)[16] |
| 2556.2688 | 2556.2427 | -0.0261 | -10 | 468 | 492 | ANASQYGLAAGVFTNSLDTANTLTR |  |  |
| 2556.2688 | 2556.2427 | -0.0261 | -10 | 468 | 492 | ANASQYGLAAGVFTNSL | 197 |  |

| **Gel Idx/Pos** | **931** |  |  |  |  |  |  |  |
| --- | --- | --- | --- | --- | --- | --- | --- | --- |
| **Accession No.** | **Protein Name** | **Protein PI** | **Protein MW** | **Protein Score** | **Protein Score C. I. %** | **Total Ion Score** | **Total Ion C. I. %** | **Pep. Count** |
| gi|219819651 | pyruvate orthophosphate dikinase [Zea mays] | 5.55 | 100312.9 | 191 | 100 | 139 | 100 | 14 |
| **Peptide Information** | | | | | | | | |
| **Calc. Mass** | **Obsrv. Mass** | **± da** | **± ppm** | **Start Seq.** | **End Seq.** | **Sequence** | **Total Ion Score** | **Modification** |
| 809.4403 | 809.3907 | -.0496 | -61 | 231 | 237 | EVYLSAK |  |  |
| 836.4373 | 836.4358 | -.0015 | -2 | 861 | 867 | FATERGR |  |  |
| 905.4152 | 905.4095 | -.0057 | -6 | 174 | 180 | FAYDSFR |  |  |
| 905.4152 | 905.4095 | -.0057 | -6 | 174 | 180 | FAYDSFR | 4 |  |
| 973.4625 | 973.4526 | -.0099 | -10 | 238 | 246 | GEPFPSDPK |  |  |
| 1001.5738 | 1001.5645 | -.0093 | -9 | 270 | 278 | SINQITGLR |  |  |
| 1001.5738 | 1001.5645 | -.0093 | -9 | 270 | 278 | SINQITGLR | 46 |  |
| 1019.4714 | 1019.5288 | .0574 | 56 | 332 | 340 | TPEDLDAMK |  |  |
| 1061.5164 | 1061.5093 | -.0071 | -7 | 174 | 181 | FAYDSFRR |  |  |
| 1061.5164 | 1061.5093 | -.0071 | -7 | 174 | 181 | FAYDSFRR | 31 |  |
| 1101.5575 | 1101.5483 | -.0092 | -8 | 238 | 247 | GEPFPSDPKK |  |  |
| 1213.6787 | 1213.6683 | -.0104 | -9 | 34 | 45 | AVVDAAPIQTTK |  |  |
| 1213.6787 | 1213.6683 | -.0104 | -9 | 34 | 45 | AVVDAAPIQTTK | 64 |  |
| 1268.6277 | 1268.5996 | -.0281 | -22 | 24 | 33 | QSRQHCSPLR |  | Carbamidomethyl (C)[6] |
| 1341.7737 | 1341.7662 | -.0075 | -6 | 34 | 46 | AVVDAAPIQTTKK |  |  |
| 1557.7942 | 1557.6819 | -.1123 | -72 | 394 | 407 | IAVDMVNEGLVEPR |  | Oxidation (M)[5] |
| 1626.8738 | 1626.8658 | -.008 | -5 | 224 | 237 | ELVGQYKEVYLSAK |  |  |
| 1685.8026 | 1685.8121 | .0095 | 6 | 182 | 195 | FLDMFGNVVMDIPR |  | Oxidation (M)[4,10] |

| **Gel Idx/Pos** | **931** |  |  |  |  |  |  |  |
| --- | --- | --- | --- | --- | --- | --- | --- | --- |
| **Accession No.** | **Protein Name** | **Protein PI** | **Protein MW** | **Protein Score** | **Protein Score C. I. %** | **Total Ion Score** | **Total Ion C. I. %** | **Pep. Count** |
| gi|11467200 | ribulose-1,5-bisphosphate carboxylase/oxygenase large subunit [Zea mays] | 6.33 | 53294.6 | 950 | 100 | 836 | 100 | 27 |
| **Peptide Information** | | | | | | | | |
| **Calc. Mass** | **Obsrv. Mass** | **± da** | **± ppm** | **Start Seq.** | **End Seq.** | **Sequence** | **Total Ion Score** | **Modification** |
| 912.472 | 912.4847 | 0.0127 | 14 | 296 | 303 | AMHAVIDR |  |  |
| 914.405 | 914.4255 | 0.0205 | 22 | 306 | 312 | NHGMHFR |  | Oxidation (M)[4] |
| 946.4839 | 946.4996 | 0.0157 | 17 | 228 | 236 | AQAETGEIK |  |  |
| 971.4073 | 971.4283 | 0.021 | 22 | 188 | 194 | ACYECLR |  | Carbamidomethyl (C)[2,5] |
| 971.4073 | 971.4283 | 0.021 | 22 | 188 | 194 | ACYECLR | 34 | Carbamidomethyl (C)[2,5] |
| 983.5197 | 983.4901 | -0.0296 | -30 | 464 | 471 | EIKFDGFK |  |  |
| 985.5789 | 985.592 | 0.0131 | 13 | 132 | 139 | ALRLEDLR |  |  |
| 985.5789 | 985.592 | 0.0131 | 13 | 132 | 139 | ALRLEDLR | 35 |  |
| 1021.5312 | 1021.5588 | 0.0276 | 27 | 33 | 41 | DTDILAAFR |  |  |
| 1021.5312 | 1021.5588 | 0.0276 | 27 | 33 | 41 | DTDILAAFR | 72 |  |
| 1037.4899 | 1037.527 | 0.0371 | 36 | 351 | 358 | DDFIEKDR |  |  |
| 1116.583 | 1116.6162 | 0.0332 | 30 | 422 | 431 | VALEACVQAR |  | Carbamidomethyl (C)[6] |
| 1116.583 | 1116.6162 | 0.0332 | 30 | 422 | 431 | VALEACVQAR | 75 | Carbamidomethyl (C)[6] |
| 1170.5586 | 1170.6744 | 0.1158 | 99 | 304 | 312 | QKNHGMHFR |  | Oxidation (M)[6] |
| 1187.6644 | 1187.7054 | 0.041 | 35 | 286 | 295 | DNGLLLHIHR |  |  |
| 1247.6129 | 1247.6428 | 0.0299 | 24 | 218 | 227 | FVFCAEAIYK |  | Carbamidomethyl (C)[4] |
| 1247.6129 | 1247.6428 | 0.0299 | 24 | 218 | 227 | FVFCAEAIYK | 51 | Carbamidomethyl (C)[4] |
| 1257.6797 | 1257.7312 | 0.0515 | 41 | 436 | 446 | DLAREGNEIIK |  |  |
| 1275.7307 | 1275.7723 | 0.0416 | 33 | 340 | 350 | EITLGFVDLLR |  |  |
| 1275.7307 | 1275.7723 | 0.0416 | 33 | 340 | 350 | EITLGFVDLLR | 99 |  |
| 1401.7737 | 1401.8202 | 0.0465 | 33 | 135 | 146 | LEDLRIPPAYSK |  |  |
| 1407.6678 | 1407.7085 | 0.0407 | 29 | 22 | 32 | LTYYTPEYETK |  |  |
| 1451.6219 | 1451.6956 | 0.0737 | 51 | 202 | 213 | DDENVNSQPFMR |  |  |
| 1465.7546 | 1465.817 | 0.0624 | 43 | 147 | 159 | TFQGPPHGIQVER |  |  |
| 1465.7546 | 1465.817 | 0.0624 | 43 | 147 | 159 | TFQGPPHGIQVER | 115 |  |
| 1481.7167 | 1481.8394 | 0.1227 | 83 | 320 | 334 | MSGGDHIHSGTVVGK |  |  |
| 1502.8512 | 1502.9182 | 0.067 | 45 | 165 | 177 | YGRPLLGCTIKPK |  | Carbamidomethyl (C)[8] |
| 1518.741 | 1518.8207 | 0.0797 | 52 | 216 | 227 | DRFVFCAEAIYK |  | Carbamidomethyl (C)[6] |
| 1534.7358 | 1534.7904 | 0.0546 | 36 | 451 | 463 | WSAELAAACEIWK |  | Carbamidomethyl (C)[9] |
| 1860.0226 | 1860.0095 | -0.0131 | -7 | 335 | 350 | LEGEREITLGFVDLLR |  |  |
| 1860.0226 | 1860.16 | 0.1374 | 74 | 335 | 350 | LEGEREITLGFVDLLR |  |  |
| 1950.916 | 1951.0042 | 0.0882 | 45 | 237 | 253 | GHYLNATAGTCEEMIKR |  | Carbamidomethyl (C)[11] |
| 2023.0747 | 2023.1958 | 0.1211 | 60 | 340 | 356 | EITLGFVDLLRDDFIEK |  |  |
| 2169.9871 | 2170.1069 | 0.1198 | 55 | 195 | 213 | GGLDFTKDDENVNSQPFMR |  |  |
| 2169.9871 | 2170.1069 | 0.1198 | 55 | 195 | 213 | GGLDFTKDDENVNSQPFMR | 131 |  |
| 2185.9819 | 2186.0103 | 0.0284 | 13 | 195 | 213 | GGLDFTKDDENVNSQPFMR |  | Oxidation (M)[18] |
| 2410.1814 | 2410.3411 | 0.1597 | 66 | 22 | 41 | LTYYTPEYETKDTDILAAFR |  |  |
| 2410.1814 | 2410.3411 | 0.1597 | 66 | 22 | 41 | LTYYTPEYETKDTDILAAFR | 148 |  |

| **Gel Idx/Pos** | **2056** |  |  |  |  |  |  |  |
| --- | --- | --- | --- | --- | --- | --- | --- | --- |
| **Accession No.** | **Protein Name** | **Protein PI** | **Protein MW** | **Protein Score** | **Protein Score C. I. %** | **Total Ion Score** | **Total Ion C. I. %** | **Pep. Count** |
| gi|226530305 | Ascorbate Peroxidase [Zea mays] | 5.55 | 27461.9 | 110 | 100 | 54 | 98.732 | 8 |
| **Peptide Information** | | | | | | | | |
| **Calc. Mass** | **Obsrv. Mass** | **± da** | **± ppm** | **Start Seq.** | **End Seq.** | **Sequence** | **Total Ion Score** | **Modification** |
| 905.404 | 905 |  | 69 | 23000% | 236 | AFFDDYK |  |  |
| 911.429 | 911 |  | 34 | 5300% | 61 | TGGPFGTMK |  | Oxidation (M)[8] |
| 911.429 | 911 |  | 34 | 5300% | 61 | TGGPFGTMK |  | Oxidation (M)[8] |
| 938.447 | 938 |  | - 53 | 24200% | 250 | LSELGYADA |  |  |
| 1249.617 | 1250 |  | - 1 | 12000% | 130 | EDKPQPPPEGR |  |  |
| 1249.617 | 1250 |  | - 1 | 12000% | 130 | EDKPQPPPEGR | 28 |  |
| 1309.686 | 1310 |  | - 10 | 13100% | 142 | LPDATKGSDHLR |  |  |
| 1309.686 | 1310 |  | - 10 | 13100% | 142 | LPDATKGSDHLR | 11 |  |
| 1483.722 | 1484 |  | 15 | 23000% | 241 | AFFDDYKEAHLK |  |  |
| 1583.916 | 1584 |  | 5 | 21000% | 223 | ALLSDPVFRPLVEK |  |  |
| 1583.916 | 1584 |  | 5 | 21000% | 223 | ALLSDPVFRPLVEK | 15 |  |
| 2071.024 | 2071 |  | - 1 | 14800% | 167 | QMGLSDQDIVALSGGHTLGR |  | Oxidation (M)[2] |

| **Gel Idx/Pos** | **1812** |  |  |  |  |  |  |  |
| --- | --- | --- | --- | --- | --- | --- | --- | --- |
| **Accession No.** | **Protein Name** | **Protein PI** | **Protein MW** | **Protein Score** | **Protein Score C. I. %** | **Total Ion Score** | **Total Ion C. I. %** | **Pep. Count** |
| gi|226496743 | 50S ribosomal protein L1 [Zea mays] | 8.69 | 37235.2 | 714 | 100 | 567 | 100 | 16 |
| **Peptide Information** | | | | | | | | |
| **Calc. Mass** | **Obsrv. Mass** | **± da** | **± ppm** | **Start Seq.** | **End Seq.** | **Sequence** | **Total Ion Score** | **Modification** |
| 936.4533 | 936.4403 | -0.013 | -14 | 161 | 167 | YNDQQLR |  |  |
| 936.4533 | 936.4403 | -0.013 | -14 | 161 | 167 | YNDQQLR | 42 |  |
| 958.5568 | 958.5397 | -0.0171 | -18 | 182 | 190 | IAVLTQGEK |  |  |
| 1046.6357 | 1046.6147 | -0.021 | -20 | 115 | 122 | FLEIQKLR |  |  |
| 1054.6045 | 1054.5798 | -0.0247 | -23 | 275 | 284 | SGIVHIPFGK |  |  |
| 1102.5635 | 1102.5914 | 0.0279 | 25 | 221 | 230 | LIASPDMMPK |  |  |
| 1170.6378 | 1170.6414 | 0.0036 | 3 | 87 | 97 | GPAFTAPTRPR |  |  |
| 1170.6378 | 1170.6414 | 0.0036 | 3 | 87 | 97 | GPAFTAPTRPR | 50 |  |
| 1192.5746 | 1192.5806 | 0.006 | 5 | 145 | 154 | FVESAEAHFR |  |  |
| 1192.5746 | 1192.5806 | 0.006 | 5 | 145 | 154 | FVESAEAHFR | 73 |  |
| 1396.7948 | 1396.7623 | -0.0325 | -23 | 272 | 284 | VDKSGIVHIPFGK |  |  |
| 1480.7716 | 1480.7678 | -0.0038 | -3 | 126 | 138 | KDYDVPTAISLMK |  |  |
| 1542.8486 | 1542.8802 | 0.0316 | 20 | 182 | 195 | IAVLTQGEKIDEAR |  |  |
| 1542.8486 | 1542.8802 | 0.0316 | 20 | 182 | 195 | IAVLTQGEKIDEAR | 101 |  |
| 1634.7955 | 1634.8448 | 0.0493 | 30 | 155 | 167 | MNLDPKYNDQQLR |  |  |
| 1694.8596 | 1694.8516 | -0.008 | -5 | 249 | 264 | AGTVSSNITQAIEEFK |  |  |
| 1698.8909 | 1698.9025 | 0.0116 | 7 | 196 | 212 | AAGADIVGGEDLIEQIK |  |  |
| 1698.8909 | 1698.9025 | 0.0116 | 7 | 196 | 212 | AAGADIVGGEDLIEQIK | 92 |  |
| 1726.8204 | 1726.8129 | -0.0075 | -4 | 331 | 345 | LNIKEMLDYGLESSD |  |  |
| 1822.9546 | 1822.9646 | 0.01 | 5 | 249 | 265 | AGTVSSNITQAIEEFKK |  |  |
| 1822.9546 | 1822.9646 | 0.01 | 5 | 249 | 265 | AGTVSSNITQAIEEFKK | 86 |  |
| 1840.8647 | 1840.8828 | 0.0181 | 10 | 139 | 154 | QMSSAKFVESAEAHFR |  | Oxidation (M)[2] |
| 1980.9736 | 1981.0502 | 0.0766 | 39 | 285 | 301 | VDFPEEDLIANFMSVVR |  |  |
| 1980.9736 | 1981.0502 | 0.0766 | 39 | 285 | 301 | VDFPEEDLIANFMSVVR | 122 |  |
| 1996.9685 | 1996.991 | 0.0225 | 11 | 285 | 301 | VDFPEEDLIANFMSVVR |  | Oxidation (M)[13] |

| **Gel Idx/Pos** | **739** |  |  |  |  |  |  |  |
| --- | --- | --- | --- | --- | --- | --- | --- | --- |
| **Accession No.** | **Protein Name** | **Protein PI** | **Protein MW** | **Protein Score** | **Protein Score C. I. %** | **Total Ion Score** | **Total Ion C. I. %** | **Pep. Count** |
| gi|226508112 | cysteine synthase1 [Zea mays] | 6.97 | 41839.6 | 394 | 100 | 345 | 100 | 8 |
| **Peptide Information** | | | | | | | | |
| **Calc. Mass** | **Obsrv. Mass** | **± da** | **± ppm** | **Start Seq.** | **End Seq.** | **Sequence** | **Total Ion Score** | **Modification** |
| 1114.6368 | 1114.6366 | -0.0002 | 0 | 299 | 309 | IQGIGAGFVPR |  |  |
| 1114.6368 | 1114.6366 | -0.0002 | 0 | 299 | 309 | IQGIGAGFVPR | 80 |  |
| 1305.705 | 1305.6831 | -0.0219 | -17 | 187 | 199 | AFGAELVLTDAAK |  |  |
| 1305.705 | 1305.6831 | -0.0219 | -17 | 187 | 199 | AFGAELVLTDAAK | 48 |  |
| 1362.7781 | 1362.7972 | 0.0191 | 14 | 366 | 377 | LIVVVFPSFGER |  |  |
| 1362.7781 | 1362.7972 | 0.0191 | 14 | 366 | 377 | LIVVVFPSFGER | 32 |  |
| 1368.6464 | 1368.5912 | -0.0552 | -40 | 125 | 136 | IGYSMINDAEQK |  |  |
| 1384.6414 | 1384.6489 | 0.0075 | 5 | 125 | 136 | IGYSMINDAEQK |  | Oxidation (M)[5] |
| 1747.9338 | 1747.9862 | 0.0524 | 30 | 249 | 267 | VDIFIGGIGTGGTISGAGR |  |  |
| 1747.9338 | 1747.9862 | 0.0524 | 30 | 249 | 267 | VDIFIGGIGTGGTISGAGR | 56 |  |
| 1933.0503 | 1933.1121 | 0.0618 | 32 | 247 | 267 | GKVDIFIGGIGTGGTISGAGR |  |  |
| 1933.0503 | 1933.1121 | 0.0618 | 32 | 247 | 267 | GKVDIFIGGIGTGGTISGAGR | 15 |  |
| 2505.209 | 2505.2454 | 0.0364 | 15 | 310 | 332 | NLDSDILDEVIEISSDEAVETAK |  |  |
| 2505.209 | 2505.2454 | 0.0364 | 15 | 310 | 332 | NLDSDILDEVIEISSDEAVETAK | 114 |  |
| 2572.1694 | 2572.2051 | 0.0357 | 14 | 378 | 398 | YLSSVLYQSIREECENMQPEP |  | Carbamidomethyl (C)[14] |
| 2588.1643 | 2588.2527 | 0.0884 | 34 | 378 | 398 | YLSSVLYQSIREECENMQPEP |  | Carbamidomethyl (C)[14], Oxidation (M)[17] |

| **Gel Idx/Pos** | **1685** |  |  |  |  |  |  |  |
| --- | --- | --- | --- | --- | --- | --- | --- | --- |
| **Accession No.** | **Protein Name** | **Protein PI** | **Protein MW** | **Protein Score** | **Protein Score C. I. %** | **Total Ion Score** | **Total Ion C. I. %** | **Pep. Count** |
| gi|357123797 | PREDICTED: peroxiredoxin-2E-1, chloroplastic-like [Brachypodium distachyon] | 8.58 | 23757.5 | 83 | 99.434 | 50 | 95.773 | 5 |
| **Peptide Information** | | | | | | | | |
| **Calc. Mass** | **Obsrv. Mass** | **± da** | **± ppm** | **Start Seq.** | **End Seq.** | **Sequence** | **Total Ion Score** | **Modification** |
| 868.504 | 868.4864 | -0.0176 | -20 | 121 | 128 | HLPGFVAK |  |  |
| 924.5737 | 924.4985 | -0.0752 | -81 | 45 | 53 | AAGLLRAPR |  |  |
| 1177.6464 | 1177.6287 | -0.0177 | -15 | 198 | 208 | YALLAEDGVVK |  |  |
| 1333.7474 | 1333.7642 | 0.0168 | 13 | 197 | 208 | RYALLAEDGVVK |  |  |
| 1333.7474 | 1333.7642 | 0.0168 | 13 | 197 | 208 | RYALLAEDGVVK | 4 |  |
| 1871.9896 | 1871.9785 | -0.0111 | -6 | 177 | 194 | AMGVELDLSDKPVGLGVR |  | Oxidation (M)[2] |
| 1871.9896 | 1871.9785 | -0.0111 | -6 | 177 | 194 | AMGVELDLSDKPVGLGVR | 50 | Oxidation (M)[2] |

| **Gel Idx/Pos** | **1713** |  |  |  |  |  |  |  |
| --- | --- | --- | --- | --- | --- | --- | --- | --- |
| **Accession No.** | **Protein Name** | **Protein PI** | **Protein MW** | **Protein Score** | **Protein Score C. I. %** | **Total Ion Score** | **Total Ion C. I. %** | **Pep. Count** |
| gi|226507400 | aconitase [Zea mays] | 6.04 | 99565.3 | 321 | 100 | 363 | 100 | 23 |
| **Peptide Information** | | | | | | | | |
| **Calc. Mass** | **Obsrv. Mass** | **± da** | **± ppm** | **Start Seq.** | **End Seq.** | **Sequence** | **Total Ion Score** | **Modification** |
| 884.4294 | 884.4355 | 0.0061 | 7 | 871 | 877 | SFTCTLR |  | Carbamidomethyl (C)[4] |
| 900.5513 | 900.5433 | -0.008 | -9 | 49 | 56 | VLLESAIR |  |  |
| 905.5203 | 905.5178 | -0.0025 | -3 | 588 | 595 | GGKEVFLR |  |  |
| 1012.4768 | 1012.4831 | 0.0063 | 6 | 620 | 627 | VYDSIMER |  |  |
| 1028.4718 | 1028.4897 | 0.0179 | 17 | 620 | 627 | VYDSIMER |  | Oxidation (M)[6] |
| 1064.5007 | 1064.5289 | 0.0282 | 26 | 150 | 157 | NEELEFQR |  |  |
| 1104.6412 | 1104.6414 | 0.0002 | 0 | 40 | 48 | IDKLPYSIR |  |  |
| 1104.6412 | 1104.6414 | 0.0002 | 0 | 40 | 48 | IDKLPYSIR | 44 |  |
| 1140.5718 | 1140.5986 | 0.0268 | 23 | 619 | 627 | KVYDSIMER |  |  |
| 1141.6729 | 1141.6754 | 0.0025 | 2 | 81 | 90 | LAEIPFKPAR |  |  |
| 1141.6729 | 1141.6754 | 0.0025 | 2 | 81 | 90 | LAEIPFKPAR | 51 |  |
| 1178.5477 | 1178.5642 | 0.0165 | 14 | 639 | 647 | EALYPWEDR |  |  |
| 1178.5477 | 1178.5642 | 0.0165 | 14 | 639 | 647 | EALYPWEDR | 67 |  |
| 1202.6052 | 1202.6124 | 0.0072 | 6 | 71 | 80 | IIDWENTSPK |  |  |
| 1210.6355 | 1210.6174 | -0.0181 | -15 | 704 | 713 | YLLEYGVEPK |  |  |
| 1261.6019 | 1261.6437 | 0.0418 | 33 | 830 | 842 | AGEDADSLGLTGR |  |  |
| 1276.6685 | 1276.6818 | 0.0133 | 10 | 29 | 39 | FFSLPALNDPR |  |  |
| 1276.6685 | 1276.6818 | 0.0133 | 10 | 29 | 39 | FFSLPALNDPR | 46 |  |
| 1294.6274 | 1294.6548 | 0.0274 | 21 | 138 | 149 | VDVAGTYDALDR |  |  |
| 1316.678 | 1316.6925 | 0.0145 | 11 | 1 | 12 | MANPTATKHAFK |  |  |
| 1392.7271 | 1392.7382 | 0.0111 | 8 | 747 | 759 | AGPWTIHVPTGEK |  |  |
| 1513.7203 | 1513.7455 | 0.0252 | 17 | 334 | 346 | SDETVSMIEAYLR |  |  |
| 1513.7203 | 1513.7455 | 0.0252 | 17 | 334 | 346 | SDETVSMIEAYLR | 92 |  |
| 1517.7053 | 1517.7356 | 0.0303 | 20 | 350 | 361 | MFVDKHEPETER |  |  |
| 1529.7152 | 1529.7494 | 0.0342 | 22 | 334 | 346 | SDETVSMIEAYLR |  | Oxidation (M)[7] |
| 1565.8422 | 1565.8419 | -0.0003 | 0 | 477 | 491 | TSLTPGSVVATEYLK |  |  |
| 1688.9694 | 1688.8354 | -0.134 | -79 | 13 | 28 | RILTSLLKPGGGEYGK |  |  |
| 1741.996 | 1742.0239 | 0.0279 | 16 | 122 | 137 | INPLIPVDAVIDHAVR |  |  |
| 1839.964 | 1840.0135 | 0.0495 | 27 | 285 | 301 | FVEFYGVGVGELSLPAR |  |  |
| 2340.1104 | 2340.1375 | 0.0271 | 12 | 138 | 157 | VDVAGTYDALDRNEELEFQR |  |  |
| 2340.1104 | 2340.1375 | 0.0271 | 12 | 138 | 157 | VDVAGTYDALDRNEELEFQR | 63 |  |

| **Gel Idx/Pos** | **1227** |  |  |  |  |  |  |  |
| --- | --- | --- | --- | --- | --- | --- | --- | --- |
| **Accession No.** | **Protein Name** | **Protein PI** | **Protein MW** | **Protein Score** | **Protein Score C. I. %** | **Total Ion Score** | **Total Ion C. I. %** | **Pep. Count** |
| gi|195623400 | chaperonin [Zea mays] | 8.67 | 25558.7 | 235 | 100 | 152 | 100 | 10 |
| **Peptide Information** | | | | | | | | |
| **Calc. Mass** | **Obsrv. Mass** | **± da** | **± ppm** | **Start Seq.** | **End Seq.** | **Sequence** | **Total Ion Score** | **Modification** |
| 801.3777 | 801.3841 | 0.0064 | 8 | 220 | 226 | YAGSEFK |  |  |
| 803.3893 | 803.4099 | 0.0206 | 26 | 164 | 170 | VAEAEER |  |  |
| 892.4444 | 892.4343 | -0.0101 | -11 | 237 | 245 | ASDVMAVLS |  |  |
| 988.488 | 988.5138 | 0.0258 | 26 | 152 | 159 | DMKPLNDR |  |  |
| 1004.4829 | 1004.5254 | 0.0425 | 42 | 152 | 159 | DMKPLNDR |  | Oxidation (M)[2] |
| 1149.6263 | 1149.6399 | 0.0136 | 12 | 52 | 61 | YTSIKPLGDR |  |  |
| 1149.6263 | 1149.6399 | 0.0136 | 12 | 52 | 61 | YTSIKPLGDR | 38 |  |
| 1173.6838 | 1173.6774 | -0.0064 | -5 | 171 | 182 | TAGGLLLTQATK |  |  |
| 1932.9338 | 1932.9729 | 0.0391 | 20 | 220 | 236 | YAGSEFKGEDGEYIVLR |  |  |
| 1932.9338 | 1932.9729 | 0.0391 | 20 | 220 | 236 | YAGSEFKGEDGEYIVLR | 92 |  |
| 2094.0825 | 2094.0588 | -0.0237 | -11 | 183 | 204 | EKPSVGSVVAVGPGPLGEDGSR |  |  |
| 2237.1926 | 2237.1926 | 0 | 0 | 100 | 120 | SFGSNRIEISVPVGAQVVYSK |  |  |
| 2691.4424 | 2691.4441 | 0.0017 | 1 | 73 | 99 | SDGGILLPVSVQTRPQGGEIVAVGEGR |  |  |
| 2691.4424 | 2691.4441 | 0.0017 | 1 | 73 | 99 | SDGGILLPVSVQTRPQGGEIVAVGEGR | 22 |  |

| **Gel Idx/Pos** | **1619** |  |  |  |  |  |  |  |
| --- | --- | --- | --- | --- | --- | --- | --- | --- |
| **Accession No.** | **Protein Name** | **Protein PI** | **Protein MW** | **Protein Score** | **Protein Score C. I. %** | **Total Ion Score** | **Total Ion C. I. %** | **Pep. Count** |
| gi|162460411 | glutathione S-transferase 3 [Zea mays] | 6.05 | 23891.6 | 100 |  | 99.99 | 70 | 5 |
| **Peptide Information** | | | | | | | | |
| **Calc. Mass** | **Obsrv. Mass** | **± da** | **± ppm** | **Start Seq.** | **End Seq.** | **Sequence** | **Total Ion Score** | **Modification** |
| 873.504 | 873.4768 | -.0272 | -31 | 18 | 25 | VATVLNEK |  |  |
| 1285.6899 | 1285.7153 | .0254 | 20 | 143 | 153 | VLDVYEAHLAR |  |  |
| 1285.6899 | 1285.7153 | .0254 | 20 | 143 | 153 | VLDVYEAHLAR | 70 |  |
| 1361.7246 | 1361.7527 | .0281 | 21 | 6 | 17 | LYGMPLSPNVVR |  | Oxidation (M)[4] |
| 1665.833 | 1665.9178 | .0848 | 51 | 79 | 95 | YASEGTDLLPATASAAK |  |  |
| 1811.9539 | 1812.0364 | .0825 | 46 | 26 | 42 | GLDFEIVPVDLTTGAHK |  |  |

| **Gel Idx/Pos** | **1867** |  |  |  |  |  |  |  |
| --- | --- | --- | --- | --- | --- | --- | --- | --- |
| **Accession No.** | **Protein Name** | **Protein PI** | **Protein MW** | **Protein Score** | **Protein Score C. I. %** | **Total Ion Score** | **Total Ion C. I. %** | **Pep. Count** |
| gi|50952841 | RuBisCo subunit binding-protein beta subunit [Zea mays] | 4.75 | 44275 | 450 | 100 | 345 | 100 | 14 |
| **Peptide Information** | | | | | | | | |
| **Calc. Mass** | **Obsrv. Mass** | **± da** | **± ppm** | **Start Seq.** | **End Seq.** | **Sequence** | **Total Ion Score** | **Modification** |
| 899.5421 | 899.5203 | -0.0218 | -24 | 303 | 310 | VGAEIVRR |  |  |
| 936.4785 | 936.4668 | -0.0117 | -12 | 334 | 341 | VLSNDNFK |  |  |
| 947.5672 | 947.5438 | -0.0234 | -25 | 310 | 317 | RALSYPLK |  |  |
| 1229.7001 | 1229.6954 | -0.0047 | -4 | 136 | 147 | IAAIKAPGFGER |  |  |
| 1229.7001 | 1229.6954 | -0.0047 | -4 | 136 | 147 | IAAIKAPGFGER | 42 |  |
| 1245.5715 | 1245.6232 | 0.0517 | 42 | 368 | 378 | CCLEHAASVAK |  | Carbamidomethyl (C)[1,2] |
| 1284.7158 | 1284.7261 | 0.0103 | 8 | 95 | 105 | DLINVLEEAIR |  |  |
| 1284.7158 | 1284.7261 | 0.0103 | 8 | 95 | 105 | DLINVLEEAIR | 97 |  |
| 1505.7159 | 1505.7134 | -0.0025 | -2 | 61 | 73 | GYISPYFVTDSEK |  |  |
| 1693.8279 | 1693.8313 | 0.0034 | 2 | 214 | 227 | NLIEAAEQEYEKEK |  |  |
| 1700.9 | 1700.9456 | 0.0456 | 27 | 268 | 284 | AAVEEGIVVGGGCTLLR |  | Carbamidomethyl (C)[13] |
| 1700.9 | 1700.9456 | 0.0456 | 27 | 268 | 284 | AAVEEGIVVGGGCTLLR | 124 | Carbamidomethyl (C)[13] |
| 1884.0437 | 1883.9796 | -0.0641 | -34 | 235 | 253 | LAGGVAVIQVGAQTETELK |  |  |
| 1920.8796 | 1920.9535 | 0.0739 | 38 | 45 | 60 | SSENFLYVVEGMQFDR |  |  |
| 1920.8796 | 1920.9535 | 0.0739 | 38 | 45 | 60 | SSENFLYVVEGMQFDR | 45 |  |
| 1936.9459 | 1936.9326 | -0.0133 | -7 | 191 | 208 | ESTTIVGDGSTQEEVTKR |  |  |
| 1936.9459 | 1936.9326 | -0.0133 | -7 | 191 | 208 | ESTTIVGDGSTQEEVTKR | 41 |  |
| 2141.1812 | 2141.1406 | -0.0406 | -19 | 235 | 255 | LAGGVAVIQVGAQTETELKEK |  |  |
| 2462.1545 | 2462.1809 | 0.0264 | 11 | 342 | 364 | YGYNAATGQYEDLMAAGIIDPTK |  |  |
| 2478.1494 | 2478.2334 | 0.084 | 34 | 342 | 364 | YGYNAATGQYEDLMAAGIIDPTK |  | Oxidation (M)[14] |

| **Gel Idx/Pos** | **1812** |  |  |  |  |  |  |  |
| --- | --- | --- | --- | --- | --- | --- | --- | --- |
| **Accession No.** | **Protein Name** | **Protein PI** | **Protein MW** | **Protein Score** | **Protein Score C. I. %** | **Total Ion Score** | **Total Ion C. I. %** | **Pep. Count** |
| gi|226502947 | S-adenosylmethionine synthetase 1 [Zea mays] | 5.5 | 42985.6 | 699 | 100 | 587 | 100 | 14 |
| **Peptide Information** | | | | | | | | |
| **Calc. Mass** | **Obsrv. Mass** | **± da** | **± ppm** | **Start Seq.** | **End Seq.** | **Sequence** | **Total Ion Score** | **Modification** |
| 873.5152 | 873.5251 | 0.0099 | 11 | 294 | 302 | SIVASGLAR |  |  |
| 873.5152 | 873.5251 | 0.0099 | 11 | 294 | 302 | SIVASGLAR | 44 |  |
| 919.4778 | 919.4993 | 0.0215 | 23 | 70 | 76 | IVRDTCR |  | Carbamidomethyl (C)[6] |
| 979.4744 | 979.4878 | 0.0134 | 14 | 366 | 374 | TAAYGHFGR |  |  |
| 979.4744 | 979.4878 | 0.0134 | 14 | 366 | 374 | TAAYGHFGR | 54 |  |
| 1141.6113 | 1141.6335 | 0.0222 | 19 | 230 | 239 | TIFHLNPSGR |  |  |
| 1141.6113 | 1141.6335 | 0.0222 | 19 | 230 | 239 | TIFHLNPSGR | 61 |  |
| 1316.7686 | 1316.7198 | -0.0488 | -37 | 214 | 224 | EHVIKPVIPER |  |  |
| 1316.7686 | 1316.8386 | 0.07 | 53 | 214 | 224 | EHVIKPVIPER |  |  |
| 1453.7548 | 1453.8062 | 0.0514 | 35 | 240 | 254 | FVIGGPHGDAGLTGR |  |  |
| 1453.7548 | 1453.8062 | 0.0514 | 35 | 240 | 254 | FVIGGPHGDAGLTGR | 108 |  |
| 1470.7332 | 1470.762 | 0.0288 | 20 | 50 | 62 | TNMVMVFGEITTK |  |  |
| 1581.8496 | 1581.8053 | -0.0443 | -28 | 240 | 255 | FVIGGPHGDAGLTGRK |  |  |
| 1609.7341 | 1609.7131 | -0.021 | -13 | 77 | 91 | EIGFTSDDVGLDADR |  |  |
| 1609.7341 | 1609.854 | 0.1199 | 74 | 77 | 91 | EIGFTSDDVGLDADR |  |  |
| 1609.7341 | 1609.854 | 0.1199 | 74 | 77 | 91 | EIGFTSDDVGLDADR | 98 |  |
| 1789.9232 | 1790.0048 | 0.0816 | 46 | 225 | 239 | YLDEKTIFHLNPSGR |  |  |
| 1897.8597 | 1897.9097 | 0.05 | 26 | 77 | 93 | EIGFTSDDVGLDADRCK |  | Carbamidomethyl (C)[16] |
| 1897.8597 | 1897.9097 | 0.05 | 26 | 77 | 93 | EIGFTSDDVGLDADRCK | 113 | Carbamidomethyl (C)[16] |
| 1948.9797 | 1949.0635 | 0.0838 | 43 | 172 | 189 | TQVTVEYVNEGGAMVPVR |  |  |
| 1948.9797 | 1949.0635 | 0.0838 | 43 | 172 | 189 | TQVTVEYVNEGGAMVPVR | 110 |  |
| 1964.9746 | 1965.0028 | 0.0282 | 14 | 172 | 189 | TQVTVEYVNEGGAMVPVR |  | Oxidation (M)[14] |
| 2417.2573 | 2417.2869 | 0.0296 | 12 | 94 | 115 | VLVNIEQQSPDIAQGVHGHFTK |  |  |
| 2649.3367 | 2649.387 | 0.0503 | 19 | 190 | 213 | VHTVLISTQHDETVTNDEI |  |  |

| **Gel Idx/Pos** | **1646** |  |  |  |  |  |  |  |
| --- | --- | --- | --- | --- | --- | --- | --- | --- |
| **Accession No.** | **Protein Name** | **Protein PI** | **Protein MW** | **Protein Score** | **Protein Score C. I. %** | **Total Ion Score** | **Total Ion C. I. %** | **Pep. Count** |
| gi|109892850 | RecName: Full=Putative cytochrome c oxidase subunit II PS17 | 9.63 | 1707 | 74 | 96.172 | 51 | 96.544 | 2 |
| **Peptide Information** | | | | | | | | |
| **Calc. Mass** | **Obsrv. Mass** | **± da** | **± ppm** | **Start Seq.** | **End Seq.** | **Sequence** | **Total Ion Score** | **Modification** |
| 856.5251 | 856.4947 | -0.0304 | -35 | 1 | 8 | SPTVIALR |  |  |
| 856.5251 | 856.4947 | -0.0304 | -35 | 1 | 8 | SPTVIALR | 25 |  |
| 870.5043 | 870.5315 | 0.0272 | 31 | 9 | 16 | VVEALSPR |  |  |
| 870.5043 | 870.5315 | 0.0272 | 31 | 9 | 16 | VVEALSPR | 26 |  |

| **Gel Idx/Pos** | **481** |  |  |  |  |  |  |  |
| --- | --- | --- | --- | --- | --- | --- | --- | --- |
| **Accession No.** | **Protein Name** | **Protein PI** | **Protein MW** | **Protein Score** | **Protein Score C. I. %** | **Total Ion Score** | **Total Ion C. I. %** | **Pep. Count** |
| gi|212722236 | uncharacterized protein LOC100193292 [Zea mays] | 8.89 | 41352.4 | 137 | 100 | 44 | 91.265 | 13 |
| **Peptide Information** | | | | | | | | |
| **Calc. Mass** | **Obsrv. Mass** | **± da** | **± ppm** | **Start Seq.** | **End Seq.** | **Sequence** | **Total Ion Score** | **Modification** |
| 824.4182 | 824.4393 | 0.0211 | 26 | 1 | 8 | MAATSSLK |  | Oxidation (M)[1] |
| 870.4355 | 870.5016 | 0.0661 | 76 | 302 | 308 | EFDFGKK |  |  |
| 870.4355 | 870.5016 | 0.0661 | 76 | 302 | 308 | EFDFGKK |  |  |
| 960.5295 | 960.4794 | -0.0501 | -52 | 51 | 59 | NILVMGGTR |  |  |
| 977.5414 | 977.4908 | -0.0506 | -52 | 265 | 273 | QIFNISGAK |  |  |
| 1041.5364 | 1041.5122 | -0.0242 | -23 | 274 | 282 | YVTFDGLAR |  |  |
| 1041.5364 | 1041.5122 | -0.0242 | -23 | 274 | 282 | YVTFDGLAR | 41 |  |
| 1072.615 | 1072.5691 | -0.0459 | -43 | 252 | 261 | AFNLVLGNPK |  |  |
| 1187.6168 | 1187.5885 | -0.0283 | -24 | 72 | 81 | EGHQVTLFTR |  |  |
| 1240.5957 | 1240.6423 | 0.0466 | 38 | 110 | 119 | GDRQDFEFVK |  |  |
| 1641.7677 | 1641.6993 | -0.0684 | -42 | 357 | 370 | EADFTTDDMILDKK |  |  |
| 1654.8224 | 1654.792 | -0.0304 | -18 | 287 | 301 | AGGFPEPELVHYNPK |  |  |
| 1694.8054 | 1694.7776 | -0.0278 | -16 | 166 | 180 | SDILPHCEVDAVDPK |  | Carbamidomethyl (C)[7] |
| 1827.8872 | 1827.8579 | -0.0293 | -16 | 120 | 136 | TSLAANGYDVVYDINGR |  |  |
| 2439.1675 | 2439.1987 | 0.0312 | 13 | 82 | 104 | GKAPITQQLPGESDAEYADFSSK |  |  |
| 2439.1675 | 2439.1987 | 0.0312 | 13 | 82 | 104 | GKAPITQQLPGESDAEYADFSSK | 4 |  |

| **Gel Idx/Pos** | **983** |  |  |  |  |  |  |  |
| --- | --- | --- | --- | --- | --- | --- | --- | --- |
| **Accession No.** | **Protein Name** | **Protein PI** | **Protein MW** | **Protein Score** | **Protein Score C. I. %** | **Total Ion Score** | **Total Ion C. I. %** | **Pep. Count** |
| gi|357151031 | PREDICTED: probable disease resistance RPP8-like protein 4-like [Brachypodium distachyon] | 5.6 | 154698.7 | 75 | 96.742 | 89 | 99 | 18 |
| **Peptide Information** | | | | | | | | |
| **Calc. Mass** | **Obsrv. Mass** | **± da** | **± ppm** | **Start Seq.** | **End Seq.** | **Sequence** | **Total Ion Score** | **Modification** |
| 870.5268 | 870.5596 | 0.0328 | 38 | 1104 | 1111 | RGGLQALR |  |  |
| 1018.5655 | 1018.5663 | 0.0008 | 1 | 67 | 73 | WWMKLVR |  |  |
| 1027.5895 | 1027.5317 | -0.0578 | -56 | 511 | 519 | SAKIPENIR |  |  |
| 1043.5481 | 1043.6091 | 0.061 | 58 | 1213 | 1221 | QVVLQDEGR |  |  |
| 1043.5481 | 1043.6091 | 0.061 | 58 | 1213 | 1221 | QVVLQDEGR | 9 |  |
| 1199.7722 | 1199.717 | -0.0552 | -46 | 213 | 223 | KLLTGVLSQIK |  |  |
| 1199.7722 | 1199.717 | -0.0552 | -46 | 213 | 223 | KLLTGVLSQIK |  |  |
| 1247.6161 | 1247.6497 | 0.0336 | 27 | 303 | 312 | MRLLNDDQSR |  |  |
| 1265.6624 | 1265.6266 | -0.0358 | -28 | 1355 | 1366 | GLVGTISEIYVD |  |  |
| 1479.7744 | 1479.7714 | -0.003 | -2 | 833 | 844 | IKQHSWTTFGFK |  |  |
| 1537.791 | 1537.7889 | -0.0021 | -1 | 786 | 797 | VDWIIFDNRGFR |  |  |
| 1562.8478 | 1562.7222 | -0.1256 | -80 | 110 | 121 | QRELFFQLAPWK |  |  |
| 1653.8806 | 1653.8394 | -0.0412 | -25 | 851 | 866 | ELSIKLGGIGAHESDK |  |  |
| 1660.8389 | 1660.8396 | 0.0007 | 0 | 1222 | 1236 | EEQQLQLVSSPSTSK |  |  |
| 1705.8214 | 1705.858 | 0.0366 | 21 | 1264 | 1277 | LELRGTEEMEHFSK | 13 |  |
| 1705.8214 | 1705.858 | 0.0366 | 21 | 1264 | 1277 | LELRGTEEMEHFSK |  |  |
| 1743.9132 | 1743.8798 | -0.0334 | -19 | 1 | 17 | MDVPVTASLGSMGPLLR |  |  |
| 1743.9132 | 1743.8798 | -0.0334 | -19 | 1 | 17 | MDVPVTASLGSMGPLLR | 53 |  |
| 1809.0204 | 1808.9845 | -0.0359 | -20 | 341 | 357 | CGGFPLAILHIASLLAR |  | Carbamidomethyl (C)[1] |
| 1814.0205 | 1813.9292 | -0.0913 | -50 | 1310 | 1325 | LTNLKILEISSCPAVR |  | Carbamidomethyl (C)[12] |
| 1814.0356 | 1813.9292 | -0.1064 | -59 | 730 | 744 | LELLPRICIFSSLPR |  | Carbamidomethyl (C)[8] |
| 1943.9547 | 1943.9963 | 0.0416 | 21 | 801 | 815 | YFKFMYTAPCLSFLR |  | Carbamidomethyl (C)[10] |

| **Gel Idx/Pos** | **1051** |  |  |  |  |  |  |  |
| --- | --- | --- | --- | --- | --- | --- | --- | --- |
| **Accession No.** | **Protein Name** | **Protein PI** | **Protein MW** | **Protein Score** | **Protein Score C. I. %** | **Total Ion Score** | **Total Ion C. I. %** | **Pep. Count** |
| gi|195627890 | stress responsive protein [Zea mays] | 6.95 | 23158.2 | 72 | 93.493 | 48 | 99.507 | 3 |
| **Peptide Information** | | | | | | | | |
| **Calc. Mass** | **Obsrv. Mass** | **± da** | **± ppm** | **Start Seq.** | **End Seq.** | **Sequence** | **Total Ion Score** | **Modification** |
| 975.3989 | 975.4641 | 0.0652 | 67 | 1 | 9 | MDGGYYGGR |  |  |
| 1064.4215 | 1064.4303 | 0.0088 | 8 | 191 | 198 | WCEGDNQR |  | Carbamidomethyl (C)[2] |
| 1064.4215 | 1064.4303 | 0.0088 | 8 | 191 | 198 | WCEGDNQR | 72 | Carbamidomethyl (C)[2] |
| 2077.9397 | 2078.0715 | 0.1318 | 63 | 182 | 198 | DGTDVVLWKWCEGDNQR |  | Carbamidomethyl (C)[11] |

| **Gel Idx/Pos** | **1281** |  |  |  |  |  |  |  |
| --- | --- | --- | --- | --- | --- | --- | --- | --- |
| **Accession No.** | **Protein Name** | **Protein PI** | **Protein MW** | **Protein Score** | **Protein Score C. I. %** | **Total Ion Score** | **Total Ion C. I. %** | **Pep. Count** |
| gi|212274863 | exoglucanase1 [Zea mays] | 6.92 | 67326.3 | 83 | 99.543 | 24 | 0 | 12 |
| **Peptide Information** | | | | | | | | |
| **Calc. Mass** | **Obsrv. Mass** | **± da** | **± ppm** | **Start Seq.** | **End Seq.** | **Sequence** | **Total Ion Score** | **Modification** |
| 814.5145 | 814.4932 | -.0213 | -26 | 38 | 44 | IKDLLGR |  |  |
| 851.4257 | 851.4325 | .0068 | 8 | 363 | 369 | IDDAVYR |  |  |
| 947.5461 | 947.5824 | .0363 | 38 | 586 | 592 | LPRTWFK |  |  |
| 1038.5439 | 1038.5348 | -.0091 | -9 | 396 | 403 | QEHRELAR |  |  |
| 1058.5841 | 1058.5829 | -.0012 | -1 | 149 | 158 | IGEATALEVR |  |  |
| 1058.5841 | 1058.5829 | -.0012 | -1 | 149 | 158 | IGEATALEVR | 74 |  |
| 1185.6879 | 1185.6801 | -.0078 | -7 | 418 | 428 | SSYAPLLPLPK |  |  |
| 1214.6852 | 1214.676 | -.0092 | -8 | 148 | 158 | RIGEATALEVR |  |  |
| 1453.791 | 1453.8011 | .0101 | 7 | 128 | 141 | ATIFPHNVGLGATR |  |  |
| 1766.8717 | 1766.8717 | 0 | 0 | 159 | 174 | ATGIPYAFAPCIAVCR |  | Carbamidomethyl (C)[11,15] |
| 1814.9371 | 1814.9242 | -.0129 | -7 | 282 | 296 | MHANHFLVTDFLKNK |  |  |
| 2135.0525 | 2135.0352 | -.0173 | -8 | 159 | 177 | ATGIPYAFAPCIAVCRDPR |  | Carbamidomethyl (C)[11,15] |
| 2299.2554 | 2299.1597 | -.0957 | -42 | 7 | 26 | ATTLVLMFCLLALGRAEYLK |  | Carbamidomethyl (C)[9], Oxidation (M)[7] |

| **Gel Idx/Pos** | **1079** |  |  |  |  |  |  |  |
| --- | --- | --- | --- | --- | --- | --- | --- | --- |
| **Accession No.** | **Protein Name** | **Protein PI** | **Protein MW** | **Protein Score** | **Protein Score C. I. %** | **Total Ion Score** | **Total Ion C. I. %** | **Pep. Count** |
| gi|195634659 | fructose-bisphosphate aldolase [Zea mays] | 7.63 | 41923.5 | 915 | 100 | 766 | 100 | 17 |
| **Peptide Information** | | | | | | | | |
| **Calc. Mass** | **Obsrv. Mass** | **± da** | **± ppm** | **Start Seq.** | **End Seq.** | **Sequence** | **Total Ion Score** | **Modification** |
| 873.4577 | 873.5159 | 0.0582 | 67 | 193 | 200 | EAAWGLAR |  |  |
| 873.4577 | 873.5159 | 0.0582 | 67 | 193 | 200 | EAAWGLAR | 65 |  |
| 901.5101 | 901.5151 | 0.005 | 6 | 359 | 367 | ANSLAQLGK |  |  |
| 947.4979 | 947.5483 | 0.0504 | 53 | 330 | 337 | ALQNTCLK |  | Carbamidomethyl (C)[6] |
| 970.5679 | 970.6136 | 0.0457 | 47 | 348 | 356 | AAQDALLLR |  |  |
| 970.5679 | 970.6136 | 0.0457 | 47 | 348 | 356 | AAQDALLLR | 72 |  |
| 1115.5845 | 1115.6208 | 0.0363 | 33 | 338 | 347 | TWGGQPDKVK |  |  |
| 1141.5007 | 1141.5554 | 0.0547 | 48 | 368 | 378 | YTSDGEAAEAK |  |  |
| 1156.5382 | 1156.5723 | 0.0341 | 29 | 163 | 172 | EAAYYQQGAR |  |  |
| 1156.5382 | 1156.5723 | 0.0341 | 29 | 163 | 172 | EAAYYQQGAR | 77 |  |
| 1291.6893 | 1291.6923 | 0.003 | 2 | 271 | 282 | ATPEQVAAYTLK |  |  |
| 1387.7175 | 1387.7222 | 0.0047 | 3 | 73 | 85 | LASIGLENTEANR |  |  |
| 1387.7175 | 1387.7222 | 0.0047 | 3 | 73 | 85 | LASIGLENTEANR | 132 |  |
| 1510.8475 | 1510.8197 | -0.0278 | -18 | 178 | 192 | TVVSIPNGPSELAVK |  |  |
| 1543.8187 | 1543.7999 | -0.0188 | -12 | 72 | 85 | RLASIGLENTEANR |  |  |
| 1562.8174 | 1562.7944 | -0.023 | -15 | 269 | 282 | DRATPEQVAAYTLK |  |  |
| 1562.8174 | 1562.7944 | -0.023 | -15 | 269 | 282 | DRATPEQVAAYTLK | 38 |  |
| 1622.7626 | 1622.731 | -0.0316 | -19 | 58 | 72 | GILAMDESNATCGKR |  | Carbamidomethyl (C)[12] |
| 1622.7626 | 1622.731 | -0.0316 | -19 | 58 | 72 | GILAMDESNATCGKR | 107 | Carbamidomethyl (C)[12] |
| 1638.7574 | 1638.7349 | -0.0225 | -14 | 58 | 72 | GILAMDESNATCGKR |  | Carbamidomethyl (C)[12], Oxidation (M)[5] |
| 1832.8372 | 1832.7981 | -0.0391 | -21 | 368 | 384 | YTSDGEAAEAKEGMFVK |  |  |
| 2401.1565 | 2401.1038 | -0.0527 | -22 | 140 | 162 | GLVPLAGSNNESWCQGLDGLASR |  | Carbamidomethyl (C)[14] |
| 2401.1565 | 2401.1038 | -0.0527 | -22 | 140 | 162 | GLVPLAGSNNESWCQGLDGLASR | 121 | Carbamidomethyl (C)[14] |
| 2743.3469 | 2743.3059 | -0.041 | -15 | 137 | 162 | VDKGLVPLAGSNNESWCQGLDGLASR |  | Carbamidomethyl (C)[17] |
| 3047.5684 | 3047.5547 | -0.0137 | -4 | 201 | 228 | YAAISQDNGLVPIVEPEILLDGEHGIER |  |  |
| 3047.5684 | 3047.5547 | -0.0137 | -4 | 201 | 228 | YAAISQDNGLVPIVEPEILLDGEHGIER | 155 |  |

| **Gel Idx/Pos** | **1177** |  |  |  |  |  |  |  |
| --- | --- | --- | --- | --- | --- | --- | --- | --- |
| **Accession No.** | **Protein Name** | **Protein PI** | **Protein MW** | **Protein Score** | **Protein Score C. I. %** | **Total Ion Score** | **Total Ion C. I. %** | **Pep. Count** |
| gi|194032833 | defensin [Zea mays] | 7.48 | 12071.7 | 82 | 91.0286 | 86 | 98 | 5 |
| **Peptide Information** | | | | | | | | |
| **Calc. Mass** | **Obsrv. Mass** | **± da** | **± ppm** | **Start Seq.** | **End Seq.** | **Sequence** | **Total Ion Score** | **Modification** |
| 1013.439 | 1013.534 | 0.095 | 94 | 29 | 36 | LCSTTMER |  | Carbamidomethyl (C)[2], Oxidation (M)[6] |
| 1406.6191 | 1406.6321 | 0.013 | 9 | 57 | 69 | SKGYTGGGFCNMK |  | Carbamidomethyl (C)[10] |
| 1589.72 | 1589.6578 | -0.0622 | -39 | 59 | 72 | GYTGGGFCNMKIER |  | Carbamidomethyl (C)[8] |
| 1646.7183 | 1646.6626 | -0.0557 | -34 | 23 | 36 | MDATAKLCSTTMER |  | Carbamidomethyl (C)[8], Oxidation (M)[1,12] |
| 1674.5928 | 1674.6914 | 0.0986 | 59 | 91 | 106 | DEAADAGDMMSQTMAD |  | Oxidation (M)[9] |
| 1690.5878 | 1690.6575 | 0.0697 | 41 | 91 | 106 | DEAADAGDMMSQTMAD | 69 | Oxidation (M)[9,10] |

| **Gel Idx/Pos** | **991** |  |  |  |  |  |  |  |
| --- | --- | --- | --- | --- | --- | --- | --- | --- |
| **Accession No.** | **Protein Name** | **Protein PI** | **Protein MW** | **Protein Score** | **Protein Score C. I. %** | **Total Ion Score** | **Total Ion C. I. %** | **Pep. Count** |
| gi|195612198 | fructose-bisphosphate aldolase, cytoplasmic isozyme 1[Zea mays] | 6.26 | 38464 | 683 | 100 | 552 | 100 | 15 |
| **Peptide Information** | | | | | | | | |
| **Calc. Mass** | **Obsrv. Mass** | **± da** | **± ppm** | **Start Seq.** | **End Seq.** | **Sequence** | **Total Ion Score** | **Modification** |
| 829.3839 | 829.416 | 0.0321 | 39 | 133 | 139 | YYEAGAR |  |  |
| 829.3839 | 829.416 | 0.0321 | 39 | 133 | 139 | YYEAGAR | 50 |  |
| 881.4614 | 881.4778 | 0.0164 | 19 | 8 | 14 | YTDELIK |  |  |
| 886.4741 | 886.4998 | 0.0257 | 29 | 164 | 171 | QNVEGLAR |  |  |
| 886.4741 | 886.4998 | 0.0257 | 29 | 164 | 171 | QNVEGLAR | 52 |  |
| 999.6448 | 999.6337 | -0.0111 | -11 | 220 | 228 | VLLEATLLK |  |  |
| 1465.7759 | 1465.771 | -0.0049 | -3 | 149 | 163 | VGPAGQGQPSELAVR |  |  |
| 1465.7759 | 1465.771 | -0.0049 | -3 | 149 | 163 | VGPAGQGQPSELAVR | 126 |  |
| 1474.7496 | 1474.7439 | -0.0057 | -4 | 40 | 52 | LSSINLENVESNR |  |  |
| 1474.7496 | 1474.7439 | -0.0057 | -4 | 40 | 52 | LSSINLENVESNR | 23 |  |
| 1591.869 | 1591.8608 | -0.0082 | -5 | 241 | 255 | VSAEVIAEYTVAALR |  |  |
| 1591.869 | 1591.8608 | -0.0082 | -5 | 241 | 255 | VSAEVIAEYTVAALR | 99 |  |
| 1630.8507 | 1630.8442 | -0.0065 | -4 | 39 | 52 | RLSSINLENVESNR |  |  |
| 1666.9739 | 1666.9324 | -0.0415 | -25 | 214 | 228 | SLSDHKVLLEATLLK |  |  |
| 1747.9701 | 1747.9658 | -0.0043 | -2 | 241 | 256 | VSAEVIAEYTVAALRR |  |  |
| 1747.9701 | 1747.9658 | -0.0043 | -2 | 241 | 256 | VSAEVIAEYTVAALRR | 57 |  |
| 1877.0604 | 1877.0406 | -0.0198 | -11 | 145 | 163 | AVLKVGPAGQGQPSELAVR |  |  |
| 2069.1643 | 2069.1401 | -0.0242 | -12 | 83 | 103 | TSDGKPFVDVLIAGGVVPGIK |  |  |
| 2249.0894 | 2249.1025 | 0.0131 | 6 | 107 | 129 | GTVEIAGTDGETTTQGLDSLGAR |  |  |
| 2249.0894 | 2249.1025 | 0.0131 | 6 | 107 | 129 | GTVEIAGTDGETTTQGLDSLGAR | 147 |  |
| 2591.2795 | 2591.3081 | 0.0286 | 11 | 104 | 129 | VDKGTVEIAGTDGETTTQGLDSLGAR |  |  |
| 3782.8364 | 3782.9565 | 0.1201 | 32 | 172 | 206 | YALICQENGLVPIVEPEILTDGGHDIGTCAAATER |  | Carbamidomethyl (C)[5,29] |

| **Gel Idx/Pos** | **778** |  |  |  |  |  |  |  |
| --- | --- | --- | --- | --- | --- | --- | --- | --- |
| **Accession No.** | **Protein Name** | **Protein PI** | **Protein MW** | **Protein Score** | **Protein Score C. I. %** | **Total Ion Score** | **Total Ion C. I. %** | **Pep. Count** |
| gi|224028705 | unknown [Zea mays] | 8.35 | 51877.9 | 511 | 100 | 334 | 100 | 21 |
| **Peptide Information** | | | | | | | | |
| **Calc. Mass** | **Obsrv. Mass** | **± da** | **± ppm** | **Start Seq.** | **End Seq.** | **Sequence** | **Total Ion Score** | **Modification** |
| 814.5145 | 814.5612 | 0.0467 | 57 | 98 | 104 | IAQTLLR |  |  |
| 814.5145 | 814.5612 | 0.0467 | 57 | 98 | 104 | IAQTLLR | 19 |  |
| 852.4978 | 852.5011 | 0.0033 | 4 | 480 | 487 | ALFGGLFK |  |  |
| 856.4886 | 856.5352 | 0.0466 | 54 | 131 | 138 | LISPAEAR |  |  |
| 856.4886 | 856.5352 | 0.0466 | 54 | 131 | 138 | LISPAEAR | 18 |  |
| 1012.5897 | 1012.6086 | 0.0189 | 19 | 131 | 139 | LISPAEARR |  |  |
| 1065.5687 | 1065.603 | 0.0343 | 32 | 471 | 479 | QPDPKPEVR |  |  |
| 1181.5837 | 1181.5775 | -0.0062 | -5 | 409 | 418 | VISADFSWEK |  |  |
| 1264.7008 | 1264.7273 | 0.0265 | 21 | 469 | 479 | AKQPDPKPEVR |  |  |
| 1264.7008 | 1264.7273 | 0.0265 | 21 | 469 | 479 | AKQPDPKPEVR | 14 |  |
| 1318.6598 | 1318.6707 | 0.0109 | 8 | 281 | 293 | EGVSPSDTGKVSR |  |  |
| 1333.623 | 1333.6528 | 0.0298 | 22 | 351 | 362 | AQAEEETLASER |  |  |
| 1333.623 | 1333.6528 | 0.0298 | 22 | 351 | 362 | AQAEEETLASER | 45 |  |
| 1344.639 | 1344.6831 | 0.0441 | 33 | 173 | 186 | GPEGGGVTTDDALR |  |  |
| 1393.7686 | 1393.7714 | 0.0028 | 2 | 249 | 260 | VAETDVRYTLVK |  |  |
| 1397.7383 | 1397.7592 | 0.0209 | 15 | 111 | 124 | AGVPDLASAQELAR |  |  |
| 1397.7383 | 1397.7592 | 0.0209 | 15 | 111 | 124 | AGVPDLASAQELAR | 83 |  |
| 1446.7224 | 1446.75 | 0.0276 | 19 | 327 | 339 | NIAEAFTAIPEDR |  |  |
| 1446.7224 | 1446.75 | 0.0276 | 19 | 327 | 339 | NIAEAFTAIPEDR | 63 |  |
| 1587.8014 | 1587.7997 | -0.0017 | -1 | 140 | 155 | LNAVEAGFGDPDAIAK |  |  |
| 1602.8235 | 1602.8165 | -0.007 | -4 | 327 | 340 | NIAEAFTAIPEDRR |  |  |
| 1646.798 | 1646.8229 | 0.0249 | 15 | 351 | 365 | AQAEEETLASERANK |  |  |
| 1673.8606 | 1673.8466 | -0.014 | -8 | 81 | 97 | DPQTVFVAGATGQAGVR |  |  |
| 1735.9126 | 1735.9475 | 0.0349 | 20 | 61 | 77 | SLIQAFPSQSLFAGGGR |  |  |
| 1743.9025 | 1743.9248 | 0.0223 | 13 | 139 | 155 | RLNAVEAGFGDPDAIAK |  |  |
| 1743.9025 | 1743.9248 | 0.0223 | 13 | 139 | 155 | RLNAVEAGFGDPDAIAK | 7 |  |
| 1801.9556 | 1801.9645 | 0.0089 | 5 | 80 | 97 | KDPQTVFVAGATGQAGVR |  |  |
| 2314.1885 | 2314.2466 | 0.0581 | 25 | 163 | 186 | VVVTVGSAEKGPEGGGVTTDDALR |  |  |
| 2314.1885 | 2314.2466 | 0.0581 | 25 | 163 | 186 | VVVTVGSAEKGPEGGGVTTDDALR | 85 |  |

| **Gel Idx/Pos** | **1955** |  |  |  |  |  |  |  |
| --- | --- | --- | --- | --- | --- | --- | --- | --- |
| **Accession No.** | **Protein Name** | **Protein PI** | **Protein MW** | **Protein Score** | **Protein Score C. I. %** | **Total Ion Score** | **Total Ion C. I. %** | **Pep. Count** |
| gi|195637254 | nucleoside diphosphate kinase 2 [Zea mays] | 9.51 | 24181.7 | 355 | 100 | 250 | 100 | 12 |
| **Peptide Information** | | | | | | | | |
| **Calc. Mass** | **Obsrv. Mass** | **± da** | **± ppm** | **Start Seq.** | **End Seq.** | **Sequence** | **Total Ion Score** | **Modification** |
| 878.4771 | 878.5054 | 0.0283 | 32 | 127 | 133 | DKPFFPK |  |  |
| 878.4771 | 878.5054 | 0.0283 | 32 | 127 | 133 | DKPFFPK | 10 |  |
| 916.4847 | 916.5133 | 0.0286 | 31 | 176 | 184 | GDLAVQTGR |  |  |
| 916.4847 | 916.5133 | 0.0286 | 31 | 176 | 184 | GDLAVQTGR | 58 |  |
| 943.5571 | 943.5764 | 0.0193 | 20 | 89 | 97 | GLVGEIISR |  |  |
| 964.5138 | 964.5283 | 0.0145 | 15 | 199 | 205 | EIELWFK |  |  |
| 1003.4843 | 1003.5102 | 0.0259 | 26 | 116 | 123 | DLAQEHYK |  |  |
| 1359.6903 | 1359.7103 | 0.02 | 15 | 116 | 126 | DLAQEHYKDLK |  |  |
| 1406.7461 | 1406.7496 | 0.0035 | 2 | 77 | 88 | SYIMIKPDGVQR |  |  |
| 1406.7461 | 1406.7496 | 0.0035 | 2 | 77 | 88 | SYIMIKPDGVQR | 17 |  |
| 1422.741 | 1422.7291 | -0.0119 | -8 | 77 | 88 | SYIMIKPDGVQR |  | Oxidation (M)[4] |
| 1422.741 | 1422.7291 | -0.0119 | -8 | 77 | 88 | SYIMIKPDGVQR | 23 | Oxidation (M)[4] |
| 1481.7092 | 1481.6986 | -0.0106 | -7 | 185 | 198 | NVVHGSDSPDNGKR |  |  |
| 1481.7092 | 1481.6986 | -0.0106 | -7 | 185 | 198 | NVVHGSDSPDNGKR | 21 |  |
| 1540.8442 | 1540.7897 | -0.0545 | -35 | 63 | 76 | AVPQRIVASSEVER |  |  |
| 1650.9174 | 1650.916 | -0.0014 | -1 | 160 | 175 | LIGATNPLQAEPGTIR |  |  |
| 1650.9174 | 1650.916 | -0.0014 | -1 | 160 | 175 | LIGATNPLQAEPGTIR | 66 |  |
| 1779.0123 | 1779.0095 | -0.0028 | -2 | 159 | 175 | KLIGATNPLQAEPGTIR |  |  |
| 1779.0123 | 1779.0095 | -0.0028 | -2 | 159 | 175 | KLIGATNPLQAEPGTIR | 71 |  |
| 2548.3843 | 2548.3459 | -0.0384 | -15 | 160 | 184 | LIGATNPLQAEPGTIRGDLAVQTGR |  |  |
| 2548.3843 | 2548.3459 | -0.0384 | -15 | 160 | 184 | LIGATNPLQAEPGTIRGDLAVQTGR | 2 |  |
| 2548.3843 | 2548.5947 | 0.2104 | 83 | 160 | 184 | LIGATNPLQAEPGTIRGDLAVQTGR |  |  |

| **Gel Idx/Pos** | **1771** |  |  |  |  |  |  |  |
| --- | --- | --- | --- | --- | --- | --- | --- | --- |
| **Accession No.** | **Protein Name** | **Protein PI** | **Protein MW** | **Protein Score** | **Protein Score C. I. %** | **Total Ion Score** | **Total Ion C. I. %** | **Pep. Count** |
| gi|194705220 | unknown [Zea mays] | 9.88 | 10854.7 | 396 | 100 | 278 | 100 | 12 |
| **Peptide Information** | | | | | | | | |
| **Calc. Mass** | **Obsrv. Mass** | **± da** | **± ppm** | **Start Seq.** | **End Seq.** | **Sequence** | **Total Ion Score** | **Modification** |
| 807.3883 | 807.3831 | -.0052 | -6 | 57 | 63 | DGVYPEK |  |  |
| 898.4993 | 898.4892 | -.0101 | -11 | 81 | 88 | NVNPIDVK |  |  |
| 906.4428 | 906.4462 | .0034 | 4 | 69 | 76 | EGVGQNFR |  |  |
| 990.5043 | 990.5165 | .0122 | 12 | 37 | 43 | ITYQFYR |  |  |
| 990.5043 | 990.5165 | .0122 | 12 | 37 | 43 | ITYQFYR | 44 |  |
| 1047.5251 | 1047.5361 | .011 | 11 | 22 | 30 | EQCLALGTR |  | Carbamidomethyl (C)[3] |
| 1110.663 | 1110.6652 | .0022 | 2 | 11 | 20 | EGPNLLKLAR |  |  |
| 1175.6201 | 1175.6317 | .0116 | 10 | 21 | 30 | KEQCLALGTR |  | Carbamidomethyl (C)[4] |
| 1175.6201 | 1175.6317 | .0116 | 10 | 21 | 30 | KEQCLALGTR | 76 | Carbamidomethyl (C)[4] |
| 1281.6626 | 1281.6917 | .0291 | 23 | 35 | 43 | YKITYQFYR |  |  |
| 1283.7318 | 1283.7435 | .0117 | 9 | 77 | 88 | SIGKNVNPIDVK |  |  |
| 1330.675 | 1330.7021 | .0271 | 20 | 57 | 68 | DGVYPEKVNPGR |  |  |
| 1330.675 | 1330.7021 | .0271 | 20 | 57 | 68 | DGVYPEKVNPGR | 72 |  |
| 1429.7295 | 1429.7629 | .0334 | 23 | 64 | 76 | VNPGREGVGQNFR |  |  |
| 1429.7295 | 1429.7629 | .0334 | 23 | 64 | 76 | VNPGREGVGQNFR | 22 |  |
| 1527.7955 | 1527.8333 | .0378 | 25 | 44 | 56 | VFPNGEVQYLHPK |  |  |
| 1527.7955 | 1527.8333 | .0378 | 25 | 44 | 56 | VFPNGEVQYLHPK | 64 |  |

| **Gel Idx/Pos** | **1735** |  |  |  |  |  |  |  |
| --- | --- | --- | --- | --- | --- | --- | --- | --- |
| **Accession No.** | **Protein Name** | **Protein PI** | **Protein MW** | **Protein Score** | **Protein Score C. I. %** | **Total Ion Score** | **Total Ion C. I. %** | **Pep. Count** |
| gi|226500452 | protochlorophyllide reductase B [Zea mays] | 8.96 | 42434.9 | 203 | 100 | 173 | 100 | 7 |
| **Peptide Information** | | | | | | | | |
| **Calc. Mass** | **Obsrv. Mass** | **± da** | **± ppm** | **Start Seq.** | **End Seq.** | **Sequence** | **Total Ion Score** | **Modification** |
| 836.4625 | 836.4272 | -.0353 | -42 | 117 | 123 | DFLKASR |  |  |
| 911.5098 | 911.5084 | -.0014 | -2 | 313 | 319 | EHIPLFR |  |  |
| 911.5098 | 911.5084 | -.0014 | -2 | 313 | 319 | EHIPLFR | 38 |  |
| 989.5818 | 989.5667 | -.0151 | -15 | 320 | 327 | LLFPPFQK |  |  |
| 1224.5856 | 1224.5873 | .0017 | 1 | 332 | 342 | GYVSEEEAGKR |  |  |
| 1224.5856 | 1224.5873 | .0017 | 1 | 332 | 342 | GYVSEEEAGKR | 51 |  |
| 1737.8654 | 1737.8744 | .009 | 5 | 203 | 217 | ELLSDLQSSDYPSKR |  |  |
| 1737.8654 | 1737.8744 | .009 | 5 | 203 | 217 | ELLSDLQSSDYPSKR | 86 |  |
| 1895.8561 | 1895.8534 | -.0027 | -1 | 273 | 287 | DSKVCNMLTMQEFHR |  | Carbamidomethyl (C)[5] |
| 2140.9629 | 2140.9697 | .0068 | 3 | 364 | 383 | NSASFENQLSEEASDADKAK |  |  |

| **Gel Idx/Pos** | **984** |  |  |  |  |  |  |  |
| --- | --- | --- | --- | --- | --- | --- | --- | --- |
| **Accession No.** | **Protein Name** | **Protein PI** | **Protein MW** | **Protein Score** | **Protein Score C. I. %** | **Total Ion Score** | **Total Ion C. I. %** | **Pep. Count** |
| gi|162464489 | glutamate dehydrogenase [Zea mays] | 6.09 | 44279.8 | 172 | 100 | 106 | 100 | 11 |
| **Peptide Information** | | | | | | | | |
| **Calc. Mass** | **Obsrv. Mass** | **± da** | **± ppm** | **Start Seq.** | **End Seq.** | **Sequence** | **Total Ion Score** | **Modification** |
| 806.4519 | 806.4393 | -0.0126 | -16 | 10 | 16 | NFKQAAK |  |  |
| 845.5243 | 845.5512 | 0.0269 | 32 | 27 | 33 | SLLIPFR |  |  |
| 845.5243 | 845.5512 | 0.0269 | 32 | 27 | 33 | SLLIPFR | 2 |  |
| 874.3835 | 874.4607 | 0.0772 | 88 | 383 | 389 | SHSCDLR |  | Carbamidomethyl (C)[4] |
| 1002.5367 | 1002.559 | 0.0223 | 22 | 403 | 411 | ATVLRGWEA |  |  |
| 1002.5367 | 1002.559 | 0.0223 | 22 | 403 | 411 | ATVLRGWEA | 22 |  |
| 1065.551 | 1065.5756 | 0.0246 | 23 | 390 | 399 | MGAFTLGVNR |  |  |
| 1172.6886 | 1172.6465 | -0.0421 | -36 | 232 | 243 | VIAISDVTGAVK |  |  |
| 1215.746 | 1215.73 | -0.016 | -13 | 27 | 36 | SLLIPFREIK |  |  |
| 1300.6168 | 1300.5966 | -0.0202 | -16 | 44 | 55 | DDGTLASYVGFR |  |  |
| 1300.6168 | 1300.5966 | -0.0202 | -16 | 44 | 55 | DDGTLASYVGFR | 42 |  |
| 1465.6094 | 1465.7321 | 0.1227 | 84 | 379 | 389 | QMCRSHSCDLR |  | Carbamidomethyl (C)[3,8], Oxidation (M)[2] |
| 1465.6094 | 1465.7321 | 0.1227 | 84 | 379 | 389 | QMCRSHSCDLR |  | Carbamidomethyl (C)[3,8], Oxidation (M)[2] |
| 1746.8328 | 1746.7573 | -0.0755 | -43 | 103 | 119 | GGIGCSPGDLSISELER |  | Carbamidomethyl (C)[5] |
| 1746.8328 | 1746.7573 | -0.0755 | -43 | 103 | 119 | GGIGCSPGDLSISELER | 40 | Carbamidomethyl (C)[5] |
| 2226.0925 | 2225.8921 | -0.2004 | -90 | 306 | 325 | YIIEAANHPTDPEADEILSK |  |  |

| **Gel Idx/Pos** | **1134** |  |  |  |  |  |  |  |
| --- | --- | --- | --- | --- | --- | --- | --- | --- |
| **Accession No.** | **Protein Name** | **Protein PI** | **Protein MW** | **Protein Score** | **Protein Score C. I. %** | **Total Ion Score** | **Total Ion C. I. %** | **Pep. Count** |
| gi|195633817 | oxygen-evolving enhancer protein 3-1 [Zea mays] | 9.77 | 23104.5 | 267 | 100 | 209 | 100 | 8 |
| **Peptide Information** | | | | | | | | |
| **Calc. Mass** | **Obsrv. Mass** | **± da** | **± ppm** | **Start Seq.** | **End Seq.** | **Sequence** | **Total Ion Score** | **Modification** |
| 1128.6047 | 1128.6049 | 0.0002 | 0 | 150 | 158 | ASYLRYDLK |  |  |
| 1132.5997 | 1132.5946 | -0.0051 | -5 | 97 | 105 | DFDLPLKER |  |  |
| 1132.5997 | 1132.5946 | -0.0051 | -5 | 97 | 105 | DFDLPLKER | 40 |  |
| 1261.6324 | 1261.6335 | 0.0011 | 1 | 138 | 147 | AWPYVQNDLR |  |  |
| 1261.6324 | 1261.6335 | 0.0011 | 1 | 138 | 147 | AWPYVQNDLR | 16 |  |
| 1442.7162 | 1442.7117 | -0.0045 | -3 | 194 | 206 | STPEAEKYFAATK |  |  |
| 1445.7272 | 1445.726 | -0.0012 | -1 | 179 | 191 | LFSTIDDLDHAAK |  |  |
| 1445.7272 | 1445.726 | -0.0012 | -1 | 179 | 191 | LFSTIDDLDHAAK |  |  |
| 1543.8268 | 1543.8317 | 0.0049 | 3 | 106 | 119 | FYLQPLPPAEAAAR |  |  |
| 1543.8268 | 1543.8317 | 0.0049 | 3 | 106 | 119 | FYLQPLPPAEAAAR |  |  |
| 1582.8475 | 1582.7803 | -0.0672 | -42 | 201 | 215 | YFAATKDALGDVLAK |  |  |
| 2002.0353 | 2002.0341 | -0.0012 | -1 | 76 | 96 | IGAPPPPSGGLPGTLNSDQAR |  |  |
| 2002.0353 | 2002.0341 | -0.0012 | -1 | 76 | 96 | IGAPPPPSGGLPGTLNSDQAR |  |  |

| **Gel Idx/Pos** | **1716** |  |  |  |  |  |  |  |
| --- | --- | --- | --- | --- | --- | --- | --- | --- |
| **Accession No.** | **Protein Name** | **Protein PI** | **Protein MW** | **Protein Score** | **Protein Score C. I. %** | **Total Ion Score** | **Total Ion C. I. %** | **Pep. Count** |
| gi|149392465 | ferredoxin-nadp reductase, leaf isozyme [Oryza sativa Indica Group] | 6.59 | 21768.9 | 139 | 100 | 109 | 100 | 6 |
| **Peptide Information** | | | | | | | | |
| **Calc. Mass** | **Obsrv. Mass** | **± da** | **± ppm** | **Start Seq.** | **End Seq.** | **Sequence** | **Total Ion Score** | **Modification** |
| 809.4152 | 809.3911 | -0.0241 | -30 | 103 | 109 | VDYAVSR |  |  |
| 811.413 | 811.3856 | -0.0274 | -34 | 119 | 124 | MYIQTR |  |  |
| 811.413 | 811.3856 | -0.0274 | -34 | 119 | 124 | MYIQTR | 20 |  |
| 827.408 | 827.3798 | -0.0282 | -34 | 119 | 124 | MYIQTR |  | Oxidation (M)[1] |
| 1232.6555 | 1232.6367 | -0.0188 | -15 | 154 | 165 | GIDDIMVSLAAK |  |  |
| 1363.6022 | 1363.6902 | 0.088 | 65 | 139 | 149 | DNTYVYMCGLK |  | Carbamidomethyl (C)[8] |
| 1507.692 | 1507.7798 | 0.0878 | 58 | 138 | 149 | KDNTYVYMCGLK |  | Carbamidomethyl (C)[9], Oxidation (M)[8] |
| 1523.7601 | 1523.8147 | 0.0546 | 36 | 97 | 109 | APENFRVDYAVSR |  |  |
| 1523.7601 | 1523.8147 | 0.0546 | 36 | 97 | 109 | APENFRVDYAVSR | 89 |  |

| **Gel Idx/Pos** | **1571** |  |  |  |  |  |  |  |
| --- | --- | --- | --- | --- | --- | --- | --- | --- |
| **Accession No.** | **Protein Name** | **Protein PI** | **Protein MW** | **Protein Score** | **Protein Score C. I. %** | **Total Ion Score** | **Total Ion C. I. %** | **Pep. Count** |
| gi|195639710 | ubiquitin fusion protein [Zea mays] | 9.94 | 14977.1 | 70 | 70 | 21 | 0 | 3 |
| **Peptide Information** | | | | | | | | |
| **Calc. Mass** | **Obsrv. Mass** | **± da** | **± ppm** | **Start Seq.** | **End Seq.** | **Sequence** | **Total Ion Score** | **Modification** |
| 1039.5167 | 1039.5134 | -0.0033 | -3 | 34 | 42 | EGIPPDQQR |  |  |
| 1072.5469 | 1072.5007 | -0.0462 | -43 | 99 | 106 | CYARLHPR |  | Carbamidomethyl (C)[1] |
| 1523.7812 | 1523.7993 | 0.0181 | 12 | 30 | 42 | IQDKEGIPPDQQR |  |  |
| 1523.7812 | 1523.7993 | 0.0181 | 12 | 30 | 42 | IQDKEGIPPDQQR | 61 |  |

| **Gel Idx/Pos** | **1337** |  |  |  |  |  |  |  |
| --- | --- | --- | --- | --- | --- | --- | --- | --- |
| **Accession No.** | **Protein Name** | **Protein PI** | **Protein MW** | **Protein Score** | **Protein Score C. I. %** | **Total Ion Score** | **Total Ion C. I. %** | **Pep. Count** |
| gi|195628632 | remorin [Zea mays] | 5.74 | 21875.4 | 111 | 100 | 56 | 99.923 | 9 |
| **Peptide Information** | | | | | | | | |
| **Calc. Mass** | **Obsrv. Mass** | **± da** | **± ppm** | **Start Seq.** | **End Seq.** | **Sequence** | **Total Ion Score** | **Modification** |
| 803.4509 | 803.4802 | 0.0293 | 36 | 9 | 15 | VEVEVTK |  |  |
| 804.4396 | 804.4869 | 0.0473 | 59 | 163 | 169 | AMVEAKR |  |  |
| 809.4628 | 809.4567 | -0.0061 | -8 | 185 | 192 | ATGHAPKK |  |  |
| 865.46 | 865.4811 | 0.0211 | 24 | 192 | 199 | KLIGCFGA |  | Carbamidomethyl (C)[5] |
| 905.3999 | 905.4825 | 0.0826 | 91 | 94 | 100 | AWEENEK |  |  |
| 935.4502 | 935.5065 | 0.0563 | 60 | 1 | 8 | MAEEEAKK |  |  |
| 1372.6927 | 1372.6952 | 0.0025 | 2 | 70 | 82 | QGGSNDRDLALAR |  |  |
| 1372.6927 | 1372.6952 | 0.0025 | 2 | 70 | 82 | QGGSNDRDLALAR | 27 |  |
| 1419.7478 | 1419.7352 | -0.0126 | -9 | 56 | 69 | VADEPAPEKPAPAK |  |  |
| 2144.196 | 2144.1516 | -0.0444 | -21 | 49 | 69 | ALAIVEKVADEPAPEKPAPAK |  |  |
| 2144.196 | 2144.1516 | -0.0444 | -21 | 49 | 69 | ALAIVEKVADEPAPEKPAPAK | 29 |  |

| **Gel Idx/Pos** | **1691** |  |  |  |  |  |  |  |
| --- | --- | --- | --- | --- | --- | --- | --- | --- |
| **Accession No.** | **Protein Name** | **Protein PI** | **Protein MW** | **Protein Score** | **Protein Score C. I. %** | **Total Ion Score** | **Total Ion C. I. %** | **Pep. Count** |
| gi|226507468 | bZIP transcription factor ABI5 [Zea mays] | 9.56 | 38416.8 | 97 | 100 | 110 | 98.856 | 9 |
| **Peptide Information** | | | | | | | | |
| **Calc. Mass** | **Obsrv. Mass** | **± da** | **± ppm** | **Start Seq.** | **End Seq.** | **Sequence** | **Total Ion Score** | **Modification** |
| 800.4335 | 800.4249 | -0.0086 | -11 | 265 | 271 | KPPAMEK |  |  |
| 800.4335 | 800.4249 | -0.0086 | -11 | 265 | 271 | KPPAMEK | 56 |  |
| 803.4118 | 803.3931 | -0.0187 | -23 | 283 | 289 | NRESAAR |  |  |
| 816.4283 | 816.3993 | -0.029 | -36 | 265 | 271 | KPPAMEK |  | Oxidation (M)[5] |
| 856.4999 | 856.5401 | 0.0402 | 47 | 337 | 344 | QVGPTAKR |  |  |
| 856.4999 | 856.5401 | 0.0402 | 47 | 336 | 343 | RQVGPTAK |  |  |
| 876.4495 | 876.4095 | -0.04 | -46 | 319 | 325 | QVEMLEK |  |  |
| 904.4523 | 904.4543 | 0.002 | 2 | 114 | 120 | TVDEVWR | 38 |  |
| 904.4523 | 904.4543 | 0.002 | 2 | 114 | 120 | TVDEVWR |  | Oxidation (M)[1] |
| 1046.5411 | 1046.5382 | -0.0029 | -3 | 328 | 335 | NEVLERMR |  |  |
| 1107.4888 | 1107.5284 | 0.0396 | 36 | 1 | 11 | MDFPGPAGSGR |  |  |

| **Gel Idx/Pos** | **687** |  |  |  |  |  |  |  |
| --- | --- | --- | --- | --- | --- | --- | --- | --- |
| **Accession No.** | **Protein Name** | **Protein PI** | **Protein MW** | **Protein Score** | **Protein Score C. I. %** | **Total Ion Score** | **Total Ion C. I. %** | **Pep. Count** |
| gi|195622012 | membrane-associated 30 kDa protein [Zea mays] | 9.5 | 35059.3 | 270 | 100 | 271 | 100 | 9 |
| **Peptide Information** | | | | | | | | |
| **Calc. Mass** | **Obsrv. Mass** | **± da** | **± ppm** | **Start Seq.** | **End Seq.** | **Sequence** | **Total Ion Score** | **Modification** |
| 1144.6321 | 1144.6002 | -0.0319 | -28 | 101 | 111 | QATAQVLASQK |  |  |
| 1201.6536 | 1201.6686 | 0.015 | 12 | 169 | 179 | GVVENLISNTR |  |  |
| 1201.6536 | 1201.6686 | 0.015 | 12 | 169 | 179 | GVVENLISNTR | 50 |  |
| 1211.6014 | 1211.5469 | -0.0545 | -45 | 151 | 161 | KSYADNASSLR |  |  |
| 1211.6014 | 1211.6566 | 0.0552 | 46 | 151 | 161 | KSYADNASSLR |  |  |
| 1267.5702 | 1267.5934 | 0.0232 | 18 | 119 | 129 | AAEQASADWYR |  |  |
| 1267.5702 | 1267.5934 | 0.0232 | 18 | 119 | 129 | AAEQASADWYR | 76 |  |
| 1300.7332 | 1300.6873 | -0.0459 | -35 | 101 | 112 | QATAQVLASQKR |  |  |
| 1423.6713 | 1423.7025 | 0.0312 | 22 | 119 | 130 | AAEQASADWYRR |  |  |
| 1423.6713 | 1423.7025 | 0.0312 | 22 | 119 | 130 | AAEQASADWYRR | 42 |  |
| 1527.8126 | 1527.8512 | 0.0386 | 25 | 131 | 144 | AQLALQKGDEDLAR |  |  |
| 1527.8126 | 1527.8512 | 0.0386 | 25 | 131 | 144 | AQLALQKGDEDLAR | 40 |  |
| 1717.8677 | 1717.9612 | 0.0935 | 54 | 84 | 98 | ILDQAVLEMNDDLTK |  |  |
| 2029.0673 | 2029.0879 | 0.0206 | 10 | 162 | 179 | SQLDQQKGVVENLISNTR |  |  |
| 2029.0673 | 2029.0879 | 0.0206 | 10 | 162 | 179 | SQLDQQKGVVENLISNTR | 62 |  |

| **Gel Idx/Pos** | **735** |  |  |  |  |  |  |  |
| --- | --- | --- | --- | --- | --- | --- | --- | --- |
| **Accession No.** | **Protein Name** | **Protein PI** | **Protein MW** | **Protein Score** | **Protein Score C. I. %** | **Total Ion Score** | **Total Ion C. I. %** | **Pep. Count** |
| gi|20257673 | glycine-rich RNA binding protein [Zea mays] | 6.89 | 14201.5 | 85 | 99.743 | 26 | 0 | 7 |
| **Peptide Information** | | | | | | | | |
| **Calc. Mass** | **Obsrv. Mass** | **± da** | **± ppm** | **Start Seq.** | **End Seq.** | **Sequence** | **Total Ion Score** | **Modification** |
| 851.3755 | 851.3937 | 0.0182 | 21 | 85 | 95 | GGGGGGYGGGR |  |  |
| 906.4349 | 906.5029 | 0.068 | 75 | 58 | 66 | SAIEGMNGK |  |  |
| 1007.4766 | 1007.4748 | -0.0018 | -2 | 85 | 96 | GGGGGGYGGGRR |  |  |
| 1131.5753 | 1131.578 | 0.0027 | 2 | 72 | 81 | NITVNEAQSR |  |  |
| 1143.6481 | 1143.6469 | -0.0012 | -1 | 33 | 41 | IILDRETQR |  |  |
| 1143.6481 | 1143.6469 | -0.0012 | -1 | 33 | 41 | IILDRETQR | 26 |  |
| 1380.5676 | 1380.6166 | 0.049 | 35 | 127 | 139 | GGGYGNNDGNWRN |  |  |
| 1564.7101 | 1564.7334 | 0.0233 | 15 | 44 | 57 | GFGFVTFSTEDAMR |  |  |
| 1580.7051 | 1580.7456 | 0.0405 | 26 | 44 | 57 | GFGFVTFSTEDAMR | 61 | Oxidation (M)[13] |

| **Gel Idx/Pos** | **1606** |  |  |  |  |  |  |  |
| --- | --- | --- | --- | --- | --- | --- | --- | --- |
| **Accession No.** | **Protein Name** | **Protein PI** | **Protein MW** | **Protein Score** | **Protein Score C. I. %** | **Total Ion Score** | **Total Ion C. I. %** | **Pep. Count** |
| gi|11467200 | ribulose-1,5-bisphosphate carboxylase/oxygenase large subunit [Zea mays] | 6.33 | 53294.6 | 131 | 100 | 103 | 100 | 7 |
| **Peptide Information** | | | | | | | | |
| **Calc. Mass** | **Obsrv. Mass** | **± da** | **± ppm** | **Start Seq.** | **End Seq.** | **Sequence** | **Total Ion Score** | **Modification** |
| 985.5789 | 985.5878 | 0.0089 | 9 | 132 | 139 | ALRLEDLR |  |  |
| 985.5789 | 985.5878 | 0.0089 | 9 | 132 | 139 | ALRLEDLR | 11 |  |
| 1021.5312 | 1021.5453 | 0.0141 | 14 | 33 | 41 | DTDILAAFR |  |  |
| 1021.5312 | 1021.5453 | 0.0141 | 14 | 33 | 41 | DTDILAAFR | 57 |  |
| 1116.583 | 1116.594 | 0.011 | 10 | 422 | 431 | VALEACVQAR |  | Carbamidomethyl (C)[6] |
| 1116.583 | 1116.594 | 0.011 | 10 | 422 | 431 | VALEACVQAR | 35 | Carbamidomethyl (C)[6] |
| 1168.6256 | 1168.5607 | -0.0649 | -56 | 296 | 305 | AMHAVIDRQK |  |  |
| 1168.6256 | 1168.5607 | -0.0649 | -56 | 296 | 305 | AMHAVIDRQK |  |  |
| 1232.6304 | 1232.5981 | -0.0323 | -26 | 440 | 450 | EGNEIIKAACK |  | Carbamidomethyl (C)[10] |
| 1465.7546 | 1465.7864 | 0.0318 | 22 | 147 | 159 | TFQGPPHGIQVER |  |  |

| **Gel Idx/Pos** | **979** |  |  |  |  |  |  |  |
| --- | --- | --- | --- | --- | --- | --- | --- | --- |
| **Accession No.** | **Protein Name** | **Protein PI** | **Protein MW** | **Protein Score** | **Protein Score C. I. %** | **Total Ion Score** | **Total Ion C. I. %** | **Pep. Count** |
| gi|162462586 | superoxide dismutase [Cu-Zn] [Zea mays] | 5.4600 | 15193.4 | 134 | 100 | 166 | 100 | 6 |
| **Peptide Information** | | | | | | | | |
| **Calc. Mass** | **Obsrv. Mass** | **± da** | **± ppm** | **Start Seq.** | **End Seq.** | **Sequence** | **Total Ion Score** | **Modification** |
| 1153.4868 | 1153.4991 | 0.0123 | 11 | 69 | 78 | EHGAPEDENR |  |  |
| 1329.7737 | 1329.7493 | -0.0244 | -18 | 2 | 15 | VKAVAVLGSSDGVK |  |  |
| 1335.6903 | 1335.6957 | 0.0054 | 4 | 115 | 127 | AVVVHADPDDLGK |  |  |
| 1687.8545 | 1687.8784 | 0.0239 | 14 | 135 | 152 | STGNAGGRVACGIIGLQG |  | Carbamidomethyl (C)[11] |
| 1687.8545 | 1687.8784 | 0.0239 | 14 | 135 | 152 | STGNAGGRVACGIIGLQG | 2 | Carbamidomethyl (C)[11] |
| 2044.0459 | 2044.0405 | -0.0054 | -3 | 115 | 134 | AVVVHADPDDLGKGGHELSK |  |  |
| 2044.0459 | 2044.0405 | -0.0054 | -3 | 115 | 134 | AVVVHADPDDLGKGGHELSK | 85 |  |
| 3514.7886 | 3514.8833 | 0.0947 | 27 | 79 | 114 | HAGDLGNVTAGADGVANINVTDSQIPLTGPNSIIGR |  |  |
| 3514.7886 | 3514.8833 | 0.0947 | 27 | 79 | 114 | HAGDLGNVTAGADGVANINVTDSQIPLTGPNSIIGR | 38 |  |

| **Gel Idx/Pos** | **1706** |  |  |  |  |  |  |  |
| --- | --- | --- | --- | --- | --- | --- | --- | --- |
| **Accession No.** | **Protein Name** | **Protein PI** | **Protein MW** | **Protein Score** | **Protein Score C. I. %** | **Total Ion Score** | **Total Ion C. I. %** | **Pep. Count** |
| gi|194690760 | unknown [Zea mays] | 9.18 | 24914.4 | 116 | 100 | 80 | 100 | 7 |
| **Peptide Information** | | | | | | | | |
| **Calc. Mass** | **Obsrv. Mass** | **± da** | **± ppm** | **Start Seq.** | **End Seq.** | **Sequence** | **Total Ion Score** | **Modification** |
| 824.4625 | 824.4589 | -0.0036 | -4 | 187 | 193 | VEPVPQR |  |  |
| 824.4625 | 824.4589 | -0.0036 | -4 | 187 | 193 | VEPVPQR | 9 |  |
| 924.4169 | 924.4328 | 0.0159 | 17 | 211 | 217 | ENQSYQR |  |  |
| 924.4169 | 924.4328 | 0.0159 | 17 | 211 | 217 | ENQSYQR | 12 |  |
| 980.5636 | 980.5722 | 0.0086 | 9 | 186 | 193 | RVEPVPQR |  |  |
| 980.5636 | 980.5722 | 0.0086 | 9 | 186 | 193 | RVEPVPQR | 33 |  |
| 1040.483 | 1040.504 | 0.021 | 20 | 121 | 128 | IYNVSCER |  | Carbamidomethyl (C)[6] |
| 1040.483 | 1040.504 | 0.021 | 20 | 121 | 128 | IYNVSCER | 26 | Carbamidomethyl (C)[6] |
| 1080.5181 | 1080.5209 | 0.0028 | 3 | 210 | 217 | RENQSYQR |  |  |
| 1177.5961 | 1177.5875 | -0.0086 | -7 | 49 | 58 | RPDSSYSPLR |  |  |
| 1427.8152 | 1427.6752 | -0.14 | -98 | 7 | 19 | TLVAACPARSLLR |  | Carbamidomethyl (C)[6] |

| **Gel Idx/Pos** | **1547** |  |  |  |  |  |  |  |
| --- | --- | --- | --- | --- | --- | --- | --- | --- |
| **Accession No.** | **Protein Name** | **Protein PI** | **Protein MW** | **Protein Score** | **Protein Score C. I. %** | **Total Ion Score** | **Total Ion C. I. %** | **Pep. Count** |
| gi|195622322 | glutathione S-transferase III [Zea mays] | 5.96 | 23879.7 | 189 | 100 | 129 | 100 | 8 |
| **Peptide Information** | | | | | | | | |
| **Calc. Mass** | **Obsrv. Mass** | **± da** | **± ppm** | **Start Seq.** | **End Seq.** | **Sequence** | **Total Ion Score** | **Modification** |
| 873.504 | 873.5005 | -0.0035 | -4 | 18 | 25 | VATVLNEK |  |  |
| 1035.5946 | 1035.5939 | -0.0007 | -1 | 70 | 78 | AINRYIASK |  |  |
| 1285.6899 | 1285.7415 | 0.0516 | 40 | 143 | 153 | VLDVYEAHLAR |  |  |
| 1285.6899 | 1285.7415 | 0.0516 | 40 | 143 | 153 | VLDVYEAHLAR | 103 |  |
| 1361.7246 | 1361.7538 | 0.0292 | 21 | 6 | 17 | LYGMPLSPNVVR |  | Oxidation (M)[4] |
| 1404.7733 | 1404.7606 | -0.0127 | -9 | 207 | 221 | TVAAIPLPPPPSSSA |  |  |
| 1665.833 | 1665.9001 | 0.0671 | 40 | 79 | 95 | YASEGTDLLPATASAAK |  |  |
| 1770.9935 | 1771.0814 | 0.0879 | 50 | 2 | 17 | APLKLYGMPLSPNVVR |  | Oxidation (M)[8] |
| 1770.9935 | 1771.0814 | 0.0879 | 50 | 2 | 17 | APLKLYGMPLSPNVVR | 26 | Oxidation (M)[8] |
| 1811.9539 | 1812.0977 | 0.1438 | 79 | 26 | 42 | GLDFEIVPVDLTTGAHK |  |  |

| **Gel Idx/Pos** | **1309** |  |  |  |  |  |  |  |
| --- | --- | --- | --- | --- | --- | --- | --- | --- |
| **Accession No.** | **Protein Name** | **Protein PI** | **Protein MW** | **Protein Score** | **Protein Score C. I. %** | **Total Ion Score** | **Total Ion C. I. %** | **Pep. Count** |
| gi|195628632 | remorin [Zea mays] | 5.74 | 21875.4 | 98 | 99.998 | 70 | 99.997 | 5 |
| **Peptide Information** | | | | | | | | |
| **Calc. Mass** | **Obsrv. Mass** | **± da** | **± ppm** | **Start Seq.** | **End Seq.** | **Sequence** | **Total Ion Score** | **Modification** |
| 804.4097 | 804.3673 | -0.0424 | -53 | 2 | 8 | AEEEAKK |  |  |
| 935.4502 | 935.4248 | -0.0254 | -27 | 1 | 8 | MAEEEAKK |  |  |
| 951.4451 | 951.4514 | 0.0063 | 7 | 1 | 8 | MAEEEAKK |  | Oxidation (M)[1] |
| 1372.6927 | 1372.7081 | 0.0154 | 11 | 70 | 82 | QGGSNDRDLALAR |  |  |
| 1372.6927 | 1372.7081 | 0.0154 | 11 | 70 | 82 | QGGSNDRDLALAR | 31 |  |
| 1419.7478 | 1419.7448 | -0.003 | -2 | 56 | 69 | VADEPAPEKPAPAK |  |  |
| 2144.196 | 2144.2253 | 0.0293 | 14 | 49 | 69 | ALAIVEKVADEPAPEKPAPAK |  |  |
| 2144.196 | 2144.2253 | 0.0293 | 14 | 49 | 69 | ALAIVEKVADEPAPEKPAPAK | 39 |  |

| **Gel Idx/Pos** | **1553** |  |  |  |  |  |  |  |
| --- | --- | --- | --- | --- | --- | --- | --- | --- |
| **Accession No.** | **Protein Name** | **Protein PI** | **Protein MW** | **Protein Score** | **Protein Score C. I. %** | **Total Ion Score** | **Total Ion C. I. %** | **Pep. Count** |
| gi|8118441 | germin-like protein 1 [Zea mays] | 9.99 | 10068.3 | 113 | 100 | 81 | 100 | 4 |
| **Peptide Information** | | | | | | | | |
| **Calc. Mass** | **Obsrv. Mass** | **± da** | **± ppm** | **Start Seq.** | **End Seq.** | **Sequence** | **Total Ion Score** | **Modification** |
| 1054.5527 | 1054.517 | -0.0357 | -34 | 81 | 90 | VGSGEVEHIK |  |  |
| 1098.5803 | 1098.5942 | 0.0139 | 13 | 30 | 38 | GLVHFQQNR |  |  |
| 1098.5803 | 1098.5942 | 0.0139 | 13 | 30 | 38 | GLVHFQQNR | 24 |  |
| 1308.6947 | 1308.7244 | 0.0297 | 23 | 18 | 29 | TVATGDVFVFPR |  |  |
| 1308.6947 | 1308.7244 | 0.0297 | 23 | 18 | 29 | TVATGDVFVFPR | 57 |  |
| 1428.7594 | 1428.7784 | 0.019 | 13 | 78 | 90 | AFRVGSGEVEHIK |  |  |

| **Gel Idx/Pos** | **469** |  |  |  |  |  |  |  |
| --- | --- | --- | --- | --- | --- | --- | --- | --- |
| **Accession No.** | **Protein Name** | **Protein PI** | **Protein MW** | **Protein Score** | **Protein Score C. I. %** | **Total Ion Score** | **Total Ion C. I. %** | **Pep. Count** |
| gi|75140229 | RecName: Full=Transketolase, chloroplastic; Short=TK | 5.47 | 73346.7 | 889 | 100 | 586 | 100 | 31 |
| **Peptide Information** | | | | | | | | |
| **Calc. Mass** | **Obsrv. Mass** | **± da** | **± ppm** | **Start Seq.** | **End Seq.** | **Sequence** | **Total Ion Score** | **Modification** |
| 802.4417 | 802.437 | -0.0047 | -6 | 580 | 586 | AADELRK |  |  |
| 872.3971 | 872.4185 | 0.0214 | 25 | 403 | 409 | MFGDFQK |  |  |
| 914.4617 | 914.4694 | 0.0077 | 8 | 326 | 332 | FAEYEKK |  |  |
| 931.5095 | 931.4986 | -0.0109 | -12 | 10 | 18 | AATGELLEK |  |  |
| 967.4731 | 967.4836 | 0.0105 | 11 | 333 | 341 | YADDAATLK |  |  |
| 996.4686 | 996.4827 | 0.0141 | 14 | 63 | 69 | NPYWFNR |  |  |
| 996.4686 | 996.4827 | 0.0141 | 14 | 63 | 69 | NPYWFNR | 46 |  |
| 1005.5615 | 1005.559 | -0.0025 | -2 | 25 | 33 | FLAIDAVEK |  |  |
| 1005.5615 | 1005.559 | -0.0025 | -2 | 25 | 33 | FLAIDAVEK | 31 |  |
| 1012.6262 | 1012.6204 | -0.0058 | -6 | 525 | 533 | RPSILALSR |  |  |
| 1085.6565 | 1085.6392 | -0.0173 | -16 | 247 | 256 | AVTDKPTLIK |  |  |
| 1113.6263 | 1113.6204 | -0.0059 | -5 | 611 | 621 | ESVLPAAVTAR |  |  |
| 1124.4967 | 1124.5205 | 0.0238 | 21 | 230 | 239 | NGNTGYDDIR |  |  |
| 1193.5433 | 1193.572 | 0.0287 | 24 | 359 | 369 | YTPESPGDATR |  |  |
| 1193.5433 | 1193.572 | 0.0287 | 24 | 359 | 369 | YTPESPGDATR | 80 |  |
| 1221.6475 | 1221.6621 | 0.0146 | 12 | 257 | 268 | VTTTIGFGSPNK |  |  |
| 1267.5967 | 1267.6323 | 0.0356 | 28 | 63 | 71 | NPYWFNRDR |  |  |
| 1361.6808 | 1361.7036 | 0.0228 | 17 | 269 | 282 | ANSYSVHGSALGAK |  |  |
| 1389.7373 | 1389.7511 | 0.0138 | 10 | 622 | 634 | ISIEAGSTLGWQK |  |  |
| 1389.7373 | 1389.7511 | 0.0138 | 10 | 622 | 634 | ISIEAGSTLGWQK | 86 |  |
| 1464.7944 | 1464.7886 | -0.0058 | -4 | 660 | 673 | EYGITVESIIAAAK |  |  |
| 1476.8057 | 1476.8082 | 0.0025 | 2 | 536 | 549 | LPHLPGTSIEGVEK |  |  |
| 1573.6952 | 1573.6798 | -0.0154 | -10 | 403 | 415 | MFGDFQKDTAEER |  |  |
| 1573.6952 | 1573.8207 | 0.1255 | 80 | 403 | 415 | MFGDFQKDTAEER |  |  |
| 1573.6952 | 1573.8207 | 0.1255 | 80 | 403 | 415 | MFGDFQKDTAEER | 86 |  |
| 1589.6901 | 1589.7318 | 0.0417 | 26 | 403 | 415 | MFGDFQKDTAEER |  | Oxidation (M)[1] |
| 1589.6901 | 1589.7318 | 0.0417 | 26 | 403 | 415 | MFGDFQKDTAEER | 66 | Oxidation (M)[1] |
| 1601.8857 | 1601.8757 | -0.01 | -6 | 10 | 24 | AATGELLEKSVNTIR |  |  |
| 1609.7605 | 1609.72 | -0.0405 | -25 | 311 | 325 | HTPEGAALEADWNAK |  |  |
| 1609.7605 | 1609.8729 | 0.1124 | 70 | 311 | 325 | HTPEGAALEADWNAK |  |  |
| 1732.9592 | 1732.9625 | 0.0033 | 2 | 534 | 549 | QKLPHLPGTSIEGVEK |  |  |
| 1779.964 | 1779.9934 | 0.0294 | 17 | 642 | 659 | AIGIDKFGASAPAGTIYK |  |  |
| 1796.9794 | 1796.9873 | 0.0079 | 4 | 342 | 358 | SIITGELPTGWVDALPK |  |  |
| 1796.9794 | 1796.9873 | 0.0079 | 4 | 342 | 358 | SIITGELPTGWVDALPK | 102 |  |
| 2054.9858 | 2054.9749 | -0.0109 | -5 | 289 | 305 | QNLGWPYDTFFVPEDVK |  |  |
| 2233.1104 | 2233.2266 | 0.1162 | 52 | 497 | 517 | AMPNILMLRPADGNETAGAYK |  |  |
| 2249.1052 | 2249.1362 | 0.031 | 14 | 497 | 517 | AMPNILMLRPADGNETAGAYK |  | Oxidation (M)[2] |
| 2740.3254 | 2740.4233 | 0.0979 | 36 | 283 | 305 | EVEATRQNLGWPYDTFFVPEDVK |  |  |
| 3038.4988 | 3038.574 | 0.0752 | 25 | 550 | 579 | GGYTISDNSTGNKPDLIVMGTGSELEIAAK |  |  |
| 3054.4937 | 3054.5442 | 0.0505 | 17 | 550 | 579 | GGYTISDNSTGNKPDLIVMGTGSELEIAAK |  | Oxidation (M)[19] |
| 3057.4324 | 3057.5452 | 0.1128 | 37 | 191 | 217 | LIAFYDDNHISIDGDTEIAFTEDVSTR |  |  |
| 3301.6265 | 3301.7659 | 0.1394 | 42 | 593 | 621 | VVSFVSWELFDEQSDEYKESVLPAAVTAR |  |  |
| 3301.6265 | 3301.7659 | 0.1394 | 42 | 593 | 621 | VVSFVSWELFDEQSDEYKESVLPAAVTAR | 154 |  |
| 3471.7754 | 3471.9031 | 0.1277 | 37 | 110 | 144 | TPGHPENFETPGVEVTT |  |  |

| **Gel Idx/Pos** | **482** |  |  |  |  |  |  |  |
| --- | --- | --- | --- | --- | --- | --- | --- | --- |
| **Accession No.** | **Protein Name** | **Protein PI** | **Protein MW** | **Protein Score** | **Protein Score C. I. %** | **Total Ion Score** | **Total Ion C. I. %** | **Pep. Count** |
| gi|28948384 | Chain C, Maize Transketolase In Complex With Tpp | 5.47 | 73346.7 | 713 | 100 | 451 | 100 | 29 |
| **Peptide Information** | | | | | | | | |
| **Calc. Mass** | **Obsrv. Mass** | **± da** | **± ppm** | **Start Seq.** | **End Seq.** | **Sequence** | **Total Ion Score** | **Modification** |
| 802.4417 | 802.4785 | 0.0368 | 46 | 580 | 586 | AADELRK |  |  |
| 872.3971 | 872.4606 | 0.0635 | 73 | 403 | 409 | MFGDFQK |  |  |
| 914.4617 | 914.4923 | 0.0306 | 33 | 326 | 332 | FAEYEKK |  |  |
| 931.5095 | 931.549 | 0.0395 | 42 | 10 | 18 | AATGELLEK |  |  |
| 967.4731 | 967.5074 | 0.0343 | 35 | 333 | 341 | YADDAATLK |  |  |
| 996.4686 | 996.5043 | 0.0357 | 36 | 63 | 69 | NPYWFNR |  |  |
| 996.4686 | 996.5043 | 0.0357 | 36 | 63 | 69 | NPYWFNR | 41 |  |
| 1005.5615 | 1005.5917 | 0.0302 | 30 | 25 | 33 | FLAIDAVEK |  |  |
| 1005.5615 | 1005.5917 | 0.0302 | 30 | 25 | 33 | FLAIDAVEK | 48 |  |
| 1012.6262 | 1012.6555 | 0.0293 | 29 | 525 | 533 | RPSILALSR |  |  |
| 1012.6262 | 1012.6555 | 0.0293 | 29 | 525 | 533 | RPSILALSR | 35 |  |
| 1085.6565 | 1085.6635 | 0.007 | 6 | 247 | 256 | AVTDKPTLIK |  |  |
| 1124.4967 | 1124.5399 | 0.0432 | 38 | 230 | 239 | NGNTGYDDIR |  |  |
| 1140.7212 | 1140.7445 | 0.0233 | 20 | 524 | 533 | KRPSILALSR |  |  |
| 1193.5433 | 1193.5885 | 0.0452 | 38 | 359 | 369 | YTPESPGDATR |  |  |
| 1193.5433 | 1193.5885 | 0.0452 | 38 | 359 | 369 | YTPESPGDATR | 63 |  |
| 1221.6475 | 1221.6821 | 0.0346 | 28 | 257 | 268 | VTTTIGFGSPNK |  |  |
| 1267.5967 | 1267.6483 | 0.0516 | 41 | 63 | 71 | NPYWFNRDR |  |  |
| 1361.6808 | 1361.7273 | 0.0465 | 34 | 269 | 282 | ANSYSVHGSALGAK |  |  |
| 1389.7373 | 1389.7766 | 0.0393 | 28 | 622 | 634 | ISIEAGSTLGWQK |  |  |
| 1464.7944 | 1464.8196 | 0.0252 | 17 | 660 | 673 | EYGITVESIIAAAK |  |  |
| 1476.8057 | 1476.8309 | 0.0252 | 17 | 536 | 549 | LPHLPGTSIEGVEK |  |  |
| 1486.7842 | 1486.8035 | 0.0193 | 13 | 218 | 229 | FEALGWHTIWVK |  |  |
| 1498.7227 | 1498.7985 | 0.0758 | 51 | 59 | 69 | YNPKNPYWFNR |  |  |
| 1573.6952 | 1573.7524 | 0.0572 | 36 | 403 | 415 | MFGDFQKDTAEER |  |  |
| 1573.6952 | 1573.7524 | 0.0572 | 36 | 403 | 415 | MFGDFQKDTAEER | 104 |  |
| 1589.6901 | 1589.7439 | 0.0538 | 34 | 403 | 415 | MFGDFQKDTAEER |  | Oxidation (M)[1] |
| 1589.6901 | 1589.7439 | 0.0538 | 34 | 403 | 415 | MFGDFQKDTAEER | 78 | Oxidation (M)[1] |
| 1601.8857 | 1601.8915 | 0.0058 | 4 | 10 | 24 | AATGELLEKSVNTIR |  |  |
| 1609.7605 | 1609.812 | 0.0515 | 32 | 311 | 325 | HTPEGAALEADWNAK |  |  |
| 1609.7605 | 1609.812 | 0.0515 | 32 | 311 | 325 | HTPEGAALEADWNAK | 100 |  |
| 1732.9592 | 1733.0103 | 0.0511 | 29 | 534 | 549 | QKLPHLPGTSIEGVEK |  |  |
| 1732.9592 | 1733.0103 | 0.0511 | 29 | 534 | 549 | QKLPHLPGTSIEGVEK | 59 |  |
| 1779.964 | 1780.0125 | 0.0485 | 27 | 642 | 659 | AIGIDKFGASAPAGTIYK |  |  |
| 1796.9794 | 1797.0287 | 0.0493 | 27 | 342 | 358 | SIITGELPTGWVDALPK |  |  |
| 2233.1104 | 2233.1973 | 0.0869 | 39 | 497 | 517 | AMPNILMLRPADGNETAGAYK |  |  |
| 2249.1052 | 2249.1521 | 0.0469 | 21 | 497 | 517 | AMPNILMLRPADGNETAGAYK |  | Oxidation (M)[2] |
| 2265.1003 | 2265.1262 | 0.0259 | 11 | 497 | 517 | AMPNILMLRPADGNETAGAYK |  | Oxidation (M)[2,7] |
| 2740.3254 | 2740.4624 | 0.137 | 50 | 283 | 305 | EVEATRQNLGWPYDTFFVPEDVK |  |  |
| 3038.4988 | 3038.6423 | 0.1435 | 47 | 550 | 579 | GGYTISDNSTGNKPDLIVMGTGSELEIAAK |  |  |
| 3054.4937 | 3054.6233 | 0.1296 | 42 | 550 | 579 | GGYTISDNSTGNKPDLIVMGTGSELEIAAK |  | Oxidation (M)[19] |

| **Gel Idx/Pos** | **578** |  |  |  |  |  |  |  |
| --- | --- | --- | --- | --- | --- | --- | --- | --- |
| **Accession No.** | **Protein Name** | **Protein PI** | **Protein MW** | **Protein Score** | **Protein Score C. I. %** | **Total Ion Score** | **Total Ion C. I. %** | **Pep. Count** |
| gi|28948384 | Chain C, Maize Transketolase In Complex With Tpp | 5.47 | 73346.7 | 772 | 100 | 460 | 100 | 32 |
| **Peptide Information** | | | | | | | | |
| **Calc. Mass** | **Obsrv. Mass** | **± da** | **± ppm** | **Start Seq.** | **End Seq.** | **Sequence** | **Total Ion Score** | **Modification** |
| 802.4417 | 802.4633 | 0.0216 | 27 | 580 | 586 | AADELRK |  |  |
| 914.4617 | 914.489 | 0.0273 | 30 | 326 | 332 | FAEYEKK |  |  |
| 931.5095 | 931.5216 | 0.0121 | 13 | 10 | 18 | AATGELLEK |  |  |
| 967.4731 | 967.5092 | 0.0361 | 37 | 333 | 341 | YADDAATLK |  |  |
| 996.4686 | 996.4901 | 0.0215 | 22 | 63 | 69 | NPYWFNR |  |  |
| 996.4686 | 996.4901 | 0.0215 | 22 | 63 | 69 | NPYWFNR | 48 |  |
| 1005.5615 | 1005.572 | 0.0105 | 10 | 25 | 33 | FLAIDAVEK |  |  |
| 1005.5615 | 1005.572 | 0.0105 | 10 | 25 | 33 | FLAIDAVEK | 39 |  |
| 1012.6262 | 1012.639 | 0.0128 | 13 | 525 | 533 | RPSILALSR |  |  |
| 1012.6262 | 1012.639 | 0.0128 | 13 | 525 | 533 | RPSILALSR | 34 |  |
| 1064.5385 | 1064.5802 | 0.0417 | 39 | 102 | 109 | QFRQWGSR |  |  |
| 1085.6565 | 1085.6443 | -0.0122 | -11 | 247 | 256 | AVTDKPTLIK |  |  |
| 1113.6263 | 1113.6185 | -0.0078 | -7 | 611 | 621 | ESVLPAAVTAR |  |  |
| 1124.4967 | 1124.5203 | 0.0236 | 21 | 230 | 239 | NGNTGYDDIR |  |  |
| 1140.7212 | 1140.7347 | 0.0135 | 12 | 524 | 533 | KRPSILALSR |  |  |
| 1140.7212 | 1140.7347 | 0.0135 | 12 | 524 | 533 | KRPSILALSR | 12 |  |
| 1193.5433 | 1193.5623 | 0.019 | 16 | 359 | 369 | YTPESPGDATR |  |  |
| 1193.5433 | 1193.5623 | 0.019 | 16 | 359 | 369 | YTPESPGDATR | 78 |  |
| 1221.6475 | 1221.6561 | 0.0086 | 7 | 257 | 268 | VTTTIGFGSPNK |  |  |
| 1267.5967 | 1267.6272 | 0.0305 | 24 | 63 | 71 | NPYWFNRDR |  |  |
| 1361.6808 | 1361.7029 | 0.0221 | 16 | 269 | 282 | ANSYSVHGSALGAK |  |  |
| 1389.7373 | 1389.7457 | 0.0084 | 6 | 622 | 634 | ISIEAGSTLGWQK |  |  |
| 1464.7944 | 1464.7976 | 0.0032 | 2 | 660 | 673 | EYGITVESIIAAAK |  |  |
| 1476.8057 | 1476.8113 | 0.0056 | 4 | 536 | 549 | LPHLPGTSIEGVEK |  |  |
| 1486.7842 | 1486.8075 | 0.0233 | 16 | 218 | 229 | FEALGWHTIWVK |  |  |
| 1498.7227 | 1498.7766 | 0.0539 | 36 | 59 | 69 | YNPKNPYWFNR |  |  |
| 1573.6952 | 1573.7306 | 0.0354 | 22 | 403 | 415 | MFGDFQKDTAEER |  |  |
| 1573.6952 | 1573.7306 | 0.0354 | 22 | 403 | 415 | MFGDFQKDTAEER | 101 |  |
| 1589.6901 | 1589.7192 | 0.0291 | 18 | 403 | 415 | MFGDFQKDTAEER |  | Oxidation (M)[1] |
| 1601.8857 | 1601.8718 | -0.0139 | -9 | 10 | 24 | AATGELLEKSVNTIR |  |  |
| 1609.7605 | 1609.7939 | 0.0334 | 21 | 311 | 325 | HTPEGAALEADWNAK |  |  |
| 1732.9592 | 1732.9834 | 0.0242 | 14 | 534 | 549 | QKLPHLPGTSIEGVEK |  |  |
| 1732.9592 | 1732.9834 | 0.0242 | 14 | 534 | 549 | QKLPHLPGTSIEGVEK | 68 |  |
| 1779.964 | 1780.0096 | 0.0456 | 26 | 642 | 659 | AIGIDKFGASAPAGTIYK |  |  |
| 1796.9794 | 1797.0051 | 0.0257 | 14 | 342 | 358 | SIITGELPTGWVDALPK |  |  |
| 1796.9794 | 1797.0051 | 0.0257 | 14 | 342 | 358 | SIITGELPTGWVDALPK | 81 |  |
| 2233.1104 | 2233.2253 | 0.1149 | 51 | 497 | 517 | AMPNILMLRPADGNETAGAYK |  |  |
| 2249.1052 | 2249.1953 | 0.0901 | 40 | 497 | 517 | AMPNILMLRPADGNETAGAYK |  | Oxidation (M)[2] |
| 2265.1003 | 2265.1643 | 0.064 | 28 | 497 | 517 | AMPNILMLRPADGNETAGAYK |  | Oxidation (M)[2,7] |
| 2564.3103 | 2564.4338 | 0.1235 | 48 | 257 | 282 | VTTTIGFGSPNKANSYSVHGSALGAK |  |  |
| 2740.3254 | 2740.4678 | 0.1424 | 52 | 283 | 305 | EVEATRQNLGWPYDTFFVPEDVK |  |  |
| 3038.4988 | 3038.7175 | 0.2187 | 72 | 550 | 579 | GGYTISDNSTGNKPDLIVMGTGSELEIAAK |  |  |
| 3054.4937 | 3054.625 | 0.1313 | 43 | 550 | 579 | GGYTISDNSTGNKPDLIVMGTGSELEIAAK |  | Oxidation (M)[19] |
| 3471.7754 | 3472.022 | 0.2466 | 71 | 110 | 144 | TPGHPENFETPGVEVTTGPLGQGIANAVGLALAEK |  |  |

| **Gel Idx/Pos** | **726** |  |  |  |  |  |  |  |
| --- | --- | --- | --- | --- | --- | --- | --- | --- |
| **Accession No.** | **Protein Name** | **Protein PI** | **Protein MW** | **Protein Score** | **Protein Score C. I. %** | **Total Ion Score** | **Total Ion C. I. %** | **Pep. Count** |
| gi|343227637 | beta-D-glucosidase precursor [Zea mays] | 6.75 | 63463.4 | 585 | 100 | 444 | 100 | 19 |
| **Peptide Information** | | | | | | | | |
| **Calc. Mass** | **Obsrv. Mass** | **± da** | **± ppm** | **Start Seq.** | **End Seq.** | **Sequence** | **Total Ion Score** | **Modification** |
| 807.4359 | 807.4657 | 0.0298 | 37 | 474 | 479 | LDYIQR |  |  |
| 807.4359 | 807.4657 | 0.0298 | 37 | 474 | 479 | LDYIQR | 30 |  |
| 956.4836 | 956.4343 | -0.0493 | -52 | 516 | 523 | YGIAYVDR |  |  |
| 963.537 | 963.563 | 0.026 | 27 | 473 | 479 | RLDYIQR |  |  |
| 972.4244 | 972.4561 | 0.0317 | 33 | 339 | 346 | GDYPFSMR |  |  |
| 988.4193 | 988.4448 | 0.0255 | 26 | 339 | 346 | GDYPFSMR |  |  |
| 988.4193 | 988.4448 | 0.0255 | 26 | 339 | 346 | GDYPFSMR | 10 | Oxidation (M)[7] |
| 1004.3812 | 1004.4262 | 0.045 | 45 | 132 | 139 | EMGMDAYR |  | Oxidation (M)[7] |
| 1094.5664 | 1094.5844 | 0.018 | 16 | 299 | 308 | IGLAFDVMGR |  | Oxidation (M)[2,4] |
| 1094.5664 | 1094.5844 | 0.018 | 16 | 299 | 308 | IGLAFDVMGR | 25 | Oxidation (M)[8] |
| 1138.5891 | 1138.6138 | 0.0247 | 22 | 351 | 359 | ERLPFFSDK |  | Oxidation (M)[8] |
| 1138.5891 | 1138.6138 | 0.0247 | 22 | 351 | 359 | ERLPFFSDK | 37 |  |
| 1211.642 | 1211.6578 | 0.0158 | 13 | 207 | 216 | IVNDYKNFAK |  |  |
| 1328.6304 | 1328.6521 | 0.0217 | 16 | 217 | 227 | VCFDNFGDKVK |  |  |
| 1358.6443 | 1358.6707 | 0.0264 | 19 | 129 | 139 | LLKEMGMDAYR |  | Carbamidomethyl (C)[2] |
| 1366.7002 | 1366.7059 | 0.0057 | 4 | 353 | 363 | LPFFSDKQQEK |  | Oxidation (M)[5,7] |
| 1454.7162 | 1454.7358 | 0.0196 | 13 | 309 | 321 | VPYGTSFLDEQAK |  |  |
| 1634.8094 | 1634.8352 | 0.0258 | 16 | 459 | 472 | EKPLPMEAALNDYK |  |  |
| 1728.8076 | 1728.8224 | 0.0148 | 9 | 151 | 166 | GTVEGGINQDGIDYYK |  | Oxidation (M)[6] |
| 1739.8599 | 1739.9043 | 0.0444 | 26 | 309 | 323 | VPYGTSFLDEQAKER |  |  |
| 1739.8599 | 1739.9043 | 0.0444 | 26 | 309 | 323 | VPYGTSFLDEQAKER | 121 |  |
| 1790.9105 | 1790.9425 | 0.032 | 18 | 459 | 473 | EKPLPMEAALNDYKR |  |  |
| 1808.9501 | 1808.999 | 0.0489 | 27 | 47 | 63 | VGNENGVQLLSPSEIPR |  | Oxidation (M)[6] |
| 1808.9501 | 1808.999 | 0.0489 | 27 | 47 | 63 | VGNENGVQLLSPSEIPR | 123 |  |
| 1884.9087 | 1884.9615 | 0.0528 | 28 | 151 | 167 | GTVEGGINQDGIDYYKR |  |  |
| 1884.9087 | 1884.9615 | 0.0528 | 28 | 151 | 167 | GTVEGGINQDGIDYYKR | 100 |  |
| 1965.0513 | 1965.0959 | 0.0446 | 23 | 47 | 64 | VGNENGVQLLSPSEIPRR |  |  |

| **Gel Idx/Pos** | **749** |  |  |  |  |  |  |  |
| --- | --- | --- | --- | --- | --- | --- | --- | --- |
| **Accession No.** | **Protein Name** | **Protein PI** | **Protein MW** | **Protein Score** | **Protein Score C. I. %** | **Total Ion Score** | **Total Ion C. I. %** | **Pep. Count** |
| gi|359497202 | PREDICTED: T-complex protein 1 [Vitis vinifera] | 6.2 | 61032.7 | 86 | 100 | 68.369 | 0 | 9 |
| **Peptide Information** | | | | | | | | |
| **Calc. Mass** | **Obsrv. Mass** | **± da** | **± ppm** | **Start Seq.** | **End Seq.** | **Sequence** | **Total Ion Score** | **Modification** |
| 861.4611 | 861.5008 | 0.0397 | 46 | 202 | 209 | KVPGGTMR |  | Oxidation (M)[7] |
| 981.5588 | 981.5421 | -0.0167 | -17 | 425 | 433 | QHARTIAGK |  |  |
| 981.5588 | 981.5421 | -0.0167 | -17 | 425 | 433 | QHARTIAGK |  |  |
| 989.4898 | 989.53 | 0.0402 | 41 | 317 | 324 | VTEEDLQR |  |  |
| 989.4898 | 989.53 | 0.0402 | 41 | 317 | 324 | VTEEDLQR |  |  |
| 1076.6462 | 1076.6072 | -0.039 | -36 | 71 | 80 | LLDIVHPAAK |  |  |
| 1154.6351 | 1154.6672 | 0.0321 | 28 | 391 | 400 | SLHDAIMIVR |  |  |
| 1170.63 | 1170.6392 | 0.0092 | 8 | 391 | 400 | SLHDAIMIVR | 71 | Oxidation (M)[7] |
| 1196.6311 | 1196.6332 | 0.0021 | 2 | 210 | 220 | DSFLVNGVAFK |  |  |
| 1321.6019 | 1321.6436 | 0.0417 | 32 | 379 | 390 | GGADQFIEEAER |  |  |
| 1321.6019 | 1321.6436 | 0.0417 | 32 | 379 | 390 | GGADQFIEEAER | 24 | 24 |
| 1402.6638 | 1402.7015 | 0.0377 | 27 | 222 | 233 | TFSYAGFEQQPK |  |  |
| 1968.0009 | 1968.1 | 0.0991 | 50 | 361 | 378 | FNIFSGCPSGQTATIVLR |  | Carbamidomethyl (C)[7] |
| 1968.0009 | 1968.1 | 0.0991 | 50 | 361 | 378 | FNIFSGCPSGQTATIVLR |  | Carbamidomethyl (C)[7] |

| **Gel Idx/Pos** | **821** |  |  |  |  |  |  |  |
| --- | --- | --- | --- | --- | --- | --- | --- | --- |
| **Accession No.** | **Protein Name** | **Protein PI** | **Protein MW** | **Protein Score** | **Protein Score C. I. %** | **Total Ion Score** | **Total Ion C. I. %** | **Pep. Count** |
| gi|195625588 | cysteine synthase [Zea mays] | 8.74 | 40833.3 | 187 | 100 | 120 | 100 | 11 |
| **Peptide Information** | | | | | | | | |
| **Calc. Mass** | **Obsrv. Mass** | **± da** | **± ppm** | **Start Seq.** | **End Seq.** | **Sequence** | **Total Ion Score** | **Modification** |
| 849.3883 | 849.3771 | -0.0112 | -13 | 82 | 89 | VTEGCGAR |  | Carbamidomethyl (C)[5] |
| 881.4363 | 881.416 | -0.0203 | -23 | 190 | 196 | ATELYER |  |  |
| 946.4952 | 946.4816 | -0.0136 | -14 | 65 | 73 | DSASQLIGR |  |  |
| 1009.5312 | 1009.5209 | -0.0103 | -10 | 189 | 196 | KATELYER |  |  |
| 1215.6804 | 1215.6901 | 0.0097 | 8 | 63 | 73 | IRDSASQLIGR |  |  |
| 1215.6804 | 1215.6901 | 0.0097 | 8 | 63 | 73 | IRDSASQLIGR | 28 |  |
| 1294.7042 | 1294.7053 | 0.0011 | 1 | 94 | 104 | LEFLQPSFSVK |  |  |
| 1296.7886 | 1296.814 | 0.0254 | 20 | 348 | 359 | LIVTVLPSLGER |  |  |
| 1296.7886 | 1296.814 | 0.0254 | 20 | 348 | 359 | LIVTVLPSLGER | 43 |  |
| 1313.6736 | 1313.7053 | 0.0317 | 24 | 360 | 370 | YLSSALFDELR |  |  |
| 1316.7209 | 1316.6591 | -0.0618 | -47 | 169 | 181 | AFGANLVLTDPAK |  |  |
| 1316.7209 | 1316.7723 | 0.0514 | 39 | 169 | 181 | AFGANLVLTDPAK |  |  |
| 1441.7686 | 1441.8307 | 0.0621 | 43 | 360 | 371 | YLSSALFDELRK |  |  |
| 1441.7686 | 1441.8307 | 0.0621 | 43 | 360 | 371 | YLSSALFDELRK | 50 |  |
| 1630.8218 | 1630.7994 | -0.0224 | -14 | 105 | 118 | DRPAISMLEDAEKR |  |  |
| 1630.8218 | 1630.9662 | 0.1444 | 89 | 105 | 118 | DRPAISMLEDAEKR |  |  |

| **Gel Idx/Pos** | **882** |  |  |  |  |  |  |  |
| --- | --- | --- | --- | --- | --- | --- | --- | --- |
| **Accession No.** | **Protein Name** | **Protein PI** | **Protein MW** | **Protein Score** | **Protein Score C. I. %** | **Total Ion Score** | **Total Ion C. I. %** | **Pep. Count** |
| gi|226532728 | electron transporter protein [Zea mays] | 7.57 | 21197.7 | 89 | 99.55 | 65 | 99.897 | 3 |
| **Peptide Information** | | | | | | | | |
| **Calc. Mass** | **Obsrv. Mass** | **± da** | **± ppm** | **Start Seq.** | **End Seq.** | **Sequence** | **Total Ion Score** | **Modification** |
| 827.4046 | 827.4222 | 0.0176 | 21 | 186 | 191 | IHEWDK |  |  |
| 1382.7063 | 1382.712 | 0.0057 | 4 | 80 | 91 | LLPDGTPDVHYR |  |  |
| 1382.7063 | 1382.712 | 0.0057 | 4 | 80 | 91 | LLPDGTPDVHYR | 38 |  |
| 3099.4429 | 3099.5339 | 0.091 | 29 | 50 | 79 | AVEVDAPSAGAPEPEEAEEPSVDFAFVSPR |  |  |
| 3099.4429 | 3099.5339 | 0.091 | 29 | 50 | 79 | AVEVDAPSAGAPEPEEAEEPSVDFAFVSPR | 52 |  |

| **Gel Idx/Pos** | **931** |  |  |  |  |  |  |  |
| --- | --- | --- | --- | --- | --- | --- | --- | --- |
| **Accession No.** | **Protein Name** | **Protein PI** | **Protein MW** | **Protein Score** | **Protein Score C. I. %** | **Total Ion Score** | **Total Ion C. I. %** | **Pep. Count** |
| gi|11467200 | ribulose-1,5-bisphosphate carboxylase/oxygenase large subunit [Zea mays] | 6.33 | 53294.6 | 950 | 100 | 836 | 100 | 27 |
| **Peptide Information** | | | | | | | | |
| **Calc. Mass** | **Obsrv. Mass** | **± da** | **± ppm** | **Start Seq.** | **End Seq.** | **Sequence** | **Total Ion Score** | **Modification** |
| 912.472 | 912.4847 | 0.0127 | 14 | 296 | 303 | AMHAVIDR |  |  |
| 914.405 | 914.4255 | 0.0205 | 22 | 306 | 312 | NHGMHFR |  | Oxidation (M)[4] |
| 946.4839 | 946.4996 | 0.0157 | 17 | 228 | 236 | AQAETGEIK |  |  |
| 971.4073 | 971.4283 | 0.021 | 22 | 188 | 194 | ACYECLR |  | Carbamidomethyl (C)[2,5] |
| 971.4073 | 971.4283 | 0.021 | 22 | 188 | 194 | ACYECLR | 34 | Carbamidomethyl (C)[2,5] |
| 983.5197 | 983.4901 | -0.0296 | -30 | 464 | 471 | EIKFDGFK |  |  |
| 985.5789 | 985.592 | 0.0131 | 13 | 132 | 139 | ALRLEDLR |  |  |
| 985.5789 | 985.592 | 0.0131 | 13 | 132 | 139 | ALRLEDLR | 35 |  |
| 1021.5312 | 1021.5588 | 0.0276 | 27 | 33 | 41 | DTDILAAFR |  |  |
| 1021.5312 | 1021.5588 | 0.0276 | 27 | 33 | 41 | DTDILAAFR | 72 |  |
| 1037.4899 | 1037.527 | 0.0371 | 36 | 351 | 358 | DDFIEKDR |  |  |
| 1116.583 | 1116.6162 | 0.0332 | 30 | 422 | 431 | VALEACVQAR |  | Carbamidomethyl (C)[6] |
| 1116.583 | 1116.6162 | 0.0332 | 30 | 422 | 431 | VALEACVQAR | 75 | Carbamidomethyl (C)[6] |
| 1170.5586 | 1170.6744 | 0.1158 | 99 | 304 | 312 | QKNHGMHFR |  | Oxidation (M)[6] |
| 1187.6644 | 1187.7054 | 0.041 | 35 | 286 | 295 | DNGLLLHIHR |  |  |
| 1247.6129 | 1247.6428 | 0.0299 | 24 | 218 | 227 | FVFCAEAIYK |  | Carbamidomethyl (C)[4] |
| 1247.6129 | 1247.6428 | 0.0299 | 24 | 218 | 227 | FVFCAEAIYK | 51 | Carbamidomethyl (C)[4] |
| 1257.6797 | 1257.7312 | 0.0515 | 41 | 436 | 446 | DLAREGNEIIK |  |  |
| 1275.7307 | 1275.7723 | 0.0416 | 33 | 340 | 350 | EITLGFVDLLR |  |  |
| 1275.7307 | 1275.7723 | 0.0416 | 33 | 340 | 350 | EITLGFVDLLR | 99 |  |
| 1401.7737 | 1401.8202 | 0.0465 | 33 | 135 | 146 | LEDLRIPPAYSK |  |  |
| 1407.6678 | 1407.7085 | 0.0407 | 29 | 22 | 32 | LTYYTPEYETK |  |  |
| 1451.6219 | 1451.6956 | 0.0737 | 51 | 202 | 213 | DDENVNSQPFMR |  |  |
| 1465.7546 | 1465.817 | 0.0624 | 43 | 147 | 159 | TFQGPPHGIQVER |  |  |
| 1465.7546 | 1465.817 | 0.0624 | 43 | 147 | 159 | TFQGPPHGIQVER | 115 |  |
| 1481.7167 | 1481.8394 | 0.1227 | 83 | 320 | 334 | MSGGDHIHSGTVVGK |  |  |
| 1502.8512 | 1502.9182 | 0.067 | 45 | 165 | 177 | YGRPLLGCTIKPK |  | Carbamidomethyl (C)[8] |
| 1518.741 | 1518.8207 | 0.0797 | 52 | 216 | 227 | DRFVFCAEAIYK |  | Carbamidomethyl (C)[6] |
| 1534.7358 | 1534.7904 | 0.0546 | 36 | 451 | 463 | WSAELAAACEIWK |  | Carbamidomethyl (C)[9] |
| 1860.0226 | 1860.0095 | -0.0131 | -7 | 335 | 350 | LEGEREITLGFVDLLR |  |  |
| 1860.0226 | 1860.16 | 0.1374 | 74 | 335 | 350 | LEGEREITLGFVDLLR |  |  |
| 1950.916 | 1951.0042 | 0.0882 | 45 | 237 | 253 | GHYLNATAGTCEEMIKR |  | Carbamidomethyl (C)[11] |
| 2023.0747 | 2023.1958 | 0.1211 | 60 | 340 | 356 | EITLGFVDLLRDDFIEK |  |  |
| 2169.9871 | 2170.1069 | 0.1198 | 55 | 195 | 213 | GGLDFTKDDENVNSQPFMR |  |  |
| 2169.9871 | 2170.1069 | 0.1198 | 55 | 195 | 213 | GGLDFTKDDENVNSQPFMR | 131 |  |
| 2185.9819 | 2186.0103 | 0.0284 | 13 | 195 | 213 | GGLDFTKDDENVNSQPFMR |  | Oxidation (M)[18] |
| 2410.1814 | 2410.3411 | 0.1597 | 66 | 22 | 41 | LTYYTPEYETKDTDILAAFR |  |  |
| 2410.1814 | 2410.3411 | 0.1597 | 66 | 22 | 41 | LTYYTPEYETKDTDILAAFR | 148 |  |

| **Gel Idx/Pos** | **1031** |  |  |  |  |  |  |  |
| --- | --- | --- | --- | --- | --- | --- | --- | --- |
| **Accession No.** | **Protein Name** | **Protein PI** | **Protein MW** | **Protein Score** | **Protein Score C. I. %** | **Total Ion Score** | **Total Ion C. I. %** | **Pep. Count** |
| gi|195612198 | fructose-bisphosphate aldolase, cytoplasmic isozyme 1[Zea mays] | 6.26 | 38464 | 683 | 100 | 552 | 100 | 15 |
| **Peptide Information** | | | | | | | | |
| **Calc. Mass** | **Obsrv. Mass** | **± da** | **± ppm** | **Start Seq.** | **End Seq.** | **Sequence** | **Total Ion Score** | **Modification** |
| 829.3839 | 829.416 | 0.0321 | 39 | 133 | 139 | YYEAGAR |  |  |
| 829.3839 | 829.416 | 0.0321 | 39 | 133 | 139 | YYEAGAR | 50 |  |
| 881.4614 | 881.4778 | 0.0164 | 19 | 8 | 14 | YTDELIK |  |  |
| 886.4741 | 886.4998 | 0.0257 | 29 | 164 | 171 | QNVEGLAR |  |  |
| 886.4741 | 886.4998 | 0.0257 | 29 | 164 | 171 | QNVEGLAR | 52 |  |
| 999.6448 | 999.6337 | -0.0111 | -11 | 220 | 228 | VLLEATLLK |  |  |
| 1465.7759 | 1465.771 | -0.0049 | -3 | 149 | 163 | VGPAGQGQPSELAVR |  |  |
| 1465.7759 | 1465.771 | -0.0049 | -3 | 149 | 163 | VGPAGQGQPSELAVR | 126 |  |
| 1474.7496 | 1474.7439 | -0.0057 | -4 | 40 | 52 | LSSINLENVESNR |  |  |
| 1474.7496 | 1474.7439 | -0.0057 | -4 | 40 | 52 | LSSINLENVESNR | 23 |  |
| 1591.869 | 1591.8608 | -0.0082 | -5 | 241 | 255 | VSAEVIAEYTVAALR |  |  |
| 1591.869 | 1591.8608 | -0.0082 | -5 | 241 | 255 | VSAEVIAEYTVAALR | 99 |  |
| 1630.8507 | 1630.8442 | -0.0065 | -4 | 39 | 52 | RLSSINLENVESNR |  |  |
| 1666.9739 | 1666.9324 | -0.0415 | -25 | 214 | 228 | SLSDHKVLLEATLLK |  |  |
| 1747.9701 | 1747.9658 | -0.0043 | -2 | 241 | 256 | VSAEVIAEYTVAALRR |  |  |
| 1747.9701 | 1747.9658 | -0.0043 | -2 | 241 | 256 | VSAEVIAEYTVAALRR | 57 |  |
| 1877.0604 | 1877.0406 | -0.0198 | -11 | 145 | 163 | AVLKVGPAGQGQPSELAVR |  |  |
| 2069.1643 | 2069.1401 | -0.0242 | -12 | 83 | 103 | TSDGKPFVDVLIAGGVVPGIK |  |  |
| 2249.0894 | 2249.1025 | 0.0131 | 6 | 107 | 129 | GTVEIAGTDGETTTQGLDSLGAR |  |  |
| 2249.0894 | 2249.1025 | 0.0131 | 6 | 107 | 129 | GTVEIAGTDGETTTQGLDSLGAR | 147 |  |
| 2591.2795 | 2591.3081 | 0.0286 | 11 | 104 | 129 | VDKGTVEIAGTDGETTTQGLDSLGAR |  |  |
| 3782.8364 | 3782.9565 | 0.1201 | 32 | 172 | 206 | YALICQENGLVPIVEPEILTDGGHDIGTCAAATER |  | Carbamidomethyl (C)[5,29] |

| **Gel Idx/Pos** | **1069** |  |  |  |  |  |  |  |
| --- | --- | --- | --- | --- | --- | --- | --- | --- |
| **Accession No.** | **Protein Name** | **Protein PI** | **Protein MW** | **Protein Score** | **Protein Score C. I. %** | **Total Ion Score** | **Total Ion C. I. %** | **Pep. Count** |
| gi|195634659 | fructose-bisphosphate aldolase [Zea mays] | 7.63 | 41923.5 | 864 | 100 | 716 | 100 | 17 |
| **Peptide Information** | | | | | | | | |
| **Calc. Mass** | **Obsrv. Mass** | **± da** | **± ppm** | **Start Seq.** | **End Seq.** | **Sequence** | **Total Ion Score** | **Modification** |
| 873.4577 | 873.475 | 0.0173 | 20 | 193 | 200 | EAAWGLAR |  |  |
| 873.4577 | 873.475 | 0.0173 | 20 | 193 | 200 | EAAWGLAR | 65 |  |
| 901.5101 | 901.4832 | -0.0269 | -30 | 359 | 367 | ANSLAQLGK |  |  |
| 947.4979 | 947.511 | 0.0131 | 14 | 330 | 337 | ALQNTCLK |  | Carbamidomethyl (C)[6] |
| 970.5679 | 970.5774 | 0.0095 | 10 | 348 | 356 | AAQDALLLR |  |  |
| 970.5679 | 970.5774 | 0.0095 | 10 | 348 | 356 | AAQDALLLR | 76 |  |
| 1115.5845 | 1115.5916 | 0.0071 | 6 | 338 | 347 | TWGGQPDKVK |  |  |
| 1141.5007 | 1141.533 | 0.0323 | 28 | 368 | 378 | YTSDGEAAEAK |  |  |
| 1156.5382 | 1156.55 | 0.0118 | 10 | 163 | 172 | EAAYYQQGAR |  |  |
| 1156.5382 | 1156.55 | 0.0118 | 10 | 163 | 172 | EAAYYQQGAR | 82 |  |
| 1291.6893 | 1291.6742 | -0.0151 | -12 | 271 | 282 | ATPEQVAAYTLK |  |  |
| 1291.6893 | 1291.6742 | -0.0151 | -12 | 271 | 282 | ATPEQVAAYTLK | 54 |  |
| 1387.7175 | 1387.7168 | -0.0007 | -1 | 73 | 85 | LASIGLENTEANR |  |  |
| 1387.7175 | 1387.7168 | -0.0007 | -1 | 73 | 85 | LASIGLENTEANR | 116 |  |
| 1510.8475 | 1510.8247 | -0.0228 | -15 | 178 | 192 | TVVSIPNGPSELAVK |  |  |
| 1543.8187 | 1543.814 | -0.0047 | -3 | 72 | 85 | RLASIGLENTEANR |  |  |
| 1562.8174 | 1562.8113 | -0.0061 | -4 | 269 | 282 | DRATPEQVAAYTLK |  |  |
| 1562.8174 | 1562.8113 | -0.0061 | -4 | 269 | 282 | DRATPEQVAAYTLK | 34 |  |
| 1622.7626 | 1622.7561 | -0.0065 | -4 | 58 | 72 | GILAMDESNATCGKR |  | Carbamidomethyl (C)[12] |
| 1638.7574 | 1638.7316 | -0.0258 | -16 | 58 | 72 | GILAMDESNATCGKR |  | Carbamidomethyl (C)[12], Oxidation (M)[5] |
| 1848.832 | 1848.8112 | -0.0208 | -11 | 368 | 384 | YTSDGEAAEAKEGMFVK |  | Oxidation (M)[14] |
| 2401.1565 | 2401.1997 | 0.0432 | 18 | 140 | 162 | GLVPLAGSNNESWCQGLDGLASR |  | Carbamidomethyl (C)[14] |
| 2401.1565 | 2401.1997 | 0.0432 | 18 | 140 | 162 | GLVPLAGSNNESWCQGLDGLASR | 137 | Carbamidomethyl (C)[14] |
| 2743.3469 | 2743.4126 | 0.0657 | 24 | 137 | 162 | VDKGLVPLAGSNNESWCQGLDGLASR |  | Carbamidomethyl (C)[17] |
| 3047.5684 | 3047.6914 | 0.123 | 40 | 201 | 228 | YAAISQDNGLVPIVEPEILLDGEHGIER |  |  |
| 3047.5684 | 3047.6914 | 0.123 | 40 | 201 | 228 | YAAISQDNGLVPIVEPEILLDGEHGIER | 152 |  |

| **Gel Idx/Pos** | **1073** |  |  |  |  |  |  |  |
| --- | --- | --- | --- | --- | --- | --- | --- | --- |
| **Accession No.** | **Protein Name** | **Protein PI** | **Protein MW** | **Protein Score** | **Protein Score C. I. %** | **Total Ion Score** | **Total Ion C. I. %** | **Pep. Count** |
| gi|326378667 | histidine triad nucleotide binding protein [Zea mays] | 6.35 | 15250.6 | 124 | 100 | 158 | 100 | 4 |
| **Peptide Information** | | | | | | | | |
| **Calc. Mass** | **Obsrv. Mass** | **± da** | **± ppm** | **Start Seq.** | **End Seq.** | **Sequence** | **Total Ion Score** | **Modification** |
| 949.4738 | 949.4692 | -0.0046 | -5 | 85 | 89 | KVVAK |  |  |
| 949.4738 | 949.4692 | -0.0046 | -5 | 27 | 38 | EIPSTVVYEDEK |  |  |
| 957.5727 | 957.5565 | -0.0162 | -17 | 27 | 38 | EIPSTVVYEDEK | 69 |  |
| 1609.8909 | 1609.9384 | 0.0475 | 30 | 61 | 67 | DGLTGLAK |  |  |
| 1609.8909 | 1609.9384 | 0.0475 | 30 | 59 | 72 | VKDGLTGLAKAEER | 78 |  |
| 1737.9858 | 1738.0491 | 0.0633 | 36 | 59 | 72 | VKDGLTGLAKAEER |  |  |

| **Gel Idx/Pos** | **1083** |  |  |  |  |  |  |  |
| --- | --- | --- | --- | --- | --- | --- | --- | --- |
| **Accession No.** | **Protein Name** | **Protein PI** | **Protein MW** | **Protein Score** | **Protein Score C. I. %** | **Total Ion Score** | **Total Ion C. I. %** | **Pep. Count** |
| gi|195634659 | fructose-bisphosphate aldolase [Zea mays] | 7.63 | 41923.5 | 915 | 100 | 766 | 100 | 17 |
| **Peptide Information** | | | | | | | | |
| **Calc. Mass** | **Obsrv. Mass** | **± da** | **± ppm** | **Start Seq.** | **End Seq.** | **Sequence** | **Total Ion Score** | **Modification** |
| 873.4577 | 873.5159 | 0.0582 | 67 | 193 | 200 | EAAWGLAR |  |  |
| 873.4577 | 873.5159 | 0.0582 | 67 | 193 | 200 | EAAWGLAR | 65 |  |
| 901.5101 | 901.5151 | 0.005 | 6 | 359 | 367 | ANSLAQLGK |  |  |
| 947.4979 | 947.5483 | 0.0504 | 53 | 330 | 337 | ALQNTCLK |  | Carbamidomethyl (C)[6] |
| 970.5679 | 970.6136 | 0.0457 | 47 | 348 | 356 | AAQDALLLR |  |  |
| 970.5679 | 970.6136 | 0.0457 | 47 | 348 | 356 | AAQDALLLR | 72 |  |
| 1115.5845 | 1115.6208 | 0.0363 | 33 | 338 | 347 | TWGGQPDKVK |  |  |
| 1141.5007 | 1141.5554 | 0.0547 | 48 | 368 | 378 | YTSDGEAAEAK |  |  |
| 1156.5382 | 1156.5723 | 0.0341 | 29 | 163 | 172 | EAAYYQQGAR |  |  |
| 1156.5382 | 1156.5723 | 0.0341 | 29 | 163 | 172 | EAAYYQQGAR | 77 |  |
| 1291.6893 | 1291.6923 | 0.003 | 2 | 271 | 282 | ATPEQVAAYTLK |  |  |
| 1387.7175 | 1387.7222 | 0.0047 | 3 | 73 | 85 | LASIGLENTEANR |  |  |
| 1387.7175 | 1387.7222 | 0.0047 | 3 | 73 | 85 | LASIGLENTEANR | 132 |  |
| 1510.8475 | 1510.8197 | -0.0278 | -18 | 178 | 192 | TVVSIPNGPSELAVK |  |  |
| 1543.8187 | 1543.7999 | -0.0188 | -12 | 72 | 85 | RLASIGLENTEANR |  |  |
| 1562.8174 | 1562.7944 | -0.023 | -15 | 269 | 282 | DRATPEQVAAYTLK |  |  |
| 1562.8174 | 1562.7944 | -0.023 | -15 | 269 | 282 | DRATPEQVAAYTLK | 38 |  |
| 1622.7626 | 1622.731 | -0.0316 | -19 | 58 | 72 | GILAMDESNATCGKR |  | Carbamidomethyl (C)[12] |
| 1622.7626 | 1622.731 | -0.0316 | -19 | 58 | 72 | GILAMDESNATCGKR | 107 | Carbamidomethyl (C)[12] |
| 1638.7574 | 1638.7349 | -0.0225 | -14 | 58 | 72 | GILAMDESNATCGKR |  | Carbamidomethyl (C)[12], Oxidation (M)[5] |
| 1832.8372 | 1832.7981 | -0.0391 | -21 | 368 | 384 | YTSDGEAAEAKEGMFVK |  |  |
| 2401.1565 | 2401.1038 | -0.0527 | -22 | 140 | 162 | GLVPLAGSNNESWCQGLDGLASR |  | Carbamidomethyl (C)[14] |
| 2401.1565 | 2401.1038 | -0.0527 | -22 | 140 | 162 | GLVPLAGSNNESWCQGLDGLASR | 121 | Carbamidomethyl (C)[14] |
| 2743.3469 | 2743.3059 | -0.041 | -15 | 137 | 162 | VDKGLVPLAGSNNESWCQGLDGLASR |  | Carbamidomethyl (C)[17] |
| 3047.5684 | 3047.5547 | -0.0137 | -4 | 201 | 228 | YAAISQDNGLVPIVEPEILLDGEHGIER |  |  |
| 3047.5684 | 3047.5547 | -0.0137 | -4 | 201 | 228 | YAAISQDNGLVPIVEPEILLDGEHGIER | 155 |  |

| **Gel Idx/Pos** | **1165** |  |  |  |  |  |  |  |
| --- | --- | --- | --- | --- | --- | --- | --- | --- |
| **Accession No.** | **Protein Name** | **Protein PI** | **Protein MW** | **Protein Score** | **Protein Score C. I. %** | **Total Ion Score** | **Total Ion C. I. %** | **Pep. Count** |
| gi|195624056 | ferredoxin--NADP reductase, leaf isozyme [Zea mays] | 8.53 | 40976.5 | 111 | 100 | 58 | 99.947 | 10 |
| **Peptide Information** | | | | | | | | |
| **Calc. Mass** | **Obsrv. Mass** | **± da** | **± ppm** | **Start Seq.** | **End Seq.** | **Sequence** | **Total Ion Score** | **Modification** |
| 807.4359 | 807.4337 | -0.0022 | -3 | 284 | 290 | LDFAVSR |  |  |
| 807.4359 | 807.4337 | -0.0022 | -3 | 284 | 290 | LDFAVSR | 13 |  |
| 811.413 | 811.4249 | 0.0119 | 15 | 300 | 305 | MYIQTR |  |  |
| 1236.6736 | 1236.6161 | -0.0575 | -46 | 87 | 96 | YKPKEPYVGR |  |  |
| 1251.6005 | 1251.5712 | -0.0293 | -23 | 360 | 369 | KGEQWNVEVY |  |  |
| 1251.6005 | 1251.5712 | -0.0293 | -23 | 360 | 369 | KGEQWNVEVY | 45 |  |
| 1283.6089 | 1283.5909 | -0.018 | -14 | 347 | 356 | DGIDWMQYKK |  |  |
| 1378.7213 | 1378.6829 | -0.0384 | -28 | 173 | 184 | LVYTNDQGEIVK |  |  |
| 1378.7213 | 1378.6829 | -0.0384 | -28 | 173 | 184 | LVYTNDQGEIVK |  |  |
| 1534.8224 | 1534.7913 | -0.0311 | -20 | 172 | 184 | RLVYTNDQGEIVK |  |  |
| 1534.8224 | 1534.7913 | -0.0311 | -20 | 172 | 184 | RLVYTNDQGEIVK |  |  |
| 1630.7959 | 1630.8209 | 0.025 | 15 | 149 | 164 | LYSIASSALGDFGDSK |  |  |
| 1735.8605 | 1735.829 | -0.0315 | -18 | 331 | 346 | GMEKGIDDIMLDLAAK |  | Oxidation (M)[2] |
| 1739.8381 | 1739.823 | -0.0151 | -9 | 291 | 305 | EQTNAAGEKMYIQTR |  |  |
| 1739.8381 | 1739.823 | -0.0151 | -9 | 291 | 305 | EQTNAAGEKMYIQTR |  |  |

| **Gel Idx/Pos** | **1196** |  |  |  |  |  |  |  |
| --- | --- | --- | --- | --- | --- | --- | --- | --- |
| **Accession No.** | **Protein Name** | **Protein PI** | **Protein MW** | **Protein Score** | **Protein Score C. I. %** | **Total Ion Score** | **Total Ion C. I. %** | **Pep. Count** |
| gi|194707256 | unknown [Zea mays] | 5.96 | 33565.8 | 462 | 100 | 388 | 100 | 10 |
| **Peptide Information** | | | | | | | | |
| **Calc. Mass** | **Obsrv. Mass** | **± da** | **± ppm** | **Start Seq.** | **End Seq.** | **Sequence** | **Total Ion Score** | **Modification** |
| 946.5139 | 946.5154 | 0.0015 | 2 | 175 | 182 | DGKVCLVR |  | Carbamidomethyl (C)[5] |
| 946.5139 | 946.5154 | 0.0015 | 2 | 175 | 182 | DGKVCLVR | 46 | Carbamidomethyl (C)[5] |
| 1196.5542 | 1196.5414 | -0.0128 | -11 | 164 | 174 | AGEDQYSLASR |  |  |
| 1196.5542 | 1196.5414 | -0.0128 | -11 | 164 | 174 | AGEDQYSLASR | 71 |  |
| 1496.6976 | 1496.6742 | -0.0234 | -16 | 164 | 177 | AGEDQYSLASRDGK |  |  |
| 1564.7167 | 1564.7206 | 0.0039 | 2 | 2 | 13 | EFPHHHHHHGHR |  |  |
| 1673.9109 | 1673.7657 | -0.1452 | -87 | 202 | 216 | VKDEEGYPAIVLVNK |  |  |
| 1673.9109 | 1673.9209 | 0.01 | 6 | 202 | 216 | VKDEEGYPAIVLVNK |  |  |
| 1673.9109 | 1673.9209 | 0.01 | 6 | 202 | 216 | VKDEEGYPAIVLVNK | 65 |  |
| 1695.7571 | 1695.8625 | 0.1054 | 62 | 1 | 13 | MEFPHHHHHHGHR |  |  |
| 1760.8273 | 1760.8219 | -0.0054 | -3 | 160 | 174 | IYCKAGEDQYSLASR |  | Carbamidomethyl (C)[3] |
| 1760.8273 | 1760.8219 | -0.0054 | -3 | 160 | 174 | IYCKAGEDQYSLASR | 50 | Carbamidomethyl (C)[3] |
| 1884.8723 | 1884.8674 | -0.0049 | -3 | 237 | 252 | HNPDSLDESVLWTESR |  |  |
| 1884.8723 | 1884.8674 | -0.0049 | -3 | 237 | 252 | HNPDSLDESVLWTESR | 106 |  |
| 2048.9131 | 2048.9387 | 0.0256 | 12 | 285 | 301 | DGTALVLWEWCEGDNQR |  | Carbamidomethyl (C)[11] |
| 2165.0452 | 2165.0757 | 0.0305 | 14 | 23 | 43 | GPPPPVYGGYGQPPPPDPYGR |  |  |
| 2165.0452 | 2165.0757 | 0.0305 | 14 | 23 | 43 | GPPPPVYGGYGQPPPPDPYGR | 50 |  |

| **Gel Idx/Pos** | **1241** |  |  |  |  |  |  |  |
| --- | --- | --- | --- | --- | --- | --- | --- | --- |
| **Accession No.** | **Protein Name** | **Protein PI** | **Protein MW** | **Protein Score** | **Protein Score C. I. %** | **Total Ion Score** | **Total Ion C. I. %** | **Pep. Count** |
| gi|195643284 | chaperonin [Zea mays] | 7.74 | 25750.7 | 140 | 100 | 110 | 100 | 4 |
| **Peptide Information** | | | | | | | | |
| **Calc. Mass** | **Obsrv. Mass** | **± da** | **± ppm** | **Start Seq.** | **End Seq.** | **Sequence** | **Total Ion Score** | **Modification** |
| 801.3777 | 801.3204 | -0.0573 | -72 | 220 | 226 | YAGSEFK |  |  |
| 1004.4829 | 1004.4242 | -0.0587 | -58 | 152 | 159 | DMKPLNDR |  | Oxidation (M)[2] |
| 1135.6106 | 1135.5983 | -0.0123 | -11 | 227 | 237 | GADGTAYIVLR |  |  |
| 1135.6106 | 1135.5983 | -0.0123 | -11 | 227 | 237 | GADGTAYIVLR | 46 |  |
| 1917.9706 | 1918.0106 | 0.04 | 21 | 220 | 237 | YAGSEFKGADGTAYIVLR |  |  |
| 1917.9706 | 1918.0106 | 0.04 | 21 | 220 | 237 | YAGSEFKGADGTAYIVLR | 64 |  |
| 2563.4204 | 2563.4814 | 0.061 | 24 | 73 | 99 | TVGGILLPSTAQTKPQGG |  |  |

| **Gel Idx/Pos** | **1321** |  |  |  |  |  |  |  |
| --- | --- | --- | --- | --- | --- | --- | --- | --- |
| **Accession No.** | **Protein Name** | **Protein PI** | **Protein MW** | **Protein Score** | **Protein Score C. I. %** | **Total Ion Score** | **Total Ion C. I. %** | **Pep. Count** |
| gi|195628632 | remorin [Zea mays] | 5.74 | 21875.4 | 98 | 99.998 | 70 | 99.997 | 5 |
| **Peptide Information** | | | | | | | | |
| **Calc. Mass** | **Obsrv. Mass** | **± da** | **± ppm** | **Start Seq.** | **End Seq.** | **Sequence** | **Total Ion Score** | **Modification** |
| 804.4097 | 804.3673 | -0.0424 | -53 | 2 | 8 | AEEEAKK |  |  |
| 935.4502 | 935.4248 | -0.0254 | -27 | 1 | 8 | MAEEEAKK |  |  |
| 951.4451 | 951.4514 | 0.0063 | 7 | 1 | 8 | MAEEEAKK |  | Oxidation (M)[1] |
| 1372.6927 | 1372.7081 | 0.0154 | 11 | 70 | 82 | QGGSNDRDLALAR |  |  |
| 1372.6927 | 1372.7081 | 0.0154 | 11 | 70 | 82 | QGGSNDRDLALAR | 31 |  |
| 1419.7478 | 1419.7448 | -0.003 | -2 | 56 | 69 | VADEPAPEKPAPAK |  |  |
| 2144.196 | 2144.2253 | 0.0293 | 14 | 49 | 69 | ALAIVEKVADEPAPEKPAPAK |  |  |
| 2144.196 | 2144.2253 | 0.0293 | 14 | 49 | 69 | ALAIVEKVADEPAPEKPAPAK | 39 |  |

| **Gel Idx/Pos** | **1689** |  |  |  |  |  |  |  |
| --- | --- | --- | --- | --- | --- | --- | --- | --- |
| **Accession No.** | **Protein Name** | **Protein PI** | **Protein MW** | **Protein Score** | **Protein Score C. I. %** | **Total Ion Score** | **Total Ion C. I. %** | **Pep. Count** |
| gi|226507468 | bZIP transcription factor ABI5 [Zea mays] | 9.56 | 38416.8 | 97 | 100 | 110 | 98.856 | 9 |
| **Peptide Information** | | | | | | | | |
| **Calc. Mass** | **Obsrv. Mass** | **± da** | **± ppm** | **Start Seq.** | **End Seq.** | **Sequence** | **Total Ion Score** | **Modification** |
| 800.4335 | 800.4249 | -0.0086 | -11 | 265 | 271 | KPPAMEK |  |  |
| 800.4335 | 800.4249 | -0.0086 | -11 | 265 | 271 | KPPAMEK | 56 |  |
| 803.4118 | 803.3931 | -0.0187 | -23 | 283 | 289 | NRESAAR |  |  |
| 816.4283 | 816.3993 | -0.029 | -36 | 265 | 271 | KPPAMEK |  | Oxidation (M)[5] |
| 856.4999 | 856.5401 | 0.0402 | 47 | 337 | 344 | QVGPTAKR |  |  |
| 856.4999 | 856.5401 | 0.0402 | 47 | 336 | 343 | RQVGPTAK |  |  |
| 876.4495 | 876.4095 | -0.04 | -46 | 319 | 325 | QVEMLEK |  |  |
| 904.4523 | 904.4543 | 0.002 | 2 | 114 | 120 | TVDEVWR | 38 |  |
| 904.4523 | 904.4543 | 0.002 | 2 | 114 | 120 | TVDEVWR |  | Oxidation (M)[1] |
| 1046.5411 | 1046.5382 | -0.0029 | -3 | 328 | 335 | NEVLERMR |  |  |
| 1107.4888 | 1107.5284 | 0.0396 | 36 | 1 | 11 | MDFPGPAGSGR |  |  |

| **Gel Idx/Pos** | **1693** |  |  |  |  |  |  |  |
| --- | --- | --- | --- | --- | --- | --- | --- | --- |
| **Accession No.** | **Protein Name** | **Protein PI** | **Protein MW** | **Protein Score** | **Protein Score C. I. %** | **Total Ion Score** | **Total Ion C. I. %** | **Pep. Count** |
| gi|226507400 | aconitase [Zea mays] | 6.04 | 99565.3 | 321 | 100 | 363 | 100 | 23 |
| **Peptide Information** | | | | | | | | |
| **Calc. Mass** | **Obsrv. Mass** | **± da** | **± ppm** | **Start Seq.** | **End Seq.** | **Sequence** | **Total Ion Score** | **Modification** |
| 884.4294 | 884.4355 | 0.0061 | 7 | 871 | 877 | SFTCTLR |  | Carbamidomethyl (C)[4] |
| 900.5513 | 900.5433 | -0.008 | -9 | 49 | 56 | VLLESAIR |  |  |
| 905.5203 | 905.5178 | -0.0025 | -3 | 588 | 595 | GGKEVFLR |  |  |
| 1012.4768 | 1012.4831 | 0.0063 | 6 | 620 | 627 | VYDSIMER |  |  |
| 1028.4718 | 1028.4897 | 0.0179 | 17 | 620 | 627 | VYDSIMER |  | Oxidation (M)[6] |
| 1064.5007 | 1064.5289 | 0.0282 | 26 | 150 | 157 | NEELEFQR |  |  |
| 1104.6412 | 1104.6414 | 0.0002 | 0 | 40 | 48 | IDKLPYSIR |  |  |
| 1104.6412 | 1104.6414 | 0.0002 | 0 | 40 | 48 | IDKLPYSIR | 44 |  |
| 1140.5718 | 1140.5986 | 0.0268 | 23 | 619 | 627 | KVYDSIMER |  |  |
| 1141.6729 | 1141.6754 | 0.0025 | 2 | 81 | 90 | LAEIPFKPAR |  |  |
| 1141.6729 | 1141.6754 | 0.0025 | 2 | 81 | 90 | LAEIPFKPAR | 51 |  |
| 1178.5477 | 1178.5642 | 0.0165 | 14 | 639 | 647 | EALYPWEDR |  |  |
| 1178.5477 | 1178.5642 | 0.0165 | 14 | 639 | 647 | EALYPWEDR | 67 |  |
| 1202.6052 | 1202.6124 | 0.0072 | 6 | 71 | 80 | IIDWENTSPK |  |  |
| 1210.6355 | 1210.6174 | -0.0181 | -15 | 704 | 713 | YLLEYGVEPK |  |  |
| 1261.6019 | 1261.6437 | 0.0418 | 33 | 830 | 842 | AGEDADSLGLTGR |  |  |
| 1276.6685 | 1276.6818 | 0.0133 | 10 | 29 | 39 | FFSLPALNDPR |  |  |
| 1276.6685 | 1276.6818 | 0.0133 | 10 | 29 | 39 | FFSLPALNDPR | 46 |  |
| 1294.6274 | 1294.6548 | 0.0274 | 21 | 138 | 149 | VDVAGTYDALDR |  |  |
| 1316.678 | 1316.6925 | 0.0145 | 11 | 1 | 12 | MANPTATKHAFK |  |  |
| 1392.7271 | 1392.7382 | 0.0111 | 8 | 747 | 759 | AGPWTIHVPTGEK |  |  |
| 1513.7203 | 1513.7455 | 0.0252 | 17 | 334 | 346 | SDETVSMIEAYLR |  |  |
| 1513.7203 | 1513.7455 | 0.0252 | 17 | 334 | 346 | SDETVSMIEAYLR | 92 |  |
| 1517.7053 | 1517.7356 | 0.0303 | 20 | 350 | 361 | MFVDKHEPETER |  |  |
| 1529.7152 | 1529.7494 | 0.0342 | 22 | 334 | 346 | SDETVSMIEAYLR |  | Oxidation (M)[7] |
| 1565.8422 | 1565.8419 | -0.0003 | 0 | 477 | 491 | TSLTPGSVVATEYLK |  |  |
| 1688.9694 | 1688.8354 | -0.134 | -79 | 13 | 28 | RILTSLLKPGGGEYGK |  |  |
| 1741.996 | 1742.0239 | 0.0279 | 16 | 122 | 137 | INPLIPVDAVIDHAVR |  |  |
| 1839.964 | 1840.0135 | 0.0495 | 27 | 285 | 301 | FVEFYGVGVGELSLPAR |  |  |
| 2340.1104 | 2340.1375 | 0.0271 | 12 | 138 | 157 | VDVAGTYDALDRNEELEFQR |  |  |
| 2340.1104 | 2340.1375 | 0.0271 | 12 | 138 | 157 | VDVAGTYDALDRNEELEFQR | 63 |  |

| **Gel Idx/Pos** | **1698** |  |  |  |  |  |  |  |
| --- | --- | --- | --- | --- | --- | --- | --- | --- |
| **Accession No.** | **Protein Name** | **Protein PI** | **Protein MW** | **Protein Score** | **Protein Score C. I. %** | **Total Ion Score** | **Total Ion C. I. %** | **Pep. Count** |
| gi|149392465 | ferredoxin-nadp reductase, leaf isozyme [Oryza sativa Indica Group] | 6.59 | 21768.9 | 139 | 100 | 109 | 100 | 6 |
| **Peptide Information** | | | | | | | | |
| **Calc. Mass** | **Obsrv. Mass** | **± da** | **± ppm** | **Start Seq.** | **End Seq.** | **Sequence** | **Total Ion Score** | **Modification** |
| 809.4152 | 809.3911 | -0.0241 | -30 | 103 | 109 | VDYAVSR |  |  |
| 811.413 | 811.3856 | -0.0274 | -34 | 119 | 124 | MYIQTR |  |  |
| 811.413 | 811.3856 | -0.0274 | -34 | 119 | 124 | MYIQTR | 20 |  |
| 827.408 | 827.3798 | -0.0282 | -34 | 119 | 124 | MYIQTR |  | Oxidation (M)[1] |
| 1232.6555 | 1232.6367 | -0.0188 | -15 | 154 | 165 | GIDDIMVSLAAK |  |  |
| 1363.6022 | 1363.6902 | 0.088 | 65 | 139 | 149 | DNTYVYMCGLK |  | Carbamidomethyl (C)[8] |
| 1507.692 | 1507.7798 | 0.0878 | 58 | 138 | 149 | KDNTYVYMCGLK |  | Carbamidomethyl (C)[9], Oxidation (M)[8] |
| 1523.7601 | 1523.8147 | 0.0546 | 36 | 97 | 109 | APENFRVDYAVSR |  |  |
| 1523.7601 | 1523.8147 | 0.0546 | 36 | 97 | 109 | APENFRVDYAVSR | 89 |  |

| **Gel Idx/Pos** | **1732** |  |  |  |  |  |  |  |
| --- | --- | --- | --- | --- | --- | --- | --- | --- |
| **Accession No.** | **Protein Name** | **Protein PI** | **Protein MW** | **Protein Score** | **Protein Score C. I. %** | **Total Ion Score** | **Total Ion C. I. %** | **Pep. Count** |
| gi|195619268 | peroxiredoxin-5 [Zea mays] | 7.7399 | 23917.6 | 110 | 100 | 85 | 95 | 5 |
| **Peptide Information** | | | | | | | | |
| **Calc. Mass** | **Obsrv. Mass** | **± da** | **± ppm** | **Start Seq.** | **End Seq.** | **Sequence** | **Total Ion Score** | **Modification** |
| 868.504 | 868.4453 | -0.0587 | -68 | 124 | 131 | HLPGFVAK |  |  |
| 1177.6464 | 1177.6533 | 0.0069 | 6 | 201 | 211 | YALLAEDGVVK |  |  |
| 1177.6464 | 1177.6533 | 0.0069 | 6 | 201 | 211 | YALLAEDGVVK | 30 |  |
| 1333.7474 | 1333.7599 | 0.0125 | 9 | 200 | 211 | RYALLAEDGVVK |  |  |
| 1333.7474 | 1333.7599 | 0.0125 | 9 | 200 | 211 | RYALLAEDGVVK |  |  |
| 1871.9896 | 1872.0991 | 0.1095 | 58 | 180 | 197 | AMGVELDLSDKPVGLGVR |  | Oxidation (M)[2] |
| 1871.9896 | 1872.0991 | 0.1095 | 58 | 180 | 197 | AMGVELDLSDKPVGLGVR | 65 | Oxidation (M)[2] |

| **Gel Idx/Pos** | **1985** |  |  |  |  |  |  |  |
| --- | --- | --- | --- | --- | --- | --- | --- | --- |
| **Accession No.** | **Protein Name** | **Protein PI** | **Protein MW** | **Protein Score** | **Protein Score C. I. %** | **Total Ion Score** | **Total Ion C. I. %** | **Pep. Count** |
| gi|195640294 | thioredoxin F-type [Zea mays] | 8.88 | 21062.1 | 78 | 99.84 | 63 | 99.985 | 3 |
| **Peptide Information** | | | | | | | | |
| **Calc. Mass** | **Obsrv. Mass** | **± da** | **± ppm** | **Start Seq.** | **End Seq.** | **Sequence** | **Total Ion Score** | **Modification** |
| 947.556 | 947.5222 | -0.0338 | -36 | 139 | 146 | NLDVVFLK |  |  |
| 1428.7581 | 1428.6979 | -0.0602 | -42 | 185 | 197 | IDELAEAIEIANK |  |  |
| 1556.853 | 1556.8086 | -0.0444 | -29 | 185 | 198 | IDELAEAIEIANKK |  |  |
| 1556.853 | 1556.8086 | -0.0444 | -29 | 185 | 198 | IDELAEAIEIANKK | 63 |  |

| **Gel Idx/Pos** | **2897** |  |  |  |  |  |  |  |
| --- | --- | --- | --- | --- | --- | --- | --- | --- |
| **Accession No.** | **Protein Name** | **Protein PI** | **Protein MW** | **Protein Score** | **Protein Score C. I. %** | **Total Ion Score** | **Total Ion C. I. %** | **Pep. Count** |
| gi|195655243 | abscisic stress ripening protein 1 [Zea mays] | 6.81 | 11666.8 | 234 | 100 | 193 | 100 | 5 |
| **Peptide Information** | | | | | | | | |
| **Calc. Mass** | **Obsrv. Mass** | **± da** | **± ppm** | **Start Seq.** | **End Seq.** | **Sequence** | **Total Ion Score** | **Modification** |
| 962.4625 | 962.4984 | 0.0359 | 37 | 33 | 39 | HHKHMEK |  | Oxidation (M)[5] |
| 1218.561 | 1218.5813 | 0.0203 | 17 | 61 | 70 | KDPENEHGHR |  |  |
| 1218.561 | 1218.5813 | 0.0203 | 17 | 61 | 70 | KDPENEHGHR | 53 |  |
| 1257.625 | 1257.6365 | 0.0115 | 9 | 6 | 14 | HHHHLFHHR |  |  |
| 1683.9065 | 1683.9541 | 0.0476 | 28 | 40 | 56 | LGELGAIAAGAYALHEK |  |  |
| 1683.9065 | 1683.9541 | 0.0476 | 28 | 40 | 56 | LGELGAIAAGAYALHEK | 140 |  |
| 2520.2629 | 2520.363 | 0.1001 | 40 | 71 | 94 | VKEEVAAVAAVGSAGFAFHEHHEK |  |  |
| 2520.2629 | 2520.363 | 0.1001 | 40 | 71 | 94 | VKEEVAAVAAVGSAGFAFHEHHEK |  |  |

| **Gel Idx/Pos** | **1797** |  |  |  |  |  |  |  |
| --- | --- | --- | --- | --- | --- | --- | --- | --- |
| **Accession No.** | **Protein Name** | **Protein PI** | **Protein MW** | **Protein Score** | **Protein Score C. I. %** | **Total Ion Score** | **Total Ion C. I. %** | **Pep. Count** |
| gi|226496743 | 50S ribosomal protein L1 [Zea mays] | 8.69 | 37235.2 | 714 | 100 | 567 | 100 | 16 |
| **Peptide Information** | | | | | | | | |
| **Calc. Mass** | **Obsrv. Mass** | **± da** | **± ppm** | **Start Seq.** | **End Seq.** | **Sequence** | **Total Ion Score** | **Modification** |
| 936.4533 | 936.4403 | -0.013 | -14 | 161 | 167 | YNDQQLR |  |  |
| 936.4533 | 936.4403 | -0.013 | -14 | 161 | 167 | YNDQQLR | 42 |  |
| 958.5568 | 958.5397 | -0.0171 | -18 | 182 | 190 | IAVLTQGEK |  |  |
| 1046.6357 | 1046.6147 | -0.021 | -20 | 115 | 122 | FLEIQKLR |  |  |
| 1054.6045 | 1054.5798 | -0.0247 | -23 | 275 | 284 | SGIVHIPFGK |  |  |
| 1102.5635 | 1102.5914 | 0.0279 | 25 | 221 | 230 | LIASPDMMPK |  |  |
| 1170.6378 | 1170.6414 | 0.0036 | 3 | 87 | 97 | GPAFTAPTRPR |  |  |
| 1170.6378 | 1170.6414 | 0.0036 | 3 | 87 | 97 | GPAFTAPTRPR | 50 |  |
| 1192.5746 | 1192.5806 | 0.006 | 5 | 145 | 154 | FVESAEAHFR |  |  |
| 1192.5746 | 1192.5806 | 0.006 | 5 | 145 | 154 | FVESAEAHFR | 73 |  |
| 1396.7948 | 1396.7623 | -0.0325 | -23 | 272 | 284 | VDKSGIVHIPFGK |  |  |
| 1480.7716 | 1480.7678 | -0.0038 | -3 | 126 | 138 | KDYDVPTAISLMK |  |  |
| 1542.8486 | 1542.8802 | 0.0316 | 20 | 182 | 195 | IAVLTQGEKIDEAR |  |  |
| 1542.8486 | 1542.8802 | 0.0316 | 20 | 182 | 195 | IAVLTQGEKIDEAR | 101 |  |
| 1634.7955 | 1634.8448 | 0.0493 | 30 | 155 | 167 | MNLDPKYNDQQLR |  |  |
| 1694.8596 | 1694.8516 | -0.008 | -5 | 249 | 264 | AGTVSSNITQAIEEFK |  |  |
| 1698.8909 | 1698.9025 | 0.0116 | 7 | 196 | 212 | AAGADIVGGEDLIEQIK |  |  |
| 1698.8909 | 1698.9025 | 0.0116 | 7 | 196 | 212 | AAGADIVGGEDLIEQIK | 92 |  |
| 1726.8204 | 1726.8129 | -0.0075 | -4 | 331 | 345 | LNIKEMLDYGLESSD |  |  |
| 1822.9546 | 1822.9646 | 0.01 | 5 | 249 | 265 | AGTVSSNITQAIEEFKK |  |  |
| 1822.9546 | 1822.9646 | 0.01 | 5 | 249 | 265 | AGTVSSNITQAIEEFKK | 86 |  |
| 1840.8647 | 1840.8828 | 0.0181 | 10 | 139 | 154 | QMSSAKFVESAEAHFR |  | Oxidation (M)[2] |
| 1980.9736 | 1981.0502 | 0.0766 | 39 | 285 | 301 | VDFPEEDLIANFMSVVR |  |  |
| 1980.9736 | 1981.0502 | 0.0766 | 39 | 285 | 301 | VDFPEEDLIANFMSVVR | 122 |  |
| 1996.9685 | 1996.991 | 0.0225 | 11 | 285 | 301 | VDFPEEDLIANFMSVVR |  | Oxidation (M)[13] |

| **Gel Idx/Pos** | **1802** |  |  |  |  |  |  |  |
| --- | --- | --- | --- | --- | --- | --- | --- | --- |
| **Accession No.** | **Protein Name** | **Protein PI** | **Protein MW** | **Protein Score** | **Protein Score C. I. %** | **Total Ion Score** | **Total Ion C. I. %** | **Pep. Count** |
| gi|50952841 | RuBisCo subunit binding-protein beta subunit [Zea mays] | 4.75 | 44275 | 450 | 100 | 345 | 100 | 14 |
| **Peptide Information** | | | | | | | | |
| **Calc. Mass** | **Obsrv. Mass** | **± da** | **± ppm** | **Start Seq.** | **End Seq.** | **Sequence** | **Total Ion Score** | **Modification** |
| 899.5421 | 899.5203 | -0.0218 | -24 | 303 | 310 | VGAEIVRR |  |  |
| 936.4785 | 936.4668 | -0.0117 | -12 | 334 | 341 | VLSNDNFK |  |  |
| 947.5672 | 947.5438 | -0.0234 | -25 | 310 | 317 | RALSYPLK |  |  |
| 1229.7001 | 1229.6954 | -0.0047 | -4 | 136 | 147 | IAAIKAPGFGER |  |  |
| 1229.7001 | 1229.6954 | -0.0047 | -4 | 136 | 147 | IAAIKAPGFGER | 42 |  |
| 1245.5715 | 1245.6232 | 0.0517 | 42 | 368 | 378 | CCLEHAASVAK |  | Carbamidomethyl (C)[1,2] |
| 1284.7158 | 1284.7261 | 0.0103 | 8 | 95 | 105 | DLINVLEEAIR |  |  |
| 1284.7158 | 1284.7261 | 0.0103 | 8 | 95 | 105 | DLINVLEEAIR | 97 |  |
| 1505.7159 | 1505.7134 | -0.0025 | -2 | 61 | 73 | GYISPYFVTDSEK |  |  |
| 1693.8279 | 1693.8313 | 0.0034 | 2 | 214 | 227 | NLIEAAEQEYEKEK |  |  |
| 1700.9 | 1700.9456 | 0.0456 | 27 | 268 | 284 | AAVEEGIVVGGGCTLLR |  | Carbamidomethyl (C)[13] |
| 1700.9 | 1700.9456 | 0.0456 | 27 | 268 | 284 | AAVEEGIVVGGGCTLLR | 124 | Carbamidomethyl (C)[13] |
| 1884.0437 | 1883.9796 | -0.0641 | -34 | 235 | 253 | LAGGVAVIQVGAQTETELK |  |  |
| 1920.8796 | 1920.9535 | 0.0739 | 38 | 45 | 60 | SSENFLYVVEGMQFDR |  |  |
| 1920.8796 | 1920.9535 | 0.0739 | 38 | 45 | 60 | SSENFLYVVEGMQFDR | 45 |  |
| 1936.9459 | 1936.9326 | -0.0133 | -7 | 191 | 208 | ESTTIVGDGSTQEEVTKR |  |  |
| 1936.9459 | 1936.9326 | -0.0133 | -7 | 191 | 208 | ESTTIVGDGSTQEEVTKR | 41 |  |
| 2141.1812 | 2141.1406 | -0.0406 | -19 | 235 | 255 | LAGGVAVIQVGAQTETELKEK |  |  |
| 2462.1545 | 2462.1809 | 0.0264 | 11 | 342 | 364 | YGYNAATGQYEDLMAAGIIDPTK |  |  |
| 2478.1494 | 2478.2334 | 0.084 | 34 | 342 | 364 | YGYNAATGQYEDLMAAGIIDPTK |  | Oxidation (M)[14] |

| **Gel Idx/Pos** | **1826** |  |  |  |  |  |  |  |
| --- | --- | --- | --- | --- | --- | --- | --- | --- |
| **Accession No.** | **Protein Name** | **Protein PI** | **Protein MW** | **Protein Score** | **Protein Score C. I. %** | **Total Ion Score** | **Total Ion C. I. %** | **Pep. Count** |
| gi|195637254 | nucleoside diphosphate kinase 2 [Zea mays] | 9.51 | 24181.7 | 355 | 100 | 250 | 100 | 12 |
| **Peptide Information** | | | | | | | | |
| **Calc. Mass** | **Obsrv. Mass** | **± da** | **± ppm** | **Start Seq.** | **End Seq.** | **Sequence** | **Total Ion Score** | **Modification** |
| 878.4771 | 878.5054 | 0.0283 | 32 | 127 | 133 | DKPFFPK |  |  |
| 878.4771 | 878.5054 | 0.0283 | 32 | 127 | 133 | DKPFFPK | 10 |  |
| 916.4847 | 916.5133 | 0.0286 | 31 | 176 | 184 | GDLAVQTGR |  |  |
| 916.4847 | 916.5133 | 0.0286 | 31 | 176 | 184 | GDLAVQTGR | 58 |  |
| 943.5571 | 943.5764 | 0.0193 | 20 | 89 | 97 | GLVGEIISR |  |  |
| 964.5138 | 964.5283 | 0.0145 | 15 | 199 | 205 | EIELWFK |  |  |
| 1003.4843 | 1003.5102 | 0.0259 | 26 | 116 | 123 | DLAQEHYK |  |  |
| 1359.6903 | 1359.7103 | 0.02 | 15 | 116 | 126 | DLAQEHYKDLK |  |  |
| 1406.7461 | 1406.7496 | 0.0035 | 2 | 77 | 88 | SYIMIKPDGVQR |  |  |
| 1406.7461 | 1406.7496 | 0.0035 | 2 | 77 | 88 | SYIMIKPDGVQR | 17 |  |
| 1422.741 | 1422.7291 | -0.0119 | -8 | 77 | 88 | SYIMIKPDGVQR |  | Oxidation (M)[4] |
| 1422.741 | 1422.7291 | -0.0119 | -8 | 77 | 88 | SYIMIKPDGVQR | 23 | Oxidation (M)[4] |
| 1481.7092 | 1481.6986 | -0.0106 | -7 | 185 | 198 | NVVHGSDSPDNGKR |  |  |
| 1481.7092 | 1481.6986 | -0.0106 | -7 | 185 | 198 | NVVHGSDSPDNGKR | 21 |  |
| 1540.8442 | 1540.7897 | -0.0545 | -35 | 63 | 76 | AVPQRIVASSEVER |  |  |
| 1650.9174 | 1650.916 | -0.0014 | -1 | 160 | 175 | LIGATNPLQAEPGTIR |  |  |
| 1650.9174 | 1650.916 | -0.0014 | -1 | 160 | 175 | LIGATNPLQAEPGTIR | 66 |  |
| 1779.0123 | 1779.0095 | -0.0028 | -2 | 159 | 175 | KLIGATNPLQAEPGTIR |  |  |
| 1779.0123 | 1779.0095 | -0.0028 | -2 | 159 | 175 | KLIGATNPLQAEPGTIR | 71 |  |
| 2548.3843 | 2548.3459 | -0.0384 | -15 | 160 | 184 | LIGATNPLQAEPGTIRGDLAVQTGR |  |  |
| 2548.3843 | 2548.3459 | -0.0384 | -15 | 160 | 184 | LIGATNPLQAEPGTIRGDLAVQTGR | 2 |  |
| 2548.3843 | 2548.5947 | 0.2104 | 83 | 160 | 184 | LIGATNPLQAEPGTIRGDLAVQTGR |  |  |
